# On a Grover-Based Quantum Signature Scheme and a Teleportation-Based Design

**DOI:** 10.3390/e28091030

**Published:** 2026-09-18

**Authors:** Guoliang Xu, Songyao Xue, Xiangfu Zou, Yumei Zhang

**Affiliations:** 1 School of Software, Luoyang Normal University, Luoyang 471934, China; 231164193@lynu.edu.cn; 2 School of Mathematics and Computational Science, Wuyi University, Jiangmen 529020, China; 3 Conservatory of Music, Luoyang Normal University, Luoyang 471934, China

**Keywords:** quantum computation, Grover algorithm, quantum signature, security analysis

## Abstract

Quantum computation, with tools including Grover’s algorithm, quantum walks, and quantum teleportation, plays an important role in quantum signature designs. Although such designs offer signature functionality, they sometimes come at the cost of security. This paper first reviews a Grover-based scheme and shows how man-in-the-middle attacks enable complete key recovery, Alice’s disavowal, and Bob’s forgery. We then propose an arbitrated quantum signature scheme based on quantum teleportation and a strengthened quantum one-time pad. The scheme is designed for quantum messages with known classical descriptions, not for arbitrary unknown quantum states. The security of the proposed scheme is analyzed through a formal adversarial model for unforgeability and non-repudiation, and its resilience against replay, intercept-and-resend, man-in-the-middle, entanglement, and collective or coherent attacks is examined. Numerical simulations and an asymptotic resource analysis further illustrate the practicality of the scheme.

## 1. Introduction

In the field of quantum computation and communication, Grover’s algorithm, quantum teleportation and quantum walk have inspired a variety of cryptographic constructions far beyond their original application scenarios [1,2,3,4,5,6,7]. Leveraging quantum superposition, phase manipulation and iterative evolution, these algorithm-driven schemes feature concise and flexible architectures, and offer security guarantees based on quantum information-theoretic principles. Among these quantum primitives, Grover’s algorithm, as a fundamental building block in quantum computation and complexity theory, is particularly intriguing and conceptually appealing, offering a novel perspective for the design and construction of quantum signature schemes.

Among quantum signature paradigms, arbitrated quantum signature (AQS) schemes introduce a trusted or semi-trusted arbitrator to assist verification and dispute resolution [8,9,10]. Since the early scheme of Zeng and Keitel [8], a range of AQS constructions have been proposed using entanglement, Bell states, or quantum one-time pad (QOTP) encryption [9,11,12,13]. A number of schemes built from seemingly sound quantum primitives were later shown to admit forgery, repudiation, or impersonation attacks [14,15,16]. These results suggest that the security of quantum signature schemes depends not only on the local soundness of the primitives employed, but also on the way those primitives are combined at the protocol level.

In 2015, Yoon et al. [4] proposed a quantum signature scheme that adopts Grover-type operations for signature generation and verification. Later in 2023, Yoon et al. [6] further extended this line of research by presenting a quantum asymmetric key cryptographic scheme based on Grover iteration. Their work migrated Grover-related operations from quantum signature scenarios to the domain of quantum asymmetric encryption, further enriching the application boundaries of Grover primitives in quantum cryptography. From the algorithmic perspective, such designs are conceptually appealing since they embed standard quantum search routines directly into cryptographic workflows. Nevertheless, combining reversible quantum transformations with publicly accessible verification parameters leads to an inherent structural conflict: information that is harmless in standard quantum search scenarios may become a critical security vulnerability in signature and encryption systems.

In this paper, we study quantum signature security from that structural perspective. Our main contributions are as follows:

(1) We analyze the Grover-based quantum signature scheme of Yoon et al. [4] and demonstrate that it is vulnerable to man-in-the-middle attacks, which enable complete key recovery, Alice’s disavowal, and Bob’s existential forgery.
(2) We propose an arbitrated quantum signature scheme based on quantum teleportation and a strengthened QOTP [16]. The scheme is designed for quantum messages with known classical descriptions, not for arbitrary unknown quantum states. A formal security analysis under a well-defined adversarial model shows that the scheme satisfies unforgeability and non-repudiation, and numerical simulations illustrate its practical viability.

The remainder of this paper is organized as follows. Section 2 introduces the necessary preliminaries. Section 3 reviews and analyzes the Grover-based quantum signature scheme of Yoon et al. [4]. Section 4 proposes and analyzes an arbitrated quantum signature scheme that combines quantum teleportation and strengthened QOTP [16]. Section 5 provides a resource overhead analysis of the proposed scheme and YKLY’2015, and simulations of the attacks on the proposed scheme. Finally, Section 6 concludes the paper. The complete simulation codes are provided in the Supplementary Materials.

## 2. Preliminaries

This section introduces the notation, quantum primitives, and background used throughout the paper.

For convenience, the principal symbols used throughout the paper are summarized in Table 1.

Then, Grover’s algorithm provides a quadratic speedup for unstructured search by iteratively amplifying the amplitude of marked states [5]. In Grover-based quantum signature schemes [4], the mechanism of unitary operations is represented by $U_{S}=I-2|K\rangle \langle K|$, $U_{V}=2|M\rangle \langle M|-I$, and their combined action $U_{V}U_{S}|M\rangle =a|K\rangle$. Here, applying the unitary operators $U_{S}$ and $U_{V}$, this mechanism can be used to identify the desired data $|K\rangle$ (for example $|K\rangle =|00\rangle$) from database $|M\rangle$ (for example, $|M\rangle =\frac{1}{2}\left(|00\rangle +|01\rangle +|10\rangle +|11\rangle \right)$). The Grover-based quantum signature scheme [4] was proposed by utilizing the unitary operations $U_{V}$ and $U_{S}$.

The teleportation procedure used in our protocol proceeds as follows. Alice combines the message state |p〉=α|0〉+β|1〉 ($\alpha ,\beta \in {C,|\alpha |}^{2}+{\left|\beta \right|}^{2}=1$) with the Bell state ${|\varphi^{+}\rangle }_{AB}$ to obtain a three-particle entangled state

(1) $$\begin{array}{cc} {|p\rangle }_{A}⊗{|\varphi^{+}\rangle }_{AB} & =\frac{1}{2}{[|\varphi^{+}\rangle }_{AA}(\alpha |0\rangle +{\beta |1\rangle )}_{B}+{|\varphi^{-}\rangle }_{AA}(\alpha |0\rangle -{\beta |1\rangle )}_{B}\\ \; & +{|\psi^{+}\rangle }_{AA}(\alpha |1\rangle +{\beta |0\rangle )}_{B}+{|\psi^{-}\rangle }_{AA}(\alpha |1\rangle -{\beta |0\rangle )}_{B}]. \end{array}$$

Defining U(X) as

(2) $$U(X)=\left\{\begin{array}{cc} I\;\;\;\;\; & =|0\rangle \langle 0|+|1\rangle \langle 1|,\;X=00,\\ \delta_{x}\;\;\; & =|1\rangle \langle 0|+|0\rangle \langle 1|,\;X=01,\\ \delta_{z}\;\;\; & =|0\rangle \langle 0|-|1\rangle \langle 1|,\;X=10,\\ \delta_{z}\delta_{x} & =|0\rangle \langle 1|-|1\rangle \langle 0|,\;X=11. \end{array}\right.$$

the above state ${|p\rangle }_{A}⊗{|\varphi^{+}\rangle }_{AB}$ can be compactly written as

(3) $${|p\rangle }_{A}⊗{|\varphi^{+}\rangle }_{AB}=\frac{1}{2}\sum_{X=00}^{11}\left[I⊗U\left(X\right)\right]{|\varphi^{+}\rangle }_{AA}⊗U\left(X\right){|p\rangle }_{B},$$

where each $\left[I⊗U\left(X\right)\right]|\varphi^{+}\rangle$ is one of the four Bell states. Alice then performs a Bell measurement on her two particles. The state collapses to

(4) $$\left[I⊗U\left(X\right)\right]{|\varphi^{+}\rangle }_{AA}⊗U\left(X\right){|p\rangle }_{B},$$

with *X* uniformly distributed over {00,01,10,11}. Alice obtains *X* from the Bell measurement outcome, while Bob’s particle collapses to U(X)|p〉. We refer to this process as Alice teleporting U(X)|p〉 to Bob.

The strengthened quantum one-time pad (QOTP) proposed by Kim et al. [16] addresses forgeability issues present in standard QOTP-based arbitrated quantum signature schemes [14,15]. Using a 4-bit key *K*, a single-qubit message |p〉=α|0〉+β|1〉 is encrypted as

(5) $$|q\rangle =E_{K}|p\rangle =\delta_{x}^{K^{1}}\delta_{z}^{K^{2}}T\delta_{x}^{K^{3}}\delta_{z}^{K^{4}}|p\rangle ,$$

where $T=\frac{i}{\sqrt{3}}\left(\delta_{x}-\delta_{y}+\delta_{z}\right)$. The standard QOTP achieves information-theoretic security when keys are uniformly random and never reused [17,18]. Our protocol inherits this property by generating fresh keys for each execution.

## 3. Analysis of a Grover-Based Quantum Signature Scheme (YKLY’2015)

In this section, we analyze the quantum signature scheme based on Grover’s search algorithm proposed by Yoon, Kang, Lim, and Yang (YKLY’2015) [4]. We show that the scheme suffers from fundamental structural vulnerabilities that lead to complete key recovery and universal forgery.

### 3.1. Review of a Grover-Based Quantum Signature Scheme (YKLY’2015)

In the interest of readability, we briefly review YKLY’2015 [4] in this section. The scheme consists of two communication processes: between Alice and TC, and between TC and Bob. Meanwhile, both processes consist of three phases: preparation, signature and verification. YKLY’2015 [4] can be described as follows.

#### 3.1.1. Process Between Alice and TC

The preparation phase consists of the following two steps.

(AT1) Alice announces the classical message *m* that she wants to send to Bob on the public board. Then, Alice and TC share the secret key $K_{AT}$ using a QKD protocol, such as those in Refs. [19,20,21], which provides unconditional security [22,23].

(AT2) Alice creates the signing unitary operator $U_{S_{AT}}=I-2|K_{AT}\rangle \langle K_{AT}|$ where $|K_{AT}\rangle$ is a two-qubit state generated from the classical two-bit key $K_{AT}$. For example, $|K_{AT}\rangle =|ij\rangle$ if $K_{AT}=ij$ for all i,j∈{0,1}.

In the Signature phase, Alice makes her signature in Step (AT3) and sends it to TC by Steps (AT4)-(AT7).

(AT3) Using the classical message *m*, Alice generates an original message qubit |M〉 where |M〉=|−〉⊗|−〉 if m=00, |M〉=|−〉⊗|+〉 if m=01, |M〉=|+〉⊗|−〉 if m=10, and |M〉=|+〉⊗|+〉 if m=11. Then, she obtains the signature $U_{S_{AT}}|M\rangle =(I-2|K_{AT}\rangle \langle K_{AT}|)(|M\rangle )$ using |M〉 and $U_{S_{AT}}$.

(AT4) Alice includes decoy qubits of the EPR pair, $|\psi^{-}\rangle =\frac{1}{\sqrt{2}}(|01\rangle -\left|10\rangle \right)$, in random locations among the message qubits $U_{S_{AT}}|M\rangle$. Alice sends TC the first sequence of particles of the message qubits, and tells TC the locations of half the decoy qubits.

(AT5) TC performs measurement on the decoy qubits using *Z* or *X* basis, and tells Alice his measurement bases and measurement outcomes. Alice uses the same measuring bases to measure the corresponding qubits in the sequence and compares the measurement outcomes with TC’s. If the error rate is smaller than the channel average error rate, Alice and TC conclude that there are no eavesdroppers in the channel and continue the protocol.

(AT6) Alice performs random unitary operations chosen from {I,X,Y,Z} on the other half of the decoy qubits. She sends the other sequence of the particles to TC, and informs TC the positions of decoy qubits and the types of unitary operations she has chosen.

(AT7) TC confirms the security of the communication channel based on the received information.

In the Verification phase, there is only one step.

(AT8) TC first makes the verifying unitary operator $U_{V}=2|M\rangle \langle M|-I$ based on the known message *m*. Then, TC implements $U_{V}$ on the received message to recover the shared key $K_{AT}$ as $U_{V}U_{S_{AT}}\left|M\rangle =\pm \right|K_{AT}\rangle .$ Finally, TC compares the result of $U_{V}U_{S_{AT}}|M\rangle$ with the shared key. If they are identical, TC confirms that Alice is a legitimate signer.

#### 3.1.2. Process Between TC and Bob

Confirming that the received message was legitimately signed by Alice, TC sends the message, his signature, to Bob by repeating the same processes above. By this process, Bob can ensure that the message received from Alice, in fact, the channel, is signed by her.

In the Preparation phase, there are the following two steps.

(TB1) TC and Bob share the secret key $K_{TB}$ by a QKD protocol, such as those in Refs. [19,20,21], which can provide unconditional security [22,23].

(TB2) TC constructs the signing unitary operator $U_{S_{TB}}=I-2|K_{TB}\rangle \langle K_{TB}|$ where $|K_{TB}\rangle$ is a two-bit state generated from $K_{TB}$ as with $K_{AT}$.

In the Signature phase, TC makes his signature in Step (TB3) and sends it to Bob by Steps (TB4)–(TB7).

(TB3) TC signs the message |M〉 using $U_{S_{TB}}$ as $U_{S_{TB}}|M\rangle =(I-2|K_{TB}\rangle \langle K_{TB}|)(|M\rangle ).$

(TB4) TC includes decoy qubits of the EPR pair, $|\psi^{-}\rangle =\frac{1}{\sqrt{2}}(|01\rangle -\left|10\rangle \right)$, in random locations among the message qubits $U_{S_{TB}}|M\rangle$. TC sends Bob the first sequence of particles of the message qubits, and informs Bob of the locations of half the decoy qubits.

(TB5) Bob measures the decoy qubits in the *Z* or *X* basis, and tells TC his measurement bases and measurement outcomes. TC uses the same measuring bases to measure the corresponding decoy qubits in the sequence and compares the measurement outcomes with those of Bob. If the error rate is smaller than the channel average error rate, TC and Bob conclude that there are no eavesdroppers in the channel, and continue the protocol.

(TB6) TC performs unitary operations chosen from {I,X,Y,Z} on the other half of the decoy qubits. TC sends the other sequence of particles to Bob, and informs him the positions of decoy qubits and the types of unitary operations he has chosen.

(TB7) Bob confirms the security of the communication channel based on the information.

In the Verification phase, there is only one step.

(TB8) Bob makes the verifying unitary operator $U_{V}=2|M\rangle \langle M|-I$ based on the known message *m*. Bob then applies $U_{V}$ on the received message to recover the shared key $K_{TB}$ as $U_{V}U_{S_{TB}}\left|M\rangle =\pm \right|K_{TB}\rangle .$ Bob compares the result of $U_{V}U_{S_{TB}}|M\rangle$ with the shared key. If they are identical, Bob confirms that TC is a legitimate signer.

### 3.2. Man-in-the-Middle (MITM) Attacks

In YKLY’2015 [4], TC serves as the arbitrator and plays a crucial role in the protocol. When a dispute arises between Alice and Bob, only TC has the authority to resolve disputes. In this section, we first give a MITM attack on YKLY’2015 [4]. Then, we show how Alice uses the proposed MITM attack to disavow signature and Bob uses the proposed MITM attack to forge signature.

#### 3.2.1. Eve’s MITM Attack

**Figure 1:**
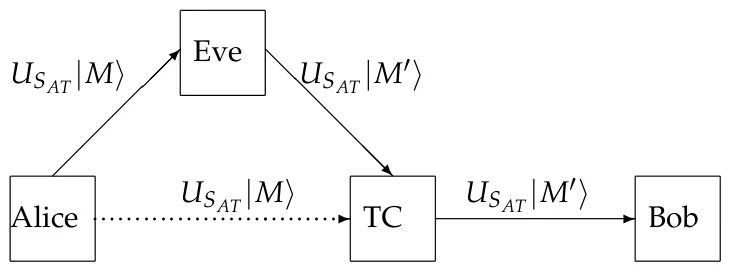
MITM attack. Solid lines correspond to the process of the attack while the dotted line denotes the corresponding process of YKLY’2015 [4].

The MITM attack on YKLY’2015 [4] consists of three processes: between Alice and Eve, between Eve and TC, and between TC and Bob. In this attack, Eve completely intercepts and replaces the quantum channel between Alice and TC, impersonating TC to Alice and Alice to TC. The attack is illustrated in Figure 1 and we present it in detail as follows.

In the process between Alice and Eve, the attacker Eve first intercepts the message qubits $U_{S_{AT}}|M\rangle$ to obtain the shared key $K_{AT}$ by pretending to be TC and performing the following four steps.

(AE1) In Step (AT4), Eve intercepts the first sequence of particles of the message qubits $U_{S_{AT}}|M\rangle$ and the message specifying the locations of half of the decoy qubits.

(AE2) In Step (AT5), Eve measures on the decoy qubits in the *Z* or *X* basis, and impersonates TC to tell Alice her measurement bases and measurement outcomes.

(AE3) In Step (AT6), Eve intercepts the other sequence of the particles that Alice sends to TC and the information specifying the positions of the decoy qubits and the selected unitary operations.

(AE4) In Step (AT8), Eve makes the unitary operator $U_{V}=2|M\rangle \langle M|-I$ based on the known message. Then, she implements $U_{V}$ on the received message to recover the shared key as $U_{V}U_{S_{AT}}\left|M\rangle =\pm \right|K_{AT}\rangle .$ Thereby, Eve can obtain the shared key $K_{AT}$ successfully.

In the Process between Eve and TC, Eve performs the following two steps to pass TC’s verification by posing as Alice.

(ET1) Eve pretends to be Alice to announce forged message $m^{\prime }$ and makes its signature $U_{S_{AT}}|M^{\prime }\rangle$ with $K_{AT}$.

(ET2) Eve impersonates Alice to communicate with TC by performing Steps (AT4)–(AT8).

Finally, there is nothing Eve needs to do in the process between TC and Bob.

By performing the MITM attack above, $U_{S_{AT}}|M^{\prime }\rangle$ as the signature of the forged message $m^{\prime }$ can pass TC’s verification. Therefore, Eve can forge Alice’s signature for any message successfully.

**Remark** **1.** *Similarly, to forge Alice’s signature in YKLY’2015 [4], Eve can perform an MITM attack between TC and Bob: Eve intercepts the message qubits $U_{S_{TB}}|M\rangle$ to obtain the shared key $K_{TB}$, pretends to be Alice to announce forged message $m^{\prime }$, makes the forged signature $U_{S_{TB}}|M^{\prime }\rangle$ with $K_{TB}$, and sends $U_{S_{TB}}|M^{\prime }\rangle$ to Bob as TC in YKLY’2015.*

#### 3.2.2. Alice’s Disavowal Attack

**Figure 2:**
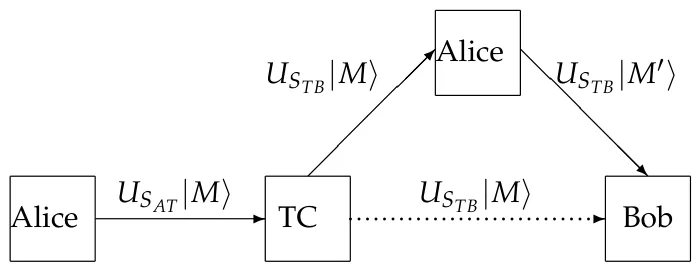
Alice’s disavowal attack. Solid lines correspond to the process of the attack while the dotted line denotes the corresponding process of YKLY’2015 [4].

Alice can achieve her disavowal in YKLY’2015 [4] by performing an MITM attack between TC and Bob which consists of two processes: between TC and Alice, and between Alice and Bob. The MITM attack is described in Figure 2.

Its details will be shown in the following.

In the Process between TC and Alice, Alice first pretends to be Bob and performs the following four steps.

(TA1) In Step (TB4), Alice intercepts the first sequence of particles of the message qubits $U_{S_{TB}}|M\rangle$ and the message that TC tells Bob the locations of half the decoy qubits.

(TA2) In Step (TB5), Alice performs measurement on the decoy qubits using *Z* or *X* basis, and impersonates Bob to tell TC her measurement bases and measurement outcomes.

(TA3) In Step (TB6), Alice intercepts the other sequence of the particles that TC sends to Bob and the message that TC tells Bob the positions of decoy qubits and the types of unitary operations.

(TA4) In Step (TB8), Alice makes a unitary operator $U_{V}=2|M\rangle \langle M|-I$ based on the known message. She implements the operator $U_{V}$ on the received message to recover the shared key as $U_{V}U_{S_{TB}}\left|M\rangle =\pm \right|K_{TB}\rangle .$ At this point, Alice has got the shared key $K_{TB}$.

In the Process between Alice and Bob, Alice poses as TC to perform the following two steps to cause Bob to accept her forged signature.

(AB1) Alice announces the forged message $m^{\prime }$ and makes the forged TC’s signature $U_{S_{TB}}|M^{\prime }\rangle$ with $K_{TB}$.

(AB2) Alice communicates with Bob to perform Steps (TB4)–(TB8) by posing as TC.

By performing operations above, Alice can forge a message and its signature which can pass Bob’s verification. Note that the sources of the messages received by Bob cannot be distinguished. In fact, the message which TC has verified and the message which Bob has received are not the same one in Alice’s attack. As a result, Alice can announce that the *m* is not the initial message to achieve her benefit.

#### 3.2.3. Bob’s Forgery Attack

Bob’s forgery attack follows the same pattern as Eve’s MITM attack, applied to the TC-Bob phase. As the legitimate receiver, Bob already possesses the correct position to intercept the message qubits $U_{S_{TB}}|M\rangle$ sent from TC to Bob in Steps (TB4)–(TB7). Bob extracts the shared key $K_{TB}$ using the publicly known verifying operator $U_{V}=2|M\rangle \langle M|-I$, which he can construct from the message *m* announced on the public board. With $K_{TB}$ in hand, Bob can sign any forged message $m^{\prime }$ by preparing $U_{S_{TB}}|M^{\prime }\rangle$ and present it as evidence that the message originated from Alice and was verified by TC. Since TC’s verification in the TC-Bob phase only confirms that a message was signed with $K_{TB}$, Bob’s forged signature passes verification. Consequently, Bob can successfully forge Alice’s signature for any message of his choice.

### 3.3. The Main Reasons Why YKLY’2015 Is Insecure

In a signature scheme, TC should be able to judge the validity of the signature and arbitrate the dispute between Alice and Bob. However, by the attacks in Section 3.2, it is not the case in YKLY’2015 [4]. In this section, we discuss the reasons why YKLY’2015 [4] is vulnerable to MITM attacks.

First, according to YKLY’2015 [4], we can know that the process between Alice and TC is used to confirm that the received message *m* was legitimately signed by Alice or not. That is, in Step (AT1), TC cannot confirm that the message in public board (such as the initial message *m* and the forged message $m^{\prime }$) really comes from Alice or not. Otherwise, the subsequent steps in the scheme [4] are unnecessary because the message in public board has been authenticated by TC. In other words, the public board cannot provide authentication so that Eve can announce some messages in public board.

Meanwhile, the required information, including data such as the sender’s quantum signatures and the positions of decoy states, is transmitted over a public channel without any prior encryption. While the protocol specifies that the sender delivers messages to the trusted center (TC), it lacks supplementary safeguards to ensure exclusive reception by the TC. Neither does the protocol guarantee that unauthorized third parties that intercept the data cannot extract valuable information. It is critical to note that although quantum key distribution (QKD) provides information-theoretic unconditional security, this security guarantee only applies to the key generation process, not to the encryption of quantum signature data during transmission.

Therefore, in order to judge which one of *m* and $m^{\prime }$ in public board is Alice’s message, TC has no alternative but to recover the secure key from $U_{S_{AT}}|M\rangle$ or $U_{S_{AT}}|M^{\prime }\rangle$. For the same reason, Alice isn’t able to ensure the following two cases: (1) The receiver of the message that Alice tells to TC is indeed TC; (2) The message received by Alice really comes from TC. This explains why Eve can intercept the signature $U_{S_{AT}}|M\rangle$ during transmission without being detected, as the protocol provides no authentication of the communicating parties.

A critical vulnerability lies in the fact that the verifying unitary operator $U_{V}=2|M\rangle \langle M|-I$ depends entirely on the message *m*, which is publicly announced on the board. Since *m* is known to all parties, anyone, including an eavesdropper, can construct $U_{V}$ independently. This means that the verification procedure provides no authentication: the ability to verify a signature is not restricted to the intended recipient but is available to any adversary who reads the public board. Combined with the unprotected transmission of the signature $U_{S_{AT}}|M\rangle$, this allows Eve to recover the secret key $K_{AT}$ by simply applying $U_{V}$ to the intercepted signature.

TC is only able to confirm that the received message $U_{S_{AT}}|M^{\prime }\rangle$ was signed using the shared key. So, Eve can prove to TC that he is Alice. Since TC can only verify that a message was signed with $K_{AT}$, and Eve has recovered $K_{AT}$, Eve can successfully impersonate Alice to TC. The same weakness also appears in the TC–Bob phase. As a result, TC’s confirmation doesn’t provide message authentication and non-repudiation. In addition, message integrity cannot be guaranteed by the signature key because Eve can obtain the secret key to sign any message. Therefore, none of the three basic conditions is satisfied in YKLY’2015 [4].

## 4. An AQS Scheme on Teleportation-QOTP

In this section, we present an arbitrated quantum signature (AQS) scheme designed to avoid the weaknesses identified in the Grover-based design. We first define the system model and assumptions, then describe the protocol, and finally provide a formal security analysis.

### 4.1. System Model and Assumptions

The proposed scheme is designed for an arbitrated setting where two mutually distrusting parties require a trusted arbitrator to resolve disputes. The following model formalizes the participants, their trust relationships, the communication channels, the adversary capabilities, and the security properties that the protocol must satisfy.

#### 4.1.1. Participants and Main Quantum Primitives

The protocol involves three parties. Alice is the signer, who holds a quantum message |p〉 whose classical description is known to her, and wishes to generate a valid signature. Bob is the receiver and verifier, who authenticates the signed message with the assistance of the arbitrator. Trent is the arbitrator, who is trusted to facilitate signature generation, verification, and dispute resolution.

The quantum message to be signed, denoted by |p〉, is assumed to be a state whose classical description is known to Alice. Consequently, Alice can prepare multiple identical copies of |p〉 without violating the no-cloning theorem. This assumption is adopted throughout the protocol and distinguishes our scheme from signature protocols designed for completely unknown quantum states.

Our protocol employs three main quantum primitives: the strengthened quantum one-time pad for encrypting quantum states (see Section 2 for details), quantum teleportation for transferring quantum states (see Section 2 for details), and the swap test for comparing quantum states during the verification phase, which we now describe in more detail.

The swap test is a well-established probabilistic procedure for comparing unknown quantum states that has been widely adopted in quantum information processing. It takes two states |ψ〉 and |ϕ〉 as input, and applies a controlled-SWAP gate between them, controlled on an auxiliary qubit initialized in |+〉, followed by a Hadamard gate on the auxiliary qubit and a measurement in the computational basis. When the two states are identical, the test always outputs “equal”. When they differ, the test outputs “equal” with probability (1+F)/2 and “not equal” with probability (1−F)/2, where $F={\left|\langle \psi |\phi \rangle \right|}^{2}$ denotes the fidelity between the two states. For orthogonal states (F=0), this reduces to 1/2 each. By repeating the test *m* times on independent copies, the probability that all *m* tests erroneously output “equal” for two different states is ${\left[\left(1+F\right)/2\right]}^{m}$. For orthogonal states (F=0), this reduces to ${\left(1/2\right)}^{m}=2^{-m}$.

In a practical protocol, the participants adopt a fidelity threshold $F_{\max}$, which captures the maximum fidelity that a modification may achieve without producing a semantically meaningful alteration of the message. Throughout this paper, $F_{\max}$ is treated as an application-level threshold, chosen according to the application scenario and the required verification strictness, in the same way that the security parameter λ is chosen according to the required security level. Any meaningful forgery must reduce the fidelity to at most $F_{\max}$, i.e., $F\leq F_{\max}$. The false-acceptance probability after λ repetitions is then bounded by ${\left[\left(1+F_{\max}\right)/2\right]}^{\lambda }$, and the detection probability is at least $1-{\left[\left(1+F_{\max}\right)/2\right]}^{\lambda }$. For instance, in a contract-signing setting, a modification that changes only a negligible portion of the contract does not constitute a meaningful forgery, just as in classical contract law a trivial typographical change does not invalidate the substance of the agreement. If the application demands the strictest verification, one may set $F_{\max}=0$, in which case the bound reduces to $2^{-\lambda }$.

The resource cost of a single swap test is modest: it requires one auxiliary qubit, one controlled-SWAP gate per pair of compared qubits, and one Hadamard gate, plus a single-qubit measurement. For single-qubit states, these operations are constant. For *n*-qubit registers, the number of controlled-SWAP gates scales as O(n), while the auxiliary qubit and measurement overhead remain constant. When the test is repeated m=λ times, the total resource cost scales linearly with λ. In addition, each repetition consumes the two input copies, so λ repetitions require λ independent physical copies of each state being compared. In the proposed protocol, every occurrence of a message copy is therefore understood as a message group consisting of the required number of physical copies. Specifically, the logical copy $|p_{1}\rangle$ is realized as 2λ+1 physical copies, while the logical copies $|p_{2}\rangle$ and $|p_{3}\rangle$ are each realized as λ physical copies. All 2λ+1 physical copies of $|p_{1}\rangle$ are transmitted to Trent through the two-stage teleportation chain via Bob. Among them, λ copies are consumed by Trent in the λ swap tests during the intermediate verification, and the remaining λ+1 copies are teleported back to Bob for the final verification. The λ copies of $|p_{3}\rangle$ serve as the arbitrator’s reference copies, and the λ copies of $|p_{2}\rangle$ serve as the receiver’s reference copies. All these copies are prepared by the signer, which is possible because the signer knows the classical description of the message. In total, Alice prepares 4λ+1 physical copies of the message. Among them, $|p_{2}\rangle$ and $|p_{3}\rangle$ are encrypted with the strengthened QOTP and transmitted directly to Bob and Trent, respectively, consuming λ physical copies each. The remaining 2λ+1 physical copies of $|p_{1}\rangle$ are transmitted through the teleportation chain from Alice to Bob and then to Trent. The first teleportation hop consumes 2λ+1 Bell pairs, the second hop consumes 2λ+1 Bell pairs, and the final hop from Trent back to Bob consumes λ+1 Bell pairs, giving 5λ+3 Bell pairs in total. Accordingly, the QOTP encryption cost and the direct communication cost each scale as O(λn), the teleportation cost scales as O(λ) Bell pairs, and the swap-test circuit cost scales as O(λn) for an *n*-qubit message. Since λ is fixed once the security level and the fidelity threshold are chosen, all these costs are constant multiples of the logical message size, and the asymptotic complexity remains O(n). In the protocol description, the swap test between two logical copies is understood as λ independent swap tests between the corresponding physical copies in their message groups. For brevity, the protocol description omits these physical copy details and refers to each logical message copy directly.

In cryptographic protocols, it is standard practice to anchor all security-critical parameters to a single security parameter, typically denoted by λ, which quantifies the overall security strength of the system. The defining property is that any adversary should succeed with probability at most $2^{-\lambda }$, which is a negligible function in λ. Following this principle, we set the repetition count of the swap test to m=λ. With the fidelity threshold $F_{\max}$, the corresponding false-acceptance bound is ${\left[\left(1+F_{\max}\right)/2\right]}^{\lambda }$, which reduces to $2^{-\lambda }$ when $F_{\max}=0$. The same parameter λ governs the length of pre-shared authentication keys and the number of bits consumed by the quantum one-time pad, so that every component of the protocol operates at a consistent security level. For practical deployment, the value of λ can be chosen according to the required security level.

#### 4.1.2. Trust Model

**Trent is trusted.** The arbitrator follows the protocol honestly and does not collude with either Alice or Bob. Trent is responsible for key distribution, signature verification, and dispute resolution. This assumption reflects the role of trusted institutions such as notaries, certification authorities, and judicial bodies in conventional legal and commercial frameworks. In such settings, the trusted third party is assumed to be impartial and bound by procedural rules, which makes the assumption both natural and well-motivated. We further assume that Trent does not collude with either party individually. If Trent were to collude with Alice, he could help her repudiate a valid signature. If Trent were to collude with Bob, he could help him forge a signature. Both scenarios would render the arbitrated model meaningless and are therefore excluded from our security analysis.

**Alice and Bob are mutually distrusting.** Each party may attempt to cheat the other. Alice may try to repudiate a signature she has generated, while Bob may attempt to forge a signature or alter a signed message. This assumption models realistic adversarial behavior in contractual relationships, where neither party has reason to trust the other’s honesty and each acts in their own interest.

**No collusion between Alice and Bob.** We assume that Alice and Bob do not collude to defraud the arbitrator or to compromise the protocol. This is a standard and well-motivated assumption in arbitrated signature schemes. If Alice and Bob were willing to collude, they would be acting in concert rather than in conflict, and the protocol would no longer be needed. The purpose of an arbitrated signature is to protect each party against a potentially dishonest counterparty. When both parties choose to cooperate, there is simply no dispute for the arbitrator to resolve, and the scenario falls outside the intended scope of the model.

The following example illustrates a realistic setting in which all three assumptions of our trust model naturally hold. Consider two multinational corporations, Alpha Corporation and Beta Limited, that have no prior business relationship and wish to sign a high-value procurement contract remotely. Neither party fully trusts the other: Alpha Corporation worries that Beta Limited may later deny having signed the contract or alter its terms, while Beta Limited worries that Alpha Corporation may forge its signature on a different document. To resolve this mutual distrust, both parties agree to use an internationally recognized third-party platform, such as a global bank or a cross-border digital notary service, to arbitrate the signing process. In our scheme, this platform plays the role of Trent. The signer executes the proposed protocol, and the platform verifies that the quantum signature is consistent with the signer’s identity and the contract content before forwarding the verified message to the receiver. If a dispute arises later, the platform serves as the authoritative witness because it has already verified the authenticity and integrity of the signed contract. This example satisfies all three assumptions of our trust model: (i) the platform is an established institution with legal obligations to remain impartial, so Trent is trusted and does not collude with either party; (ii) Alpha Corporation and Beta Limited are mutually distrusting, as they have no established relationship and each has incentives to protect its own interests; and (iii) the two companies do not collude, because they are contractual counterparts with conflicting interests. This setting is realistic because existing cross-border commerce already relies on centralized intermediaries such as escrow services, correspondent banks, and notarial platforms to mediate transactions and provide certification. Such scenarios are widespread in practice, which demonstrates the broad applicability of the trusted-arbitrator model assumed in this work.

#### 4.1.3. Channel Assumptions

We assume that the classical channels between all parties are authenticated but not necessarily confidential. An adversary may observe the messages on these channels but cannot modify them without detection. The quantum channels are assumed to be fully insecure: an adversary may intercept, measure, or replace quantum states in transit. The security of our protocol does not rely on the confidentiality of the quantum channels, since all transmitted quantum states, with the exception of the λ plaintext copies of $|p_{2}\rangle$ sent from Alice to Bob for Bob’s final verification, are encrypted via the quantum one-time pad before transmission. We also assume that quantum key distribution links are available between each pair of parties to establish the pre-shared keys $K_{AT}$ and $K_{BT}$.

#### 4.1.4. Adversary Model

We consider two classes of adversaries. An external adversary is not a legitimate participant in the protocol and does not possess any pre-shared keys, but has full access to the classical and quantum channels and may eavesdrop on all classical messages, intercept quantum states in transit, inject forged quantum states or classical messages into the channels, capture a transmitted state and perform arbitrary quantum operations on it before forwarding the result to the intended recipient, and record and later retransmit previously observed messages or states. For the analysis of quantum-state confidentiality, the external adversary is assumed to be computationally unbounded but limited by the laws of quantum mechanics. For the analysis of unforgeability and non-repudiation, however, we restrict the adversary to be polynomial-time in the security parameter λ, because those properties rely on classical cryptographic primitives whose security is computational rather than information-theoretic. Internal adversaries consist of a dishonest Alice or a dishonest Bob, either of whom may deviate arbitrarily from the protocol specification. A dishonest Alice may attempt to repudiate a valid signature that she has genuinely generated by tampering with the quantum states she prepares, sending inconsistent classical messages, or exploiting the dispute resolution procedure. A dishonest Bob may attempt to forge a valid signature by manipulating the verification process, altering the quantum states he receives, or presenting modified evidence to Trent during dispute resolution. Both internal adversaries have full knowledge of the protocol specification and access to their own keys and quantum resources, but they do not possess the keys held by the other party or by Trent.

#### 4.1.5. Security Properties and Formal Security Games

Under the above model, the proposed scheme aims to satisfy unforgeability and non-repudiation. We now define these properties through formal security games between a challenger and an adversary.

**Unforgeability.** Unforgeability requires that no adversary can generate a valid signature attributable to Alice without Alice’s participation. We define the following game between a challenger C and an adversary A, where A may be an external adversary or a dishonest Bob. In the setup phase, C generates the pre-shared keys $K_{AT}$ and $K_{BT}$ and gives $K_{BT}$ to A. If A is an external adversary, it receives no keys. In the query phase, A may polynomially many times request Alice to sign messages of its choice. For each query with message $|p_{i}\rangle$, C runs the protocol on behalf of Alice and Trent and returns the resulting transcript to A. In the forgery phase, A outputs a message $|p^{*}\rangle$ and a claimed signature $\sigma^{*}$, where $|p^{*}\rangle$ was never queried in the previous phase. In the verification phase, C runs the verification procedure on behalf of Trent. A wins if Trent outputs “valid” and attributes the signature to Alice. The scheme satisfies unforgeability if, for any polynomial-time adversary A, the probability that A wins this game is a negligible function in λ, where λ is the security parameter. The restriction to polynomial-time adversaries applies to unforgeability because the protocol’s authentication components are computationally secure.

**Non-repudiation.** Non-repudiation requires that if Alice genuinely generates a valid signature, she cannot later deny having done so. We define the following game between a challenger C and a dishonest Alice A. In the setup phase, C generates the pre-shared keys $K_{AT}$ and $K_{BT}$ and gives $K_{AT}$ to A, and also initializes Bob and Trent with their respective keys. In the signature generation phase, A follows the protocol honestly and generates a valid signature σ on a message |p〉 of her choice, with Bob and Trent both following the specification honestly. In the repudiation attempt phase, A outputs a claim that the signature σ is invalid, together with any evidence she wishes to present to the arbitrator. In the dispute resolution phase, C invokes Trent’s dispute resolution procedure using the evidence provided by A and any records held by Bob and Trent. A wins if Trent concludes that the signature is invalid. The scheme satisfies non-repudiation if, for any polynomial-time dishonest Alice A, the probability that A wins this game is a negligible function in λ, where λ is the security parameter. The restriction to polynomial-time adversaries applies to non-repudiation because the dispute-resolution records rely on computationally secure classical authentication.

### 4.2. The Proposed Scheme

To satisfy the security properties defined in Section 4.1, the proposed scheme combines entanglement distribution, quantum teleportation [24], and strengthened quantum one-time pad encryption [16]. The protocol consists of two phases, the initializing phase and the signing-verifying phase. For simplicity, the protocol is described for a single physical copy of the quantum message and a single Bell pair at each teleportation step. The description of the operations on one copy completely determines the operations on all other copies. In actual execution, the required numbers of message copies, Bell pairs, and classical key bits are supplied in sufficient quantities according to the consumption of each step, and these numbers are summarized after the protocol description.

#### 4.2.1. Initializing Phase

In this phase, the secret keys are shared and the Bell states are generated.

(I1) Alice and Bob share their secret keys $K_{AT}\in {\left\{0,1\right\}}^{16},K_{BT}\in {\left\{0,1\right\}}^{24}$ with Trent by QKD protocols [19,20,21] respectively, which are proved to be unconditionally secure [25,26]. $K_{AT}$ is divided into three segments: $K_{AT}^{\prime }$ (bits 1–4), $K_{AT}^{\prime \prime }$ (bits 5–8), and $K_{AT}^{\prime \prime \prime }$ (bits 9–16). Similarly, $K_{BT}$ is divided into four segments: $K_{BT}^{\prime }$ (bits 1–4), $K_{BT}^{\prime \prime }$ (bits 5–12), $K_{BT}^{\prime \prime \prime }$ (bits 13–16), and $K_{BT}^{\left(4\right)}$ (bits 17–24). In practice, QKD can operate continuously in the background to maintain a pool of pre-shared keys. The keys required for each signature are then drawn from this pool, so that the key generation does not delay the signing process. The key-partition pattern specified above, namely the 16-bit $K_{AT}$ and the 24-bit $K_{BT}$, describes the key material consumed by one physical-copy processing path. In actual execution with multiple physical copies, fresh key blocks with the same internal structure are drawn from the QKD key pool for each copy, and no QOTP key block is reused across different encrypted objects.

(I2) Trent generates a sufficient number of Bell states $|\varphi^{+}\rangle =\frac{1}{\sqrt{2}}(|00\rangle +\left|11\rangle \right)$. He sends one particle of a Bell state to Alice and the other to Bob, establishing a shared Bell state $|\varphi^{+}\rangle_{AB}$ for Step (S2). He sends one particle of another Bell state to Bob and keeps the other, establishing a shared Bell state for Step (S4). He sends one particle of another Bell state to Bob and keeps the other, establishing a shared Bell state for Step (S6). The exact numbers of Bell states used in these steps are given in the resource summary after the protocol description. We assume that these entanglement distribution steps can be realized via standard quantum communication techniques, using quantum repeaters [27] if the parties are far away from each other [25]. The security of the distributed entanglement can be ensured by entanglement purification and quantum state authentication [28].

#### 4.2.2. Signing-Verifying Phase

In this phase, a quantum message |p〉 is signed by Alice and verified by Trent and Bob.

The first three steps are performed by Alice.

(S1) Alice prepares the quantum message in three groups: $|p_{1}\rangle$, $|p_{2}\rangle$, and $|p_{3}\rangle$. Each group consists of the required number of physical copies as specified in the resource summary. As discussed in Section 4.1, this is possible because Alice knows the classical description of |p〉. The notation $|p_{1}\rangle$, $|p_{2}\rangle$, $|p_{3}\rangle$ refers to these groups throughout the protocol.

(S2) Alice combines one physical copy of $|p_{1}\rangle$ with the Bell state $|\varphi^{+}\rangle_{AB}$ shared with Bob to obtain a three-particle entangled state, and performs a Bell measurement on the message particle and the Bell state particle in her hand. Then, the corresponding Bell state particle in Bob’s hand becomes $U\left(X_{1}\right)|p_{1}\rangle$. Here, $X_{1}$ is determined by the measurement outcome in Alice’s hand, and is determined randomly by the measurement outcome from the set {00,01,10,11}. Note that Alice can obtain $X_{1}$. In actual execution, this step is repeated for each physical copy of $|p_{1}\rangle$, and the corresponding measurement outcomes are denoted $X_{1}^{\left(i\right)}$.

(S3) Alice sends $|p_{2}\rangle$ and $E_{K_{AT}^{\prime }}|p_{3}\rangle$ to Bob and Trent, respectively. After encrypting the second particle of the Bell state in her hand by $E_{K_{AT}^{\prime \prime }}$ and $|X_{1}\rangle$ by $K_{AT}^{\prime \prime \prime }$, she sends the particle pair (its state is $\left[I⊗E_{K_{AT}^{\prime \prime }}U\left(X_{1}\right)\right]|\varphi^{+}\rangle$) and $E_{K_{AT}^{\prime \prime \prime }}|X_{1}\rangle$ to Trent. In actual execution, this step is repeated for each physical copy of $|p_{1}\rangle$ and the corresponding $X_{1}^{\left(i\right)}$ value.

Next step is performed by Bob.

(S4) Bob combines the received copy $U\left(X_{1}\right)|p_{1}\rangle$ with the Bell state shared with Trent (established in Step (I2) for this purpose) to obtain a three-particle entangled state, and performs a Bell measurement on the message particle and the Bell state particle in his hand. Then, the corresponding Bell state particle in Trent’s hand becomes $U\left(X_{2}\right)U\left(X_{1}\right)|p_{1}\rangle$. Note that Bob can obtain $X_{2}$. After encrypting the second particle of the Bell state in his hand by $E_{K_{BT}^{\prime }}$ and $|X_{2}\rangle$ by $K_{BT}^{\prime \prime }$, Bob sends the particle pair (its state is $\left[I⊗E_{K_{BT}^{\prime }}U\left(X_{2}\right)\right]|\varphi^{+}\rangle$) and $E_{K_{BT}^{\prime \prime }}|X_{2}\rangle$ to Trent. In actual execution, this step is repeated for each received copy, and the corresponding measurement outcomes are denoted $X_{2}^{\left(i\right)}$.

The next two steps are performed by Trent.

(S5) Trent decrypts the particle pair from Alice using $K_{AT}^{\prime \prime }$ and measures it to obtain $X_{1}$. He then decrypts $E_{K_{AT}^{\prime \prime \prime }}|X_{1}\rangle$ using $K_{AT}^{\prime \prime \prime }$ to obtain $|X_{1}\rangle$, and compares the measured $X_{1}$ with the decrypted value. If they differ, the protocol aborts. Similarly, Trent decrypts the particle pair from Bob using $K_{BT}^{\prime }$ to obtain $X_{2}$, and compares it with the decrypted $E_{K_{BT}^{\prime \prime }}|X_{2}\rangle$. If they differ, the protocol aborts. Trent then applies $U(X_{1})$ and $U(X_{2})$ to recover $|p_{1}\rangle$ from $U\left(X_{2}\right)U\left(X_{1}\right)|p_{1}\rangle$. In actual execution, this step is repeated for each copy, yielding the recovered copies $|p_{1}^{\left(i\right)}\rangle$. Trent randomly selects the required number of recovered copies and compares them with $|p_{3}\rangle$ using the swap test described in Section 4.1. If any test fails, the protocol aborts. Otherwise, Trent retains the remaining copies of $|p_{1}\rangle$ and proceeds to Step (S6).

(S6) Trent combines one of the remaining copies of $|p_{1}\rangle$ with the Bell state shared with Bob (established in Step (I2) for this purpose) to obtain a three-particle entangled state, and performs a Bell measurement on the message particle and the Bell state particle in his hand. Then, the corresponding Bell state particle in Bob’s hand becomes $U\left(X_{3}\right)|p_{1}\rangle$. After encrypting the second particle of the Bell state in his hand by $E_{K_{BT}^{\prime \prime \prime }}$ and $|X_{3}\rangle$ by $K_{BT}^{\left(4\right)}$, Trent sends the particle pair (its state is $\left[I⊗E_{K_{BT}^{\prime \prime \prime }}U\left(X_{3}\right)\right]|\varphi^{+}\rangle$) and $E_{K_{BT}^{\left(4\right)}}|X_{3}\rangle$ to Bob. In actual execution, this step is repeated for each remaining copy, and the corresponding measurement outcomes are denoted $X_{3}^{\left(i\right)}$.

**Figure 3:**
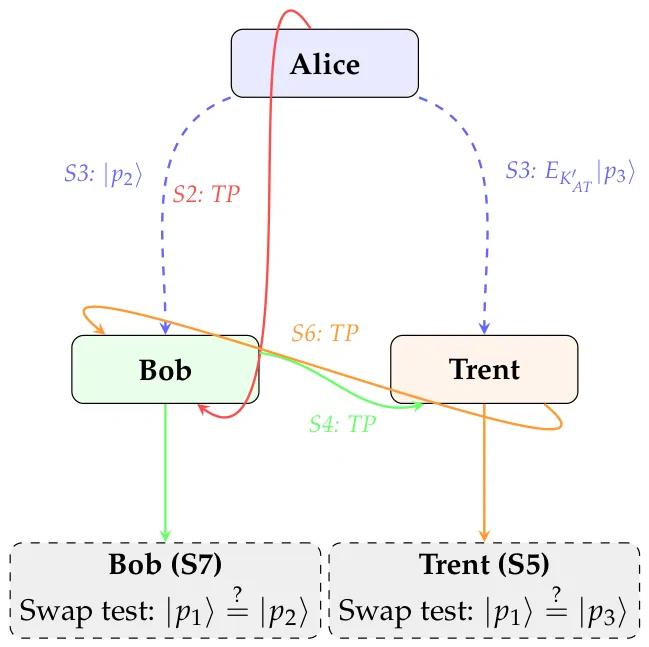
Transmission overview of the signing-verifying phase. Dashed straight arrows denote the direct distribution of the three message groups in S3. Curved arrows denote the teleportation hops S2, S4, and S6, which relay $|p_{1}\rangle$ along the path Alice → Bob → Trent → Bob.

**Remark on the message flow.** The signing-verifying phase realizes a two-stage teleportation chain that routes the message copy $|p_{1}\rangle$ along the closed path Alice → Bob → Trent → Bob. In the first stage, Alice teleports the state $U\left(X_{1}\right)|p_{1}\rangle$ to Bob (S2), and Bob teleports the received state to Trent (S4), so that after decrypting $X_{1}$ and $X_{2}$, Trent can recover $|p_{1}\rangle$ without Alice and Trent sharing a direct teleportation channel. After Trent verifies the consistency between the teleported copy and the encrypted copy $|p_{3}\rangle$ (S5), the second stage begins: Trent teleports the verified message back to Bob (S6), who performs a final consistency check against his own copy $|p_{2}\rangle$ (S7). Each teleportation hop consumes one Bell pair and produces a two-bit classical outcome. The outcomes of the first two hops are denoted $X_{1}$ and $X_{2}$, and the outcomes of the final hop are denoted $X_{3}$. In actual execution, there are multiple such outcomes corresponding to the multiple physical copies, but the operation on each copy is identical to the single-copy description given here. All such outcomes are transmitted in encrypted form ($X_{1},X_{2}$ to Trent, and $X_{3}$ to Bob), and every quantum transmission is protected by the strengthened quantum one-time pad. Consequently, Bob cannot forge the signature without being detected, because the teleported state he receives is encrypted by the unknown operation $U(X_{1})$, and any tampering would be detected by Trent’s swap test, and Trent cannot tamper with the message without invalidating Bob’s final check. The overall transmission structure is illustrated in Figure 3.

The final step is performed by Bob to verify and accept the message.

(S7) Bob decrypts the particle pair from Trent using $K_{BT}^{\prime \prime \prime }$ and measures it to obtain $X_{3}$. He then compares this value with the decrypted $|X_{3}\rangle$ value obtained by decrypting $E_{K_{BT}^{\left(4\right)}}|X_{3}\rangle$ using $K_{BT}^{\left(4\right)}$. If they differ, the protocol aborts. Otherwise, Bob applies $U(X_{3})$ to recover one copy of $|p_{1}\rangle$. In actual execution, this step is repeated for each received copy, yielding the recovered copies $|p_{1}^{\left(i\right)}\rangle$. Bob randomly selects the required number of recovered copies and compares them with $|p_{2}\rangle$ using the swap test described in Section 4.1. If all tests match, Bob accepts the message and retains the remaining one copy of $|p_{1}\rangle$ as the signed quantum message. Otherwise, the protocol aborts.

**Resource summary.** In actual execution, the numbers of physical copies and Bell pairs are as follows. Alice prepares 2λ+1 physical copies of $|p_{1}\rangle$, λ physical copies of $|p_{2}\rangle$, and λ physical copies of $|p_{3}\rangle$. Step (S2) consumes 2λ+1 Bell pairs shared between Alice and Bob. Step (S4) consumes 2λ+1 Bell pairs shared between Bob and Trent. Step (S5) consumes λ copies of $|p_{1}\rangle$ and λ copies of $|p_{3}\rangle$ in the swap test. Step (S6) consumes λ+1 Bell pairs shared between Trent and Bob. Step (S7) consumes λ copies of $|p_{1}\rangle$ and λ copies of $|p_{2}\rangle$ in the swap test. After the protocol, Bob retains exactly one physical copy of $|p_{1}\rangle$ as the signed quantum message.

**Figure 4:**
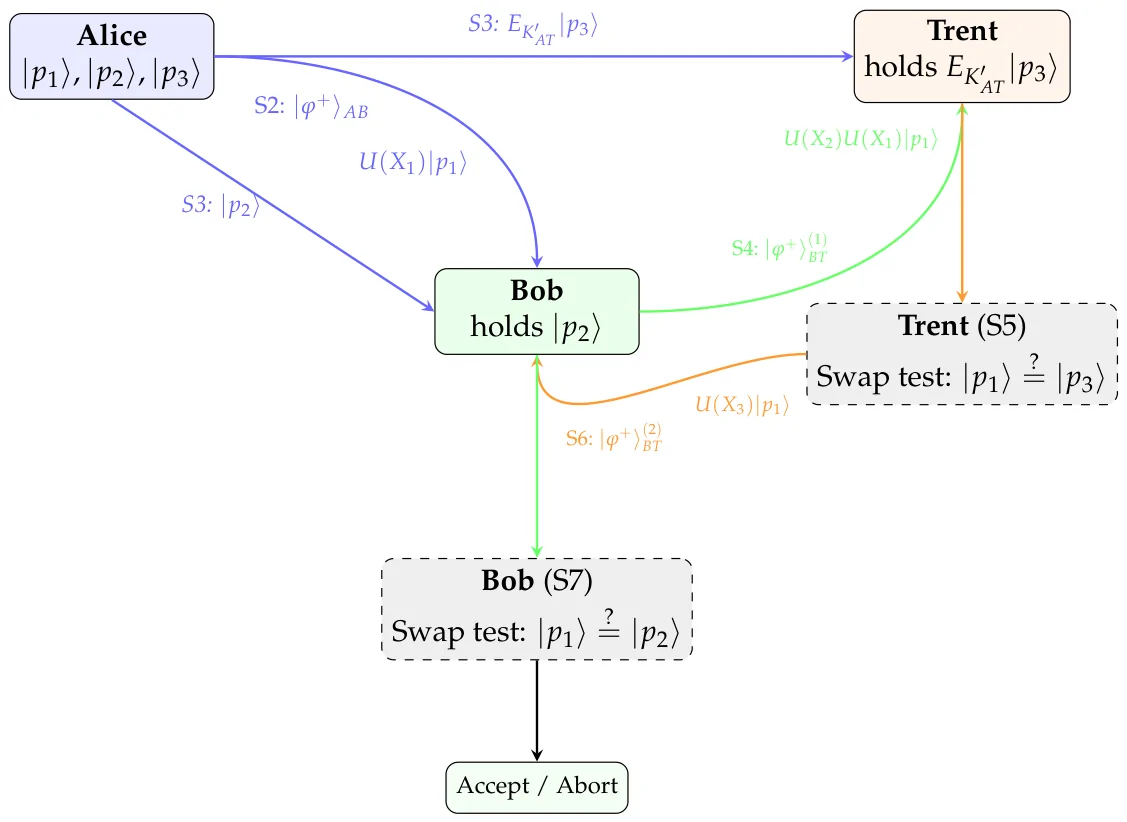
Flow of the three message groups and Bell state consumption in the proposed scheme.

In summary, Alice transmits the quantum message to Trent via two rounds of teleportation through Bob. Trent verifies the message and teleports it back to Bob, who performs a final verification before acceptance. Throughout the protocol, all quantum states are protected by the strengthened quantum one-time pad, and all state comparisons are performed using the swap test. For clarity, the protocol execution flow is illustrated in Figure 4.

### 4.3. Security Analysis of the Proposed Scheme

We now analyze the security of the proposed scheme with respect to the formal definitions of non-repudiation and unforgeability established in Section 4.1. The analysis considers the three classes of adversaries defined in our model: a dishonest Alice, a dishonest Bob, and external adversaries. We then discuss the resilience of the protocol against other standard attack vectors.

#### 4.3.1. Non-Repudiation

We show that the proposed scheme satisfies the non-repudiation game defined in Section 4.1. Let A be a dishonest Alice who attempts to repudiate a valid signature.

In the signature generation phase of the game, Alice honestly produces a valid signature on a message |p〉 of her choice. The protocol executes normally with Bob and Trent following the specification. In the repudiation attempt phase, Alice outputs a claim that the signature is invalid and presents evidence to the arbitrator. For Alice to win, Trent must conclude that the signature is invalid despite Alice having genuinely generated it.

The analysis below considers a stronger adversary than the one defined in the non-repudiation game. Alice is allowed to deviate already during the signature generation phase, not only during the repudiation attempt phase. If the protocol resists this stronger adversary, it certainly resists the standard one. We now show that any attempt by Alice to repudiate is detected by the protocol’s verification mechanisms. Suppose Alice mounts the strongest possible attack by deviating from the protocol in every step where she has control. Specifically, in Step (S3), Alice sends $|p_{2}^{\prime }\rangle$ to Bob, $E_{K_{AT}^{\prime }}|p_{3}^{\prime }\rangle$ to Trent, and a particle pair encoding $X_{1}^{\prime }$ together with $E_{K_{AT}^{\prime \prime \prime }}|X_{1}^{\prime }\rangle$ to Trent. These may differ arbitrarily from the honest values.

For the protocol to proceed beyond Step (S5), Trent must successfully verify both $X_{1}$ and $X_{2}$, and the swap test between the recovered $|p_{1}\rangle$ and $|p_{3}\rangle$ must pass. The verification of $X_{1}$ requires that the value measured from Alice’s particle pair matches the decrypted $|X_{1}^{\prime }\rangle$. Since Alice controls both the particle pair and the encrypted $X_{1}^{\prime }$, she can ensure this check passes by sending consistent values. However, the swap test imposes a stronger constraint. Trent recovers the teleported state and compares it with $|p_{3}^{\prime }\rangle$. Let $|p_{1}^{\mathrm{rec}}\rangle$ denote the state recovered by Trent. For the swap test to pass with high probability, we must have $|p_{1}^{\mathrm{rec}}\rangle ≅|p_{3}^{\prime }\rangle$. Given the teleportation chain and assuming Bob behaves honestly, Trent first verifies that the $X_{1}$ value measured from Alice’s particle pair matches the decrypted value. If Alice sends inconsistent values, the protocol aborts before the swap test. If Alice sends consistent values, Trent’s recovery yields $|p_{1}^{\mathrm{rec}}\rangle =|p_{1}\rangle$. The swap test then compares $|p_{1}\rangle$ with $|p_{3}^{\prime }\rangle$, which imposes the condition $|p_{1}\rangle ≅|p_{3}^{\prime }\rangle$.

Now suppose the protocol continues to Step (S7). Bob receives the state teleported by Trent, applies $U(X_{3})$ to recover $|p_{1}\rangle$, and performs a swap test with $|p_{2}^{\prime }\rangle$. For Bob to accept, the swap test must pass, which implies $|p_{2}^{\prime }\rangle ≅|p_{1}\rangle ≅|p_{3}^{\prime }\rangle$.

This is the crucial observation: if Bob accepts the signature, then the message $|p_{2}^{\prime }\rangle$ held by Bob, the message $|p_{3}^{\prime }\rangle$ that Trent verified in Step (S5), and Alice’s original $|p_{1}\rangle$ are provably connected through the relation $|p_{2}^{\prime }\rangle ≅|p_{3}^{\prime }\rangle ≅|p_{1}\rangle$. Trent, who possesses the authenticated classical record of the encrypted transmission and the records of $X_{1}$ and $X_{2}$, can verify this chain of derivation. Since $|p_{2}^{\prime }\rangle$ is the message that Bob received from Alice, and Trent can confirm that it originated from Alice’s original $|p_{1}\rangle$, Alice cannot later claim that she did not sign this message.

Even in the extreme case where Alice sends $|p_{2}^{\prime }\rangle$ that differs from $|p_{1}\rangle$, the consistency enforced by the protocol ensures that $|p_{2}^{\prime }\rangle ≅|p_{3}^{\prime }\rangle$, meaning that whatever message Bob received is exactly what Trent verified. Alice cannot later claim that Bob received a different message than what she sent, because Trent’s records provide an unforgeable audit trail.

We now quantify Alice’s success probability. For Alice to win the non-repudiation game, Trent must conclude that the signature is invalid despite the protocol having produced an accepted signature. From the chain of derivation above, Bob’s acceptance implies $|p_{2}^{\prime }\rangle ≅|p_{3}^{\prime }\rangle ≅|p_{1}\rangle$. Trent holds authenticated classical records of Alice’s encrypted transmission $E_{K_{AT}^{\prime }}|p_{3}\rangle$ and of the teleportation outcome $X_{1}$. Alice’s only freedom is to choose $|p_{2}^{\prime }\rangle$ and $|p_{3}^{\prime }\rangle$ at the time of signing. If these choices are inconsistent with the teleportation outcome, the swap test in Step (S5) rejects with probability at least $1-{\left[\left(1+F_{\max}\right)/2\right]}^{\lambda }$. If they are consistent, the accepted message is traceable to Alice through Trent’s records. Therefore, Alice’s repudiation succeeds only if the swap test erroneously accepts an inconsistent state, which occurs with probability at most ${\left[\left(1+F_{\max}\right)/2\right]}^{\lambda }$, a negligible function in λ. Hence, Alice cannot win the non-repudiation game with non-negligible probability.

#### 4.3.2. Unforgeability

We show that the proposed scheme satisfies the unforgeability game defined in Section 4.1. Let A be a polynomial-time adversary, which may be either a dishonest Bob or an external adversary. We consider each case separately.

**Dishonest Bob.** A dishonest Bob possesses the key $K_{BT}$ and participates in the protocol as the designated receiver. His goal is to forge a valid signature—that is, to produce a message-signature pair that passes Trent’s verification and is attributed to Alice, without Alice having signed that message.

Bob’s capabilities in the protocol are as follows. After Step (S3), Bob holds $|p_{2}\rangle$ (the plaintext copy sent by Alice) and $U\left(X_{1}\right)|p_{1}\rangle$ (the teleported state). In Step (S4), Bob performs a Bell measurement and learns $X_{2}$. After Step (S7), Bob additionally holds $U\left(X_{3}\right)|p_{1}\rangle$ (the state teleported back by Trent) and learns $X_{3}$.

To forge a signature, Bob must produce a message $|p^{*}\rangle$ and evidence that causes Trent to attribute it to Alice. There are two stages at which Bob might attempt to cheat.

In the first stage (during protocol execution), Bob could modify $|p_{2}\rangle$ or apply a non-identity operation *G* on $U\left(X_{1}\right)|p_{1}\rangle$ before teleporting it to Trent in Step (S4). If Bob applies *G*, the state reaching Trent in Step (S5) becomes $U\left(X_{2}\right)GU\left(X_{1}\right)|p_{1}\rangle$. For the protocol to proceed, Trent must successfully verify $X_{1}$ and $X_{2}$. The verification of $X_{1}$ depends on Alice’s encryption with $K_{AT}$. By the information-theoretic security of the QOTP, Bob cannot determine $X_{1}$ from the ciphertext without $K_{AT}$. If Bob sends an inconsistent $X_{2}$, the decryption check in Step (S5) fails and the protocol aborts. If Bob passes the $X_{2}$ check but modified the quantum state, Trent applies $U(X_{1})$ and $U(X_{2})$ to the received state. Since Pauli operators either commute or anti-commute, the recovered state becomes $U\left(X_{1}\right)GU\left(X_{1}\right)|p_{1}\rangle$ (up to a global phase). Trent then compares it with $|p_{3}\rangle$ via the swap test. Let $F_{G}=|\langle p_{1}|U\left(X_{1}\right)GU\left(X_{1}\right)|p_{1}{\rangle |}^{2}$ denote the fidelity between the recovered state and the reference state $|p_{3}\rangle$. The swap test detects the mismatch with probability $1-{\left[\left(1+F_{G}\right)/2\right]}^{\lambda }$. When G=I, we have $F_{G}=1$ and the test always accepts. When G≠I, the detection probability depends on $F_{G}$; in the worst case for the protocol, *G* may be chosen such that $F_{G}\approx 1$ (e.g., a near-identity operation that preserves the message up to a small perturbation), yielding a low detection probability. We now reduce Bob’s forgery success to the security of the underlying primitives. Bob holds the plaintext copy $|p_{2}\rangle =|p_{1}\rangle$, so he can in principle produce a state with fidelity $F_{G}=1$ to the reference. However, such a state is exactly the original message signed by Alice, and presenting it as a forgery yields no advantage: it is the message Alice already signed. To forge a different message, Bob must produce a state $|p^{*}\rangle \neq |p_{1}\rangle$, whose fidelity to the reference satisfies $F_{G}<1$. The swap test then detects the mismatch with probability $1-{\left[\left(1+F_{G}\right)/2\right]}^{\lambda }$. Bob cannot make $F_{G}$ arbitrarily close to 1 while keeping $|p^{*}\rangle$ semantically different from $|p_{1}\rangle$: under the fidelity threshold $F_{\max}$, any semantically meaningful forgery satisfies $F_{G}\leq F_{\max}$, so the detection probability is at least $1-{\left[\left(1+F_{\max}\right)/2\right]}^{\lambda }$. Therefore, Bob’s forgery success probability is at most ${\left[\left(1+F_{\max}\right)/2\right]}^{\lambda }$, which is negligible in λ.

In the second stage (after protocol completion), Bob possesses $|p_{2}\rangle$ and $U\left(X_{3}\right)|p_{1}\rangle$. After applying $U(X_{3})$, Bob recovers $|p_{1}\rangle$ and compares it with $|p_{2}\rangle$. At this point, Bob already holds the valid message. To forge a different message, Bob would need to produce a new $|p^{*}\rangle$ and corresponding evidence that Trent accepts as originating from Alice. However, Trent retains the authenticated classical transcript of the protocol execution, including the record of Alice’s encrypted transmission, the classical parameters $X_{1}$ and $X_{2}$, and the swap-test outcomes. Any forgery attempt by Bob would require him to produce evidence consistent with this authenticated transcript. Since the transcript is authenticated under keys that Bob does not possess, Bob cannot alter it without detection.

Bob’s knowledge of $X_{2}$ and $X_{3}$ does not assist in forgery, because these values are random Bell measurement outcomes that reveal no information about the signed message. They are independent of the message content and are used solely for reconstructing the teleported state.

Therefore, a dishonest Bob cannot win the unforgeability game with non-negligible probability.

**External adversary.** An external adversary does not possess any of the pre-shared keys $K_{AT}$ or $K_{BT}$. With the exception of the plaintext copy $|p_{2}\rangle$ sent to Bob in Step (S3), all quantum states transmitted during the protocol are encrypted via the strengthened quantum one-time pad, which provides information-theoretic security when the encryption keys are uniformly random and used only once. Specifically, in Step (S3), Alice encrypts $|p_{3}\rangle$ with $K_{AT}^{\prime }$ and the Bell state particle pair with $K_{AT}^{\prime \prime }$. In Step (S4), Bob encrypts his particle pair with $K_{BT}^{\prime }$. In Step (S6), Trent encrypts his particle pair with $K_{BT}^{\prime \prime \prime }$. Without access to these keys, the adversary cannot decrypt the ciphertext to recover the underlying quantum states, nor can it extract meaningful information via the attacks discussed in Section 4.3.3. Consequently, the adversary cannot extract the plaintext from the ciphertext. Furthermore, any active interference by the adversary, such as intercepting and replacing a quantum state in transit, introduces discrepancies that are detected by the subsequent consistency checks, not by the QOTP encryption itself. These discrepancies manifest in two ways. First, the decrypted values $X_{1}$, $X_{2}$, and $X_{3}$ obtained by Trent and Bob will not match the encrypted values sent by the legitimate parties, causing the protocol to abort in Steps (S5) and (S7). Second, even if the adversary somehow managed to preserve the classical parameters, the swap test comparisons in Steps (S5) and (S7) would detect the mismatch between the recovered $|p_{1}\rangle$ and the stored copies $|p_{3}\rangle$ and $|p_{2}\rangle$, respectively. The swap test fails to detect a difference with probability ${\left[\left(1+F\right)/2\right]}^{\lambda }\leq {\left[\left(1+F_{\max}\right)/2\right]}^{\lambda }$, where *F* is the fidelity between the recovered state and the reference state, and $F_{\max}$ is the fidelity threshold defined in Section 4.1. For orthogonal states (F=0), this reduces to $2^{-\lambda }$, which is negligible. We now summarize the external adversary’s success probability. To win the unforgeability game, the adversary must produce a message-signature pair that passes Trent’s verification. This requires either extracting the plaintext from a QOTP-encrypted state without the key, which succeeds with a negligible probability by the information-theoretic security of the QOTP, or modifying a transmitted state in a way that survives the swap test, which succeeds with probability at most ${\left[\left(1+F_{\max}\right)/2\right]}^{\lambda }$. Therefore, the adversary’s success probability is at most ${\left[\left(1+F_{\max}\right)/2\right]}^{\lambda }$, a negligible function in λ.

#### 4.3.3. Security Against Other Attacks

We now discuss the resilience of the proposed scheme against several additional types of attacks that are relevant to quantum signature protocols.

**Replay attacks.** A replay attack against the proposed scheme would consist of recording the quantum states and classical messages transmitted during a legitimate execution and later retransmitting them in a new session. Two protocol features prevent this. First, every execution uses freshly generated QKD keys $K_{AT}$ and $K_{BT}$. The replayed states, encrypted under an old key, would not decrypt correctly under the current key held by Trent or Bob, causing the verification in Steps (S5) or (S7) to fail. Second, the Bell measurement outcomes $X_{1}$, $X_{2}$, and $X_{3}$ are independently random in each execution. Even if a replayed quantum state were somehow accepted, the accompanying classical $X_{i}$ values from the previous session would not match the values independently measured by Trent and Bob in the current session, and the comparisons in Steps (S5) and (S7) would abort the protocol. Replay attacks are therefore prevented by fresh per-execution QKD keys and independently random Bell outcomes.

**Intercept-and-resend attacks.** In the proposed scheme, an adversary who intercepts a quantum transmission, for instance the particle pair sent by Alice to Trent in Step (S3), faces two obstacles. First, the state is encrypted via the strengthened QOTP using $K_{AT}^{\prime \prime }$, which the adversary does not possess. The QOTP renders the ciphertext statistically indistinguishable from a maximally mixed state, so any measurement yields no information about the underlying plaintext. Second, if the adversary replaces the intercepted state with an arbitrary state and forwards it to Trent, the decryption in Step (S5) will produce a value that does not match the separately transmitted encrypted record $E_{K_{AT}^{\prime \prime \prime }}|X_{1}\rangle$, causing the protocol to abort. The same argument applies to the particle pairs transmitted in Steps (S4) and (S6). If the adversary instead targets the plaintext copy $|p_{2}\rangle$ or the encrypted copy of $|p_{3}\rangle$, any modification will be detected by the swap test comparisons in Steps (S5) and (S7) with probability at least $1-{\left[\left(1+F_{\max}\right)/2\right]}^{\lambda }$. We note that QOTP provides confidentiality and plaintext hiding; integrity and authentication are enforced by these additional protocol checks. The adversary therefore gains no advantage from intercept-and-resend attacks.

**Man-in-the-middle attacks.** A man-in-the-middle adversary must simultaneously impersonate one party to another on both directions of a communication channel. In the proposed scheme, this requires overcoming three independent defenses. First, the classical channels are authenticated, so any modification of the encrypted $X_{i}$ values or other classical messages is detected. Second, all quantum states are QOTP-encrypted; without the corresponding key, the adversary can neither extract the plaintext nor re-encrypt a modified state to pass verification. Third, even if the adversary successfully impersonates one party on one leg, the cross-verification performed by Trent in Step (S5) compares the independently received $X_{1}$ and $X_{2}$ values from Alice and Bob. A discrepancy introduced on any single channel, for example by replacing the particle pair and encrypted $X_{1}$ sent by Alice to Trent in Step (S3), will be detected when Trent decrypts and finds mismatching values. The adversary cannot replace $X_{1}$ without being detected, because the classical authentication check would fail. Symmetric arguments apply to the other communication legs. Under the assumptions of authenticated classical channels and confidential pre-shared keys, man-in-the-middle attacks are effectively prevented.

**Entanglement attacks.** An adversary may attempt to entangle an ancilla with a transmitted quantum state and later measure the ancilla to extract information. In the proposed scheme, all quantum states transmitted over the insecure channels are QOTP-encrypted. The ciphertext appears maximally mixed to any party without the decryption key. If the adversary entangles an ancilla with the ciphertext, the reduced state of the ancilla remains independent of the plaintext. Formally, for a QOTP-encrypted state $\frac{1}{2^{2n}}\sum_{a,b}X^{a}Z^{b}\rho Z^{b}X^{a}⊗\left|a,b\rangle \langle a,b\right|$, tracing out the ciphertext leaves the ancilla uncorrelated with the original message ρ. The adversary therefore gains no information, and this protection holds against computationally unbounded adversaries due to the information-theoretic security of the QOTP.

**Collective and coherent attacks.** In collective attacks, the adversary processes each quantum signal individually but stores ancillae for joint measurement across multiple protocol runs. In coherent attacks, all signals may be processed jointly. The proposed scheme resists these attacks in the sense of preserving plaintext confidentiality across executions. Each execution uses independent, freshly generated QKD keys, so the encrypted states from different runs are statistically independent. Joint measurement across multiple ciphertexts yields no more information than individual analysis. Within a single execution, the security follows from the standard QOTP confidentiality guarantee, which holds against unbounded adversaries. The keys themselves are generated by QKD and are information-theoretically secure.

As above, we have shown that the proposed scheme satisfies non-repudiation and unforgeability under the formal adversarial model defined in Section 4.1. The analysis considers dishonest internal parties and external adversaries separately, and shows that neither class can succeed with non-negligible probability. Together, QOTP encryption, authenticated classical channels, cross-verification by Trent, and per-execution fresh keys protect the protocol against replay, intercept-and-resend, man-in-the-middle, entanglement, and collective or coherent attacks.

## 5. Resource Overhead and Simulation

This section first analyzes resource overheads of the proposed scheme and YKLY’2015, and then simulates the attacks on the proposed scheme.

### 5.1. Resource Overhead Analysis of the Proposed Scheme and YKLY’2015

To assess the practical cost of the security guarantees established in Section 4.3, we now analyze the resource consumption of the proposed scheme. For a single quantum message, all core operations of the proposed scheme maintain constant-level computational complexity. In this subsection, we divide the protocol execution into the initialization stage (I1–I2) and the signal interaction stage (S1–S7) to conduct a comprehensive resource overhead analysis, covering key consumption, qubit overhead, quantum communication overhead, classical communication overhead, and computational complexity of each step.

Step (I1): This step completes QKD key generation and distribution, with a total key consumption of O(λ) bits per protocol execution. The key $K_{AT}$ (16 bits) is divided into three segments: $K_{AT}^{\prime }$ (4 bits) for encrypting $|p_{3}\rangle$, $K_{AT}^{\prime \prime }$ (4 bits) for encrypting the Bell state particle pair, and $K_{AT}^{\prime \prime \prime }$ (8 bits) for encrypting $X_{1}$. The key $K_{BT}$ (24 bits) is divided into four segments: $K_{BT}^{\prime }$ (4 bits) for encrypting the Bell state particle pair, $K_{BT}^{\prime \prime }$ (8 bits) for encrypting $X_{2}$, $K_{BT}^{\prime \prime \prime }$ (4 bits) for encrypting the Bell state particle pair in the verification phase, and $K_{BT}^{\left(4\right)}$ (8 bits) for encrypting $X_{3}$. Each segment is used exactly once and is never reused. All keys are freshly generated via QKD for each protocol execution. The key generation involves only fixed quantum operations per key bit, with an overall computational complexity of O(λ).

Step (I2): No additional secret keys are consumed in this step. 5λ+3 Bell states are prepared and serve as persistent entanglement resources, occupying 2(5λ+3)=10λ+6 static qubits. Among them, 2λ+1 Bell states are shared between Alice and Bob for Step (S2), 2λ+1 Bell states are shared between Bob and Trent for Step (S4), and λ+1 Bell states are shared between Trent and Bob for Step (S6). A total of 7λ+4 qubits are transmitted from Trent to Alice and Bob during entanglement distribution. For the 2λ+1 Alice–Bob Bell pairs, both particles leave Trent, giving 2(2λ+1)=4λ+2 transmitted qubits. For the 2λ+1 Bob–Trent Bell pairs used in Step (S4), one particle is sent to Bob while the other is retained by Trent, giving 2λ+1 transmitted qubits. For the λ+1 Bob–Trent Bell pairs used in Step (S6), one particle is sent to Bob while the other is retained by Trent, giving λ+1 transmitted qubits. These Bell states must be preserved until their respective teleportation steps. Let $t_{\mathrm{comm}}$ denote the one-way classical communication latency between any two parties, and let $t_{\mathrm{op}}$ denote the time for local quantum operations and measurements. Under a fully serial schedule for all physical-copy teleportations, the total protocol execution time, which determines the required storage duration, is $t_{\mathrm{total}}=\left(5\lambda +3\right)t_{\mathrm{comm}}+O\left(t_{\mathrm{op}}\right)$. This latency expression corresponds to serial processing of the physical copies; parallel or pipelined execution can reduce the number of sequential communication intervals. For metropolitan-scale distances on the order of tens of kilometers, the serial estimate places $t_{\mathrm{total}}$ on the order of milliseconds when $t_{\mathrm{comm}}$ is on the order of tens of microseconds in optical fiber. This is well within the coherence times demonstrated by current quantum memory technologies. For longer-distance applications, the required storage time increases accordingly, and practical deployment would require continued progress in coherence-preserving technologies.

Step (S1): No key resources are consumed. 4λ+1 dynamic qubits are occupied for quantum message storage, realized as 2λ+1 copies of $|p_{1}\rangle$, λ copies of $|p_{2}\rangle$, and λ copies of $|p_{3}\rangle$. There is no quantum or classical communication transmission overhead. The operation includes single-qubit state preparation for all physical copies, with a computational complexity of O(λ).

Step (S2): In actual execution, this step is repeated for 2λ+1 copies of $|p_{1}\rangle$, consuming 2λ+1 Bell pairs shared between Alice and Bob, and performing 2λ+1 Bell measurements. No classical bits are transmitted in this step. The computational complexity is O(λ).

Step (S3): Partial segments of $K_{AT}$ are consumed, including 4-bit $K_{AT}^{\prime }$, 4-bit $K_{AT}^{\prime \prime }$, and 8-bit $K_{AT}^{\prime \prime \prime }$. No new persistent qubits are added. Alice sends λ copies of $|p_{2}\rangle$ to Bob and λ copies of $E_{K_{AT}^{\prime }}|p_{3}\rangle$ to Trent. In addition, for each of the 2λ+1 copies of $|p_{1}\rangle$, Alice sends one encrypted particle pair, consisting of two qubits, to Trent, giving 2(2λ+1)=4λ+2 additional qubits. The total quantum communication in this step is therefore λ+λ+4λ+2+4λ+2=10λ+4 qubits.

Step (S4): Partial segments of $K_{BT}$ are consumed, including $K_{BT}^{\prime }$ and $K_{BT}^{\prime \prime }$. In actual execution, this step is repeated for 2λ+1 received copies. A total of 2(2λ+1)=4λ+2 encrypted Bell particle qubits are transmitted, accompanied by 2λ+1 encrypted parameters $E_{K_{BT}^{\prime \prime }}|X_{2}^{\left(i\right)}\rangle$, each transmitted as a two-qubit quantum state, adding 2(2λ+1)=4λ+2 qubits. The total quantum communication in this step is therefore 8λ+4 qubits. The core operations are Bell measurement and QOTP encryption, and the computational complexity is O(λ).

Step (S5): No new key segments and persistent qubits are consumed. There is no quantum and classical communication overhead. In actual execution, Trent recovers 2λ+1 copies of $|p_{1}\rangle$. Among them, λ copies are used in the swap test with $|p_{3}\rangle$, and the remaining λ+1 copies are retained for Step (S6). The main operations are quantum state recovery and λ independent swap tests, with a computational complexity of O(λ).

Step (S6): It consumes partial segments of $K_{BT}$, including 4-bit $K_{BT}^{\prime \prime \prime }$ and 8-bit $K_{BT}^{\left(4\right)}$. No extra persistent qubits are introduced. A total of 2(λ+1)=2λ+2 encrypted Bell particle qubits are transmitted, accompanied by λ+1 encrypted parameters $E_{K_{BT}^{\left(4\right)}}|X_{3}^{\left(j\right)}\rangle$, each transmitted as a two-qubit quantum state, adding 2(λ+1)=2λ+2 qubits. The total quantum communication in this step is therefore 4λ+4 qubits. The step integrates Bell measurement and QOTP encryption, with an overall computational complexity of O(λ).

Step (S7): No key resources and persistent qubits are consumed, with zero communication overhead of quantum and classical channels. In actual execution, Bob receives λ+1 copies of $|p_{1}\rangle$. He uses λ copies in the swap test with $|p_{2}\rangle$ and retains the remaining one copy as the signed quantum message. The core operations are Pauli correction and λ independent swap tests, and the computational complexity is O(λ).

As above, the stepwise resource overhead summary of the proposed scheme is presented in Table 2. By the same approach, we derive the resource overhead of the Grover-based scheme (i.e., YKLY’2015), as listed in Table 3.

The proposed scheme differs architecturally from YKLY’2015 in several respects. The proposed scheme introduces persistent static qubits and consumes more key material (O(λ) bits per execution vs. 4 bits in YKLY’2015). These additional costs are incurred in exchange for stronger security guarantees. Unlike YKLY’2015, which is vulnerable to man-in-the-middle attacks that enable Alice’s disavowal and Bob’s forgery, the proposed scheme achieves unforgeability and non-repudiation through the combined use of strengthened QOTP encryption and arbitrated teleportation-based verification. The higher key consumption reflects the additional encryption and authentication steps required to protect against these attacks. Furthermore, quantum teleportation is adopted to reuse quantum channels, reducing the need for repeated quantum state transmissions.

The above analysis considers a single-qubit message. For a general *n*-qubit message |p〉, the resource requirements scale as follows.

**Key consumption.** Each qubit of the message requires independent encryption via the strengthened QOTP, which consumes a constant number of key bits per qubit. Specifically, for an *n*-qubit message, Alice must encrypt 4λ+1 physical copies of the message and the Bell state particle pairs used in teleportation. The $X_{i}$ parameters each become 2n-bit classical values for an *n*-qubit message. There are 5λ+3 such parameters in total: 2λ+1 values of $X_{1}$, 2λ+1 values of $X_{2}$, and λ+1 values of $X_{3}$. Their encryption cost therefore scales as O(λn). The total key consumption therefore scales as O(λn), with a dominant contribution coming from the QOTP encryption of the *n*-qubit message copies.

**Static qubits.** Each teleportation of an *n*-qubit message consumes *n* Bell states. The protocol involves 5λ+3 rounds of teleportation, requiring (5λ+3)n Bell states in total. Each Bell state occupies 2 qubits, yielding 2(5λ+3)n static qubits. The total number of static qubits therefore scales as O(λn).

**Dynamic qubits.** Alice prepares 4λ+1 physical copies of the *n*-qubit message, occupying (4λ+1)n dynamic qubits. During teleportation, each message qubit temporarily interacts with one half of a Bell state, but no additional persistent storage is required beyond the message copies themselves. The total dynamic qubit requirement is therefore O(λn).

**Quantum communication.** In Step (S3), Alice transmits the message group $|p_{2}\rangle$ (λn qubits) to Bob and the encrypted message group $E_{K_{AT}^{\prime }}|p_{3}\rangle$ (λn qubits) to Trent. In addition, the Bell state particle pairs transmitted in Steps (S3), (S4), and (S6) scale as O(λn) in total, since these steps involve 2λ+1, 2λ+1, and λ+1 teleportations, respectively. The total quantum communication overhead is therefore O(λn), dominated by the transmission of the message groups.

**Classical communication.** The QKD initialization step (I1) requires O(λn) classical bits for key distribution, as the total key length scales with λn. All encrypted $X_{i}$ parameters are transmitted as quantum states and are therefore counted in the quantum communication overhead rather than the classical communication overhead. The total classical communication overhead is therefore O(λn), dominated by QKD key distribution.

**Computational complexity.** The dominant operations are Bell measurements, QOTP encryption and decryption, and swap tests. For an *n*-qubit message, Bell measurements are performed qubit-wise on each of the 5λ+3 teleportation rounds, requiring O(λn) operations. The QOTP encryption applies Pauli gates independently to each qubit of the 4λ+1 physical copies, scaling as O(λn). Each swap test compares *n*-qubit registers using *n* controlled-SWAP gates, and the test is repeated λ times per comparison. Two comparisons are performed (in Steps S5 and S7), yielding O(λn) overall. The total computational complexity is therefore O(λn).

In summary, for an *n*-qubit message, key consumption, qubit overhead, communication requirements, and computational complexity all scale as O(λn). This scaling remains linear in the message length *n*, which is a natural consequence of processing each qubit individually. Table 4 summarizes the asymptotic resource scaling. Since λ is determined by the required security level and $F_{\max}$ is an application-level threshold chosen according to the required verification strictness, both are fixed once the security level and the application scenario are specified. Consequently, all resource requirements remain linear in the message length *n*.

### 5.2. Attack Simulations of the Proposed Scheme

To complement the formal security analysis of Section 4.3, we now analyze the strongest attacks that a dishonest Alice or Bob can mount against the proposed scheme, and illustrate their behavior through numerical modeling. By contrast, the vulnerabilities of YKLY’2015 follow directly from its protocol structure and require no simulation to verify.

#### 5.2.1. Alice’s Attack Simulation

For Alice’s repudiation, we construct the strongest possible attack, in which Alice deviates maximally in every step under her control.

Alice’s attack description. In Step (S3), Alice deviates from the protocol by sending a potentially forged state $|p_{2}^{\prime }\rangle$ to Bob and $E_{K_{AT}^{\prime }}|p_{3}^{\prime }\rangle$ to Trent, where $|p_{2}^{\prime }\rangle$ and $|p_{3}^{\prime }\rangle$ may differ from the honest copies. Simultaneously, she sends a forged particle pair with state $\left[I⊗E_{K_{AT}^{\prime \prime }}U\left(X_{1}^{\prime }\right)\right]|\varphi^{+}\rangle$ and $E_{K_{AT}^{\prime \prime \prime }}|X_{1}^{\prime }\rangle$ to Trent, where $X_{1}^{\prime }$ may differ from the true $X_{1}$.

Verification phase Analysis. In Step (S5), Trent decrypts and verifies the received particles. We analyze three possible scenarios depending on Bob’s behavior, since Alice does not control whether Bob is honest.

Scenario 1: Bob is honest. Bob follows the protocol (G=I, $X_{2}^{\prime }=X_{2}$). Trent measures $X_{1}^{\prime }$ from Alice’s particle pair and compares it with the decrypted $|X_{1}^{\prime }\rangle$. If they match, Trent applies $U(X_{1}^{\prime })$ and $U(X_{2})$ to the state $U\left(X_{2}\right)U\left(X_{1}\right)|p_{1}\rangle$. The recovered state is $|p_{1}^{\mathrm{rec}}\rangle =U\left(X_{1}^{\prime }\right)U\left(X_{1}\right)|p_{1}\rangle$ (up to a global phase). The swap test between $|p_{1}^{\mathrm{rec}}\rangle$ and $|p_{3}^{\prime }\rangle$ yields the condition

(6) $$|p_{1}^{\mathrm{rec}}\rangle ≅|p_{3}^{\prime }\rangle ≅U\left(X_{1}^{\prime }\right)U\left(X_{1}\right)|p_{1}\rangle .$$

This corresponds to the stronger malicious-signing adversary considered in the non-repudiation analysis of Section 4.3, where Alice is allowed to deviate during signature generation.

Scenario 2: Bob is dishonest, but his attack prevents the protocol from proceeding. Bob applies G≠I or sends $X_{2}^{\prime }\neq X_{2}$. If $U\left(X_{2}^{\prime }\right)U\left(X_{2}\right)G\neq I$, the fidelity *F* between the state recovered by Trent and the reference state $|p_{3}\rangle$ satisfies F<1, and the swap test detects the mismatch with probability $1-{\left[\left(1+F\right)/2\right]}^{\lambda }$. Unless Bob’s deviation is chosen such that F≈1, this probability is close to 1, causing the protocol to abort. In this case, Bob has not accepted any signature, so Alice has nothing to repudiate. Her attack fails trivially.

Scenario 3: Bob is dishonest, but his attack inadvertently assists Alice. Bob applies G≠I and/or sends $X_{2}^{\prime }\neq X_{2}$, but the combination happens to satisfy $U\left(X_{2}^{\prime }\right)U\left(X_{2}\right)G=I$. In this case, the swap test passes and the protocol proceeds despite Bob’s deviation. Table 5 shows the required *G* for each pair $\left(X_{2}^{\prime },X_{2}\right)$. For a given pair $\left(X_{2}^{\prime },X_{2}\right)$, Bob’s deviation *G* is chosen randomly from $\left\{I,\sigma_{x},\sigma_{y},\sigma_{z}\right\}$, and exactly one of these four choices satisfies $U\left(X_{2}^{\prime }\right)U\left(X_{2}\right)G=I$. Hence, if Bob chooses *G* uniformly at random, the probability that his deviation inadvertently allows the protocol to proceed is 1/4. Even in this scenario, once the protocol proceeds to Step (S7) and Bob accepts, the traceability argument of Scenario 1 applies, and Trent can still link the accepted message to Alice.

In practical systems, non-attack factors such as channel noise, device imperfections, and environmental disturbances introduce an additional baseline error rate, denoted by α. In Scenario 1 (Bob honest), the protocol proceeds deterministically from Alice’s perspective, and the only source of error is the finite failure probability ${\left[\left(1+F\right)/2\right]}^{\lambda }$ of the swap test, which is bounded by ${\left[\left(1+F_{\max}\right)/2\right]}^{\lambda }$ and reduces to $2^{-\lambda }$ when $F_{\max}=0$. In Scenario 3 (Bob inadvertently assists Alice), the protocol proceeds with ideal probability 1/4 as shown above. Taking the worst-case view that Scenario 3 occurs, the total probability that Trent observes an erroneous acceptance is modeled as $p=\frac{1}{4}+\alpha$, where the 1/4 term accounts for the probability that Bob’s random deviation accidentally satisfies $U\left(X_{2}^{\prime }\right)U\left(X_{2}\right)G=I$, and α is the baseline error rate due to physical noise, treated as an independent additive contribution in this phenomenological model. A detection threshold β is set: the transmission is accepted if the observed bit-error rate $\hat{\varepsilon }\leq \beta$, and rejected otherwise. The probability that Alice’s attack succeeds (remains undetected) is $P_{\mathrm{success}}\left(n\right)=\mathrm{Pr}\left(\hat{\varepsilon }\leq \beta \;|\;p=\frac{1}{4}+\alpha \right).$ For *n* transmitted quantum bits, let the observed number of erroneous bits be *k*. Then *k* follows a binomial distribution k∼Binomial(n,p), and the observed bit-error rate is $\hat{\varepsilon }=k/n$. Hence,

(7) Psuccess(n)=∑k=0⌊nβ⌋nk14+αk34−αn−k.

The security requirements is p>β. The condition p>β ensures that the bit-error rate induced by Alice’s attack exceeds the detection threshold, meaning the attack leaves a detectable trace and is therefore likely to be rejected by the system. Consequently, the attack success probability $P_{\mathrm{success}}\left(n\right)$ represents the probability that the system accepts the transmission despite an actual attack being present, i.e., the false negative rate. By Chernoff’s bound for the binomial distribution, for β<1/4+α,

(8) $$P_{\mathrm{success}}\left(n\right)\leq \exp\left[-n\left(\beta \ln\frac{\beta }{1/4+\alpha }+\left(1-\beta \right)\ln\frac{1-\beta }{3/4-\alpha }\right)\right].$$

**Figure 5:**
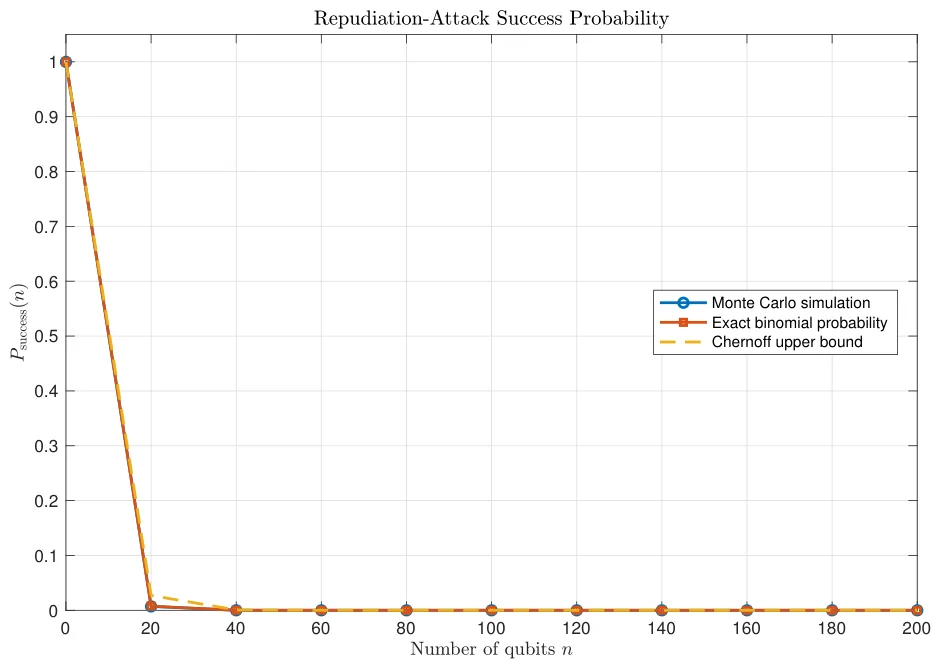
Attack success probability versus the number of qubits.

For clarity, the upper bound on Alice’s attack success probability as a function of the number of quantum bits *n* is shown in Figure 5. We adopt the Monte-Carlo method to conduct numerical simulations. For each value of qubit number *n*, we perform 20,000 random sampling experiments obeying binomial distribution k∼Binomial(n,0.30). We judge that the attack succeeds when k≤nβ with β=0.06. It can be seen that the simulation results fit well with the theoretical binomial expression, which confirms the consistency of the numerical implementation with the probabilistic model. The MATLAB R2024a code implementing the Monte-Carlo simulation is given in the Supplementary Material.

Finally, this constructed attack represents the strongest possible repudiation attempt, in which Alice deviates maximally in every step under her control. The analysis above covers three scenarios. If Bob is honest (Scenario 1), the protocol proceeds and Trent can trace the accepted message to Alice. If Bob’s deviation prevents the protocol from proceeding (Scenario 2), Alice has no accepted signature to repudiate. If Bob’s deviation inadvertently assists Alice (Scenario 3, probability 1/4), the protocol proceeds and Trent’s traceability still holds. Therefore, in all possible scenarios, Alice cannot successfully repudiate a signature that Bob has accepted.

#### 5.2.2. Bob’s Attack Simulation

We now analyze Bob’s forgery attack under the assumption that Alice follows the protocol honestly. This corresponds to the standard unforgeability game in Section 4.3. If Alice were also dishonest, the analysis of Scenario 2 and Scenario 3 in the previous subsection applies symmetrically: Alice’s deviation would either cause the protocol to abort or, with small probability, inadvertently assist Bob. In either case, Bob’s forgery success remains bounded by the same arguments.

Bob’s attack description. Bob has two attack opportunities. In the first stage (during protocol execution, Step (S3)–(S4)), Bob possesses $|p_{2}\rangle$ and $U\left(X_{1}\right)|p_{1}\rangle$. He may apply a non-identity operation *G* on $U\left(X_{1}\right)|p_{1}\rangle$ before teleporting it to Trent in Step (S4), or modify the plaintext copy $|p_{2}\rangle$. In the second stage (after protocol completion, Step (S7)), Bob holds $|p_{2}\rangle$ and $U\left(X_{3}\right)|p_{1}\rangle$, and may attempt to produce a forged signature.

Verification phase Analysis. For the first stage, if Bob applies G≠I, the state reaching Trent becomes $U\left(X_{2}\right)GU\left(X_{1}\right)|p_{1}\rangle$. After Trent applies $U(X_{1})$ and $U(X_{2})$ in Step (S5), the recovered state is $U\left(X_{1}\right)GU\left(X_{1}\right)|p_{1}\rangle$ (up to a global phase). Trent compares this with $|p_{3}\rangle$ via the swap test. Since $|p_{3}\rangle$ was encrypted by Alice with $K_{AT}^{\prime }$, Bob cannot alter it. The swap test detects the mismatch with probability at least $1-{\left[\left(1+F_{G}\right)/2\right]}^{\lambda }$, where $F_{G}$ is the fidelity between the tampered state and the reference state. For orthogonal states ($F_{G}=0$), this reduces to $1-2^{-\lambda }$. If Bob modifies $|p_{2}\rangle$ instead, the swap test in Step (S7) detects the mismatch with the same probability guarantee.

For the second stage, after recovering $|p_{1}\rangle$ and verifying it against $|p_{2}\rangle$, Bob already holds the valid message. To forge a different message, Bob would need to produce a new $|p^{*}\rangle$ and corresponding evidence that Trent accepts as originating from Alice. However, Trent retains the encrypted record $E_{K_{AT}^{\prime }}|p_{3}\rangle$ and the particle pairs from both Alice and Bob, all protected by $K_{AT}$ that Bob does not possess. Bob’s knowledge of $X_{2}$ and $X_{3}$ provides no advantage, as these are random Bell measurement outcomes independent of the message content.

In summary, the above analysis shows that neither opportunity allows Bob to forge Alice’s signature with non-negligible probability. This is consistent with the formal unforgeability proof in Section 4.3.

### 5.3. Numerical Simulations

To complement the analytical security discussion, we performed numerical and hardware evaluations of the verification model. Reference implementations are provided in the Supplementary Material. The numerical simulations and hardware-evaluation workflows were implemented in Python 3.12.5 using Qiskit 2.0.0, qiskit-aer 0.17.0, qiskit-ibm-runtime 0.40, NumPy 2.1.1, pandas 3.0.2, and Matplotlib 3.10.1. IBM Quantum hardware jobs were executed through Qiskit Runtime using QiskitRuntimeService and SamplerV2.

**Figure 6:**
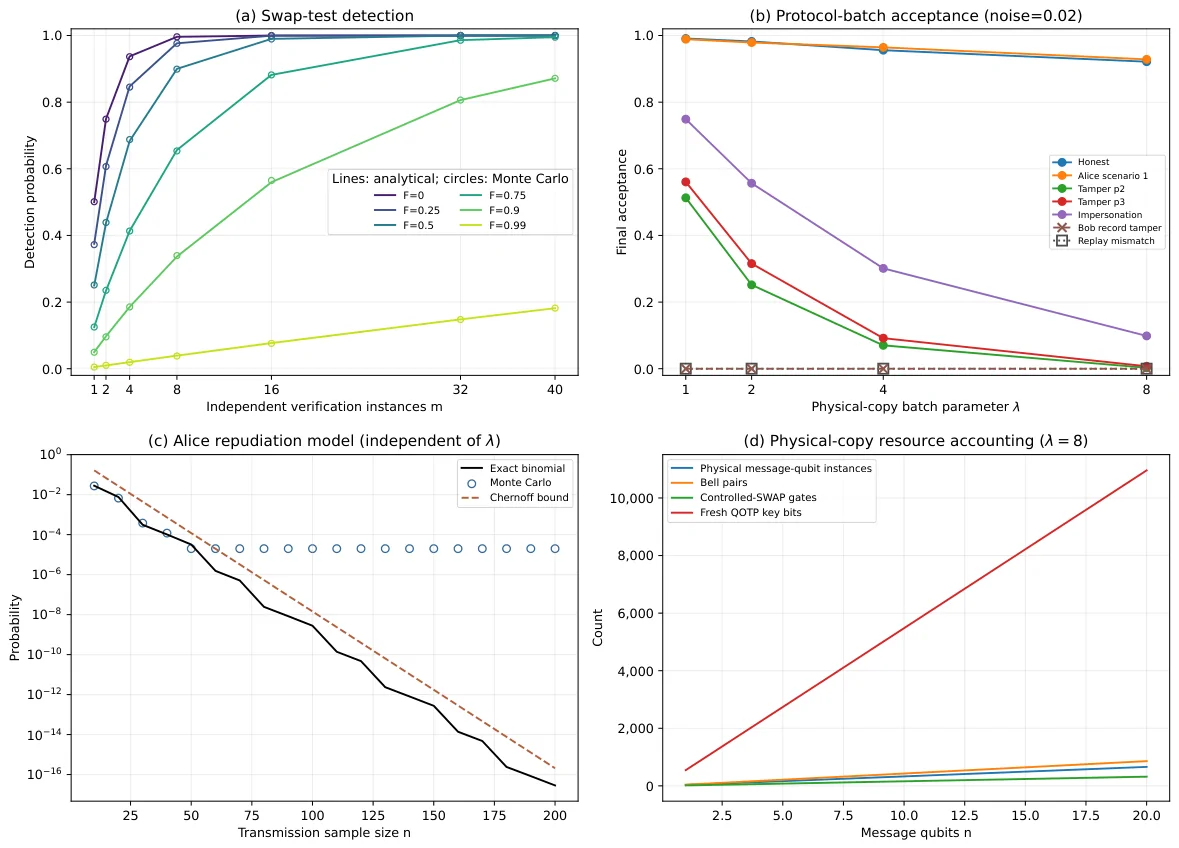
Protocol-batch performance and resource accounting. (**a**) Swap-test detection probability versus the number *m* of independent Swap-test comparisons for several fidelity values *F*. Lines are analytical results; circles are Monte Carlo simulations. (**b**) Final acceptance probability under honest execution and multiple attack scenarios versus the physical-copy batch parameter λ, under a simplified state-level noise model with noise parameter 0.02. Honest execution and Alice scenario 1 remain statistically close in this consistency-only diagnostic. The Bob record-tampering and replay-mismatch curves overlap at zero acceptance. (**c**) Alice’s repudiation probability versus the transmission sample size *n* under the independent binomial model. Exact binomial, Monte Carlo, and Chernoff-bound results are compared. (**d**) Cumulative physical-resource counts versus message size *n* for fixed batch parameter λ=8.

Figure 6 summarizes the batch-mode performance and resource overhead. Figure 6a shows the detection probability as a function of the number of verification instances *m* for fidelity values *F* from 0 to 0.99. The analytical curves agree with Monte Carlo simulations across the tested range. For small and moderate *F*, the detection probability increases rapidly with *m*; however, when *F* approaches unity, such as F=0.99, the detection rate remains limited even at m=40, reflecting the inherent difficulty of distinguishing near-identical states. Figure 6b shows that as the batch parameter λ grows, the acceptance probabilities of the modeled tampering, impersonation, record-tampering, and replay-mismatch scenarios generally decrease, while the honest acceptance probability remains high. Alice scenario 1 remains statistically close to honest execution in this consistency-only diagnostic. The suppression is quantitative rather than absolute: the modeled attack-acceptance probabilities become small but do not necessarily reach zero for finite batch sizes. Figure 6c compares the exact binomial probability, the seeded Monte Carlo estimate, and the Chernoff upper bound for the separate statistical model of Alice’s repudiation behavior. Figure 6d quantifies the cumulative physical-resource scaling versus the number of message qubits for a fixed batch parameter λ=8, under the stated resource-counting definitions.

We note three limitations of the numerical evaluation. First, the $1-2^{-m}$ detection formula applies only to orthogonal inputs with F=0; for high-fidelity cases such as F=0.99, the detection probability grows much more slowly with *m*, and this should not be interpreted as a general closed-form detection law. Second, the batch-level curves are evaluated in the physical-copy batch model under a simplified state-level noise model, in which each relevant state is subjected to a fixed noise level. This model is not a device-level noise simulation and does not correspond to independent protocol instances. Under nonzero noise, even the honest acceptance probability decreases with λ, because every selected comparison must pass. Third, the binomial curve for Alice’s repudiation probability in Figure 6c is a statistical illustration under an independent binomial model; it does not by itself establish non-repudiation, which relies on the full protocol records and the adversarial analysis of Section 4.3.

**Figure 7:**
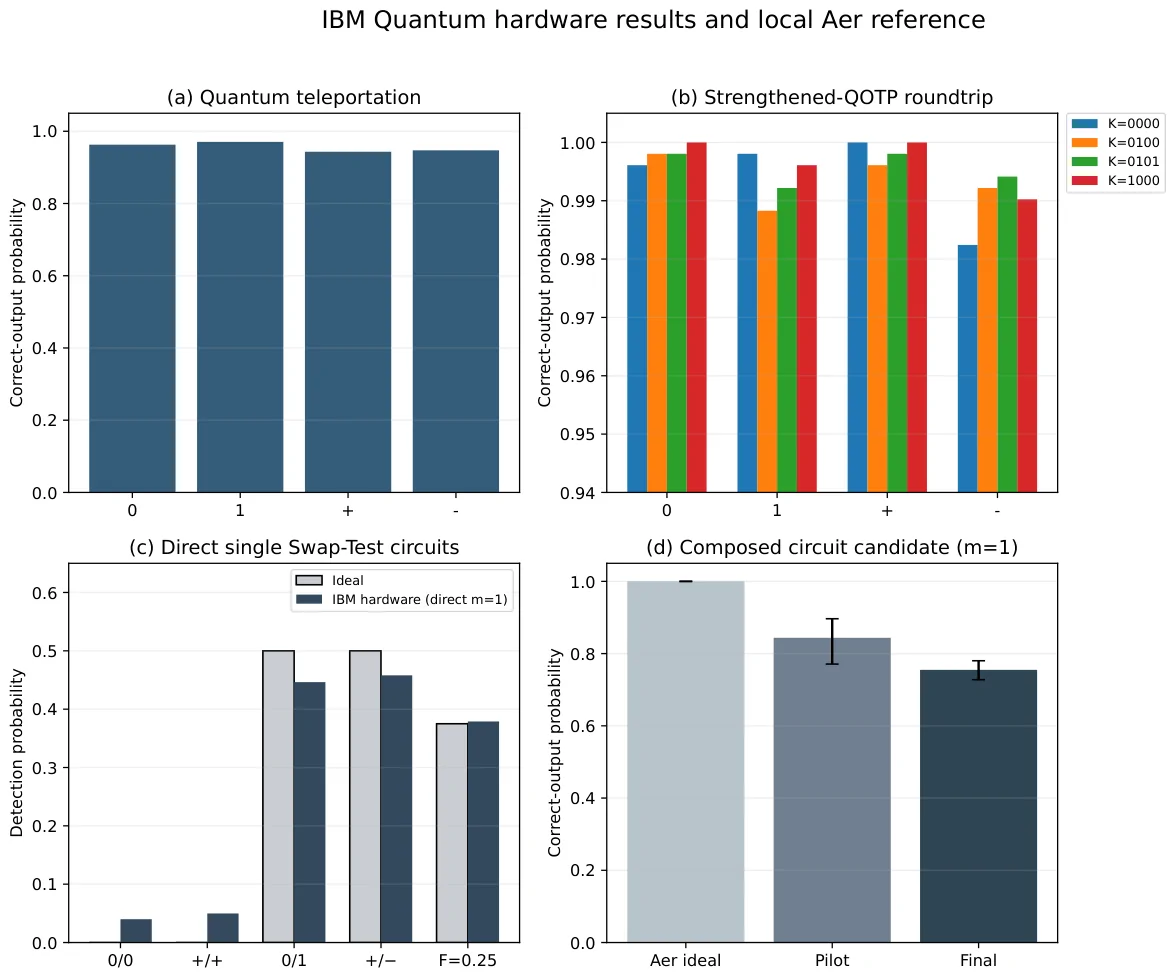
Circuit-level hardware evaluation of core quantum primitives on IBM Quantum devices and local simulations. (**a**) Correct-output probability for quantum teleportation under different input states. (**b**) Correct-output probability for the strengthened-QOTP roundtrip with various keys. (**c**) Detection probability for direct single Swap-test circuits, comparing ideal, local Aer simulation, and real hardware results. (**d**) Correct-output probability comparison for a composed circuit-level candidate corresponding to a single verification instance (m=1), evaluated using IBM Quantum hardware and a local Aer reference.

Figure 7 presents circuit-level hardware evaluation results obtained from IBM Quantum devices and local simulations. Figure 7a,b show that quantum teleportation and the strengthened-QOTP roundtrip achieve correct-output probabilities mostly above 0.94 across different input states and keys, indicating basic feasibility despite noticeable hardware noise. Figure 7c reveals a significant gap between ideal/simulated and real-hardware Swap-test detection, highlighting the sensitivity of this operation to current-device noise. Figure 7d further shows that an optimized composed circuit candidate with batch parameter m=1 performs acceptably on hardware, albeit below simulator levels. These hardware runs are circuit-level benchmarks of the protocol’s core primitives; they do not constitute a full implementation of the complete (5λ+3)-Bell-pair physical-copy batch protocol. In particular, the Swap-test gap between simulation and hardware indicates that direct deployment of the full batch protocol would require either noise suppression or additional error mitigation, and the present experiments should not be taken as an end-to-end hardware demonstration.

Overall, these evaluations show that the verification behavior is consistent with the fidelity-based analysis for the tested instances, that the batch structure and resource accounting follow the stated numerical model, and that the selected circuit-level quantum operations can be executed on current noisy quantum devices at a small scale.

## 6. Conclusions

This paper addressed the structural security challenges that arise when quantum search primitives are directly embedded into signature workflows. We identified structural vulnerabilities in a Grover-based quantum signature scheme and demonstrated that these vulnerabilities enable complete key recovery, Alice’s disavowal, and Bob’s existential forgery via man-in-the-middle attacks. To address these weaknesses, we proposed an arbitrated quantum signature scheme based on quantum teleportation and a strengthened quantum one-time pad. We emphasize that the scheme targets quantum messages with known classical descriptions, and is not applicable to arbitrary unknown quantum states. The security of the proposed scheme was analyzed through a formal adversarial model with well-defined security games for unforgeability and non-repudiation, and its resilience against replay, intercept-and-resend, man-in-the-middle, entanglement, and collective or coherent attacks was examined. Numerical simulations illustrated the behavior of the swap test as a verification primitive and the linear scaling of resource consumption.

Beyond signature schemes, future work may further extend quantum search algorithms to other quantum cryptographic protocols or multi-party quantum communication scenarios. Potential directions include routing optimization, repeater selection, and network diagnostics, where quantum speedups hold promise for reducing latency and enhancing resource efficiency. These avenues warrant further investigation within the context of future quantum networks.

## Figures and Tables

**Table 1:** Summary of key notation.

General		Primitives		Protocol	
C	Complex numbers	$\delta_{x},\delta_{y},\delta_{z}$	Pauli operators	$K_{AT}$(16)	Key (Alice-Trent)
i	Imaginary unit	*F*	State fidelity	$K_{BT}$(24)	Key (Bob-Trent)
≅	Equal up to phase	|p〉	Message state	$K_{AT}^{\prime }$(4) etc.	Key segments
α	Physical error rate	$E_{K}|p\rangle$	Strengthened QOTP	$X_{1},X_{2},X_{3}$	Bell outcomes
λ	Security parameter	$E_{k}\left(\rho \right)$	Standard QOTP	$|p_{1}\rangle ,|p_{2}\rangle ,|p_{3}\rangle$	Message copies
$K^{i}$	*i*-th bit of *K*	U(X)	Pauli operation	$|p_{2}^{\prime }\rangle ,|p_{3}^{\prime }\rangle ,X_{1}^{\prime }$	Forged values
|X〉	State for *n*-bit *X*	$|\varphi^{\pm }\rangle ,|\psi^{\pm }\rangle$	Bell states	$p_{\mathrm{dep}}$	Depolarizing noise
$t_{\mathrm{comm}},t_{\mathrm{op}}$	Latency, local time	$|\varphi^{+}\rangle_{AB},{|\varphi^{+}\rangle }_{BT}$	Shared Bell pairs		

**Table 2:** Stepwise resource overhead summary of the proposed scheme.

Index	I1	I2	S1	S2	S3	S4	S5	S6	S7	Total
Key Consumed (bit)	O(λ)	0	0	0	16	12	0	12	0	O(λ)
Static and Dynamic Qubits	0	2(5λ+3)	4λ+1	0	0	0	0	0	0	14λ+7
Quantum Transmit (qubit)	0	7λ+4	0	0	10λ+4	8λ+4	0	4λ+4	0	29λ+16
Classical Transmit (bit)	O(λ)	0	0	0	0	0	0	0	0	O(λ)
Computational Complexity	O(λ)	O(λ)	O(λ)	O(λ)	O(λ)	O(λ)	O(λ)	O(λ)	O(λ)	O(λ)

Notes: The O(λ) secret key bits are freshly generated via QKD for each protocol execution. Each key segment is used exactly once and is never reused. Maximum number of simultaneously occupied valid qubits throughout a complete protocol execution cycle (values are non-cumulative). Quantum/Classical Transmission: Cumulative qubits/classical bits transmitted over all protocol stages, including communication for QKD initialization. Computational Complexity: Asymptotic complexity for all quantum and classical operations within each step.

**Table 3:** Stepwise resource overhead summary of YKLY’2015.

Index	AT Total (AT1–AT8)	TB Total (TB1–TB8)	Overall Total
Key Consumed (bit)	2	2	4
Dynamic Qubits Used	8	8	8
Quantum Transmit (qubit)	8	8	16
Classical Transmit (bit)	13	13	26
Computational Complexity	O(1)	O(1)	O(1)

Notes: Key Consumption: New secret key bits generated in each stage; the total row accumulates all independent QKD keys $K_{AT}$ and $K_{TB}$. Dynamic Qubit Consumption: Temporary qubits occupied within each step (values are non-cumulative). Quantum/Classical Transmission: Cumulative transmitted qubits and classical bits within each step. Computational Complexity: Asymptotic complexity of all quantum and classical operations within each step.

**Table 4:** Asymptotic resource scaling for an *n*-qubit message.

Resource	Scaling
Key consumption	O(λn)
Static qubits	O(λn)
Dynamic qubits	O(λn)
Quantum communication	O(λn)
Classical communication	O(λn)
Computational complexity	O(λn)

**Table 5:** Required operation *G* for the protocol to proceed when Bob deviates by sending $X_{2}^{\prime }$ instead of the true $X_{2}$. The diagonal entries ($X_{2}^{\prime }=X_{2}$) correspond to G=I, i.e., Bob does not apply any additional operation.

$X_{2}^{\prime }\setminus X_{2}$	00	01	10	11
00	*I*	$\sigma_{x}$	$\sigma_{z}$	$\sigma_{y}$
01	$\sigma_{x}$	*I*	$\sigma_{y}$	$\sigma_{z}$
10	$\sigma_{z}$	$\sigma_{y}$	*I*	$\sigma_{x}$
11	$\sigma_{y}$	$\sigma_{z}$	$\sigma_{x}$	*I*

## Data Availability

The code supporting the results is available in the Supplementary Materials. Further inquiries can be directed to the corresponding author.

## Supplementary Materials

The following supporting information can be downloaded at https://www.mdpi.com/article/10.3390/e28091030/s1. File S1: Full Code of Figure 5; File S2: Full Code of Figure 6; File S3: Full Code of Figure 7.
