# Supplementary material for: On a Grover-Based Quantum Signature Scheme and a Teleportation-Based Design
Source: Entropy (Basel). 2026 Sep 18;28(9):1030. doi: 10.3390/e28091030 (PMC13605562; doi:10.3390/e28091030)
Supplement: Supplementary file 1 [file entropy-28-01030-s001.zip › Supplementary Material-S1-Figure_5_MATLAB code.pdf]

The following MATLAB code generates Figure 5.

```
% Clear workspace
>> %% Parameter setting
alpha = 0.05;
beta    = 0.06;
p          = 0.25 + alpha;
trials = 20000;
n_list = 0:20:200;
sim_result = zeros(size(n_list));
theory_result = zeros(size(n_list));
for idx = 1:length(n_list)
        n = n_list(idx);
        threshold = floor(n * beta);
        success_case = 0;
        for t = 1:trials
                k = binornd(n,p);
                if(k <= threshold)
                        success_case = success_case + 1;
                end
        end
        sim_result(idx) = success_case / trials;
        term1 = beta * log(beta / p);
        term2 = (1 - beta) * log((1 - beta) / (1 - p));
        theory_result(idx) = exp(-n * (term1 + term2));
end
%% Plot figure
figure('Position',[100,100,640,400])
plot(n_list,sim_result,'o-','LineWidth',2,'DisplayName','Monte-Carlo Numerical Simulation');
hold on;
plot(n_list,theory_result,'--','LineWidth',2,'DisplayName','Chernoff            Bound            Theoretical
Upper-bound');
xlabel('Number of qubits $n$','Interpreter','latex');
ylabel('$P_{success}(n)$','Interpreter','latex');
title('Attack Success Probability: Simulation vs Theoretical Results');
legend;
grid on;
>>
```
