# Supplementary material for: On a Grover-Based Quantum Signature Scheme and a Teleportation-Based Design
Source: Entropy (Basel). 2026 Sep 18;28(9):1030. doi: 10.3390/e28091030 (PMC13605562; doi:10.3390/e28091030)
Supplement: Supplementary file 1 [file entropy-28-01030-s001.zip › Supplementary Material-S2-Figure_6_Python code.pdf]

Qiskit Simulation

local_reference_evaluation.py

```
"""Offline local validation for the organized verification code."""

from __future__ import annotations

import json
import math
from pathlib import Path

import numpy as np
from qiskit import QuantumCircuit, transpile
from qiskit.providers.basic_provider import BasicSimulator
from qiskit.quantum_info import Statevector, partial_trace, state_fidelity
from qiskit_aer import AerSimulator

from qiskit_protocol_reference import (
    build_composed_staged_emulation_candidate,
    coherent_teleportation,
    composed_candidate_expected_outputs,
    prepare_named_state,
    success_probability_from_counts,
)
from verification_experiment_suite import SEED, fidelity, write_rows


VALIDATION_STATES = ("0", "1", "+", "-", "i+", "i-")
COMPOSED_STATES = ("0", "1", "+", "-", "i+", "i-", "global_phase_i+")
STAGES = ("S2", "S4", "S6")


def named_statevector(label: str) -> np.ndarray:
    if label == "0":
        return np.array([1, 0], dtype=complex)
    if label == "1":
        return np.array([0, 1], dtype=complex)
    if label == "+":
        return np.array([1, 1], dtype=complex) / np.sqrt(2)
    if label == "-":
        return np.array([1, -1], dtype=complex) / np.sqrt(2)
    if label == "i+":
        return np.array([1, 1j], dtype=complex) / np.sqrt(2)
    if label == "i-":
        return np.array([1, -1j], dtype=complex) / np.sqrt(2)
    raise ValueError(f"unsupported validation state: {label}")


def coherent_chain_fidelity(input_state: str, rounds: int) -> float:
    if rounds not in (1, 2, 3):
        raise ValueError("rounds must be 1, 2, or 3")
    qc = QuantumCircuit(1 + 2 * rounds)
    prepare_named_state(qc, 0, input_state)
    for round_index in range(rounds):
        sender = 1 + 2 * round_index
        receiver = sender + 1
        qc.h(sender)
        qc.cx(sender, receiver)
        source = 0 if round_index == 0 else 2 * round_index
        coherent_teleportation(qc, source, sender, receiver, STAGES[round_index])
    state = Statevector.from_instruction(qc)
    receiver = 2 * rounds
    reduced = partial_trace(state, [q for q in range(qc.num_qubits) if q != receiver])
    return float(np.clip(state_fidelity(reduced, named_statevector(input_state)), 0.0, 1.0))


def conditioned_outcome_fidelities(input_state: str) -> dict[str, float]:
    """Exact Bell-outcome branches with X^b then Z^a receiver corrections."""
    target = named_statevector(input_state)
    qc = QuantumCircuit(3)
    prepare_named_state(qc, 0, input_state)
    qc.h(1)
    qc.cx(1, 2)
```

```python
    qc.cx(0, 1)
    qc.h(0)
    tensor = np.asarray(Statevector.from_instruction(qc)).reshape(2, 2, 2)
    X = np.array([[0, 1], [1, 0]], dtype=complex)
    Z = np.array([[1, 0], [0, -1]], dtype=complex)
    results = {}
    for message_bit in (0, 1):
        for sender_bit in (0, 1):
            receiver = tensor[:, sender_bit, message_bit]
            norm = np.linalg.norm(receiver)
            if norm <= 0:
                raise RuntimeError("zero-probability teleportation branch")
            receiver = receiver / norm
            if sender_bit:
                receiver = X @ receiver
            if message_bit:
                receiver = Z @ receiver
            results[f"{message_bit}{sender_bit}"] = fidelity(target, receiver)
    return results


def generate_teleportation_regression(path: Path) -> None:
    rows = []
    for state_name in VALIDATION_STATES:
        conditioned = conditioned_outcome_fidelities(state_name)
        phase_fidelity = fidelity(named_statevector(state_name), np.exp(1j * 0.731) * named_statevector(state_name))
        for rounds, stage in enumerate(STAGES, start=1):
            rows.append(
                {
                    "input_state": state_name,
                    "stage": stage,
                    "sequential_rounds": rounds,
                    "coherent_deferred_measurement_fidelity": coherent_chain_fidelity(state_name, rounds),
                    "measurement_conditioned_min_fidelity": min(conditioned.values()),
                    "measurement_conditioned_branch_fidelities_json": json.dumps(conditioned, sort_keys=True),
                    "global_phase_invariance_fidelity": phase_fidelity,
                    "expected_fidelity": 1.0,
                }
            )
    write_rows(path, rows)


def _candidate_for_label(label: str):
    if label == "global_phase_i+":
        circuit = build_composed_staged_emulation_candidate("i+")
        circuit.global_phase += math.pi / 3
        circuit.metadata = dict(circuit.metadata)
        circuit.metadata["input_state"] = label
        return circuit, "i+"
    return build_composed_staged_emulation_candidate(label), label


def generate_composed_candidate_reference(path: Path, shots: int = 4096) -> None:
    rows = []
    engines = (("BasicSimulator", BasicSimulator()), ("AerSimulator-ideal", AerSimulator()))
    for engine_index, (engine_name, backend) in enumerate(engines):
        for state_index, label in enumerate(COMPOSED_STATES):
            for m in (1, 2, 4, 8):
                instance_probabilities = []
                instance_counts = []
                valid_outputs = None
                for j in range(m):
                    circuit, semantic_label = _candidate_for_label(label)
                    valid_outputs = composed_candidate_expected_outputs(semantic_label)
                    seed = SEED + engine_index * 100_000 + state_index * 1_000 + m * 10 + j
                    executable = transpile(circuit, backend, optimization_level=1, seed_transpiler=seed)
                    counts = backend.run(executable, shots=shots, seed_simulator=seed).result().get_counts()
                    instance_counts.append(counts)
                    instance_probabilities.append(success_probability_from_counts(counts, valid_outputs))
                logical_probability = float(np.prod(instance_probabilities))
                dominant_probability = float(np.prod([max(item.values()) / shots for item in instance_counts]))
                rows.append(
                    {
                        "engine": engine_name,
                        "input_state": label,
```

```
                        "m": m,
                        "shots_per_instance": shots,
                        "circuit_instances": m,
                        "valid_expected_outputs": json.dumps(sorted(valid_outputs)),
                        "expected_measurement_distribution": json.dumps({value: 1.0 for value in sorted(valid_outputs)}),
                        "dominant_output_probability": dominant_probability,
                        "logical_success_probability": logical_probability,
                        "absolute_difference_between_metrics": abs(logical_probability - dominant_probability),
                        "relative_difference_between_metrics": abs(logical_probability - dominant_probability) /
dominant_probability if dominant_probability else math.nan,
                        "interpretation": "Logical success is evaluated over the input-dependent valid-output set; the
dominant output is a separate diagnostic.",
                        "counts_per_instance_json": json.dumps(instance_counts, sort_keys=True),
                    }
                )
    write_rows(path, rows)
```

qiskit_circuit_library.py

```
"""Complete IBM/Qiskit circuit, retrieval, evaluation, and guarded-submission code."""

from __future__ import annotations

import argparse
import csv
import hashlib
import json
from math import sqrt
from pathlib import Path

from qiskit import ClassicalRegister, QuantumCircuit, QuantumRegister, transpile
from qiskit.qasm3 import dumps as qasm3_dumps
from qiskit.transpiler import generate_preset_pass_manager
try:
    from qiskit_ibm_runtime import QiskitRuntimeService, SamplerV2
except ImportError:  # Optional for local circuit construction.
    QiskitRuntimeService = None
    SamplerV2 = None


# 1. Bell resources

def build_bell_pair_circuit(name='bell_pair'):
    q = QuantumRegister(2, 'bell')
    qc = QuantumCircuit(q, name=name)
    qc.h(q[0])
    qc.cx(q[0], q[1])
    return qc

def build_three_bell_pair_circuit():
    q = QuantumRegister(6, 'bell')
    qc = QuantumCircuit(q, name='three_v7_bell_pairs')
    for a, b in ((0, 1), (2, 3), (4, 5)):
        qc.h(q[a])
        qc.cx(q[a], q[b])
    qc.metadata = {'pairs': {'AB_S2': [0, 1], 'BT_S4': [2, 3], 'TB_S6': [4, 5]}}
    return qc

# 2. Teleportation

def coherent_teleportation(qc, message, bell_sender, bell_receiver, label):
    qc.barrier(label=f'{label}_Bell_measurement')
    qc.cx(message, bell_sender)
    qc.h(message)
    qc.cx(bell_sender, bell_receiver)
    qc.cz(message, bell_receiver)

def build_teleportation_circuit(input_state='0'):
    qc = QuantumCircuit(3, 1, name=f'teleport_{input_state}')
    if input_state == '1':
        qc.x(0)
    elif input_state == '+':
        qc.h(0)
    elif input_state == '-':
        qc.x(0)
        qc.h(0)
    qc.h(1)
    qc.cx(1, 2)
    coherent_teleportation(qc, 0, 1, 2, 'S2')
    if input_state in {'+', '-'}:
        qc.h(2)
    qc.measure(2, 0)
    return qc

def build_three_teleportation_rounds(input_state='0'):
    qc = QuantumCircuit(7, 1, name='three_teleportation_rounds')
    if input_state == '1':
        qc.x(0)
    elif input_state == '+':
        qc.h(0)
    elif input_state == '-':
        qc.x(0)
```

```
        qc.h(0)
    for a, b in ((1, 2), (3, 4), (5, 6)):
        qc.h(a)
        qc.cx(a, b)
    coherent_teleportation(qc, 0, 1, 2, 'S2')
    coherent_teleportation(qc, 2, 3, 4, 'S4')
    coherent_teleportation(qc, 4, 5, 6, 'S6')
    if input_state in {'+', '-'}:
        qc.h(6)
    qc.measure(6, 0)
    return qc

# 3. Strengthened-QOTP

def append_qotp(qc, qubit, key4, label='E_K'):
    U = encryption_unitary(key4)
    qc.unitary(U, [qubit], label=label)

def append_qotp_inverse(qc, qubit, key4, label='E_K^-1'):
    U = encryption_unitary(key4)
    qc.unitary(U.conj().T, [qubit], label=label)

def build_qotp_roundtrip_circuit(key4='0101', input_state='0'):
    qc = QuantumCircuit(1, 1, name='strengthened_qotp_roundtrip')
    if input_state == '1':
        qc.x(0)
    elif input_state == '+':
        qc.h(0)
    elif input_state == '-':
        qc.x(0)
        qc.h(0)
    append_qotp(qc, 0, key4)
    append_qotp_inverse(qc, 0, key4)
    if input_state in {'+', '-'}:
        qc.h(0)
    qc.measure(0, 0)
    return qc

# 4. Encrypted Bell records

def append_encrypted_record_roundtrip(qc, carrier, key4, record, label):
    if record[1] == '1':
        qc.x(carrier)
    if record[0] == '1':
        qc.z(carrier)
    append_qotp(qc, carrier, key4, f'{label}_encrypt')
    append_qotp_inverse(qc, carrier, key4, f'{label}_decrypt')
    if record[0] == '1':
        qc.z(carrier)
    if record[1] == '1':
        qc.x(carrier)

def build_encrypted_bell_record_circuit(key4='1010', record='01'):
    qc = QuantumCircuit(2, 2, name='encrypted_bell_record_roundtrip')
    qc.h(0)
    qc.cx(0, 1)
    append_encrypted_record_roundtrip(qc, 1, key4, record, 'E_KU_X')
    qc.cx(0, 1)
    qc.h(0)
    qc.measure([0, 1], [0, 1])
    return qc

# 5. Repeated swap tests

def build_repeated_swap_test_circuits(left='0', right='1', lam=8):
    return [build_swap_test_circuit(left, right, name=f'swap_{index + 1}_of_{lam}') for index in range(lam)]

# 6. Staged components

def build_staged_components():
    return {'S2': build_teleportation_circuit(), 'S3_record': build_encrypted_bell_record_circuit(), 'S5_QOTP':
build_qotp_roundtrip_circuit(), 'S6': build_teleportation_circuit()}

# 7. Composed staged-emulation candidate
```

```
FIXED_KEY = '0101'

def _prepare_message_copies(qc, qubits, input_state):
    for q in qubits:
        if input_state == '1':
            qc.x(q)
        elif input_state == '+':
            qc.h(q)
        elif input_state == '-':
            qc.x(q)
            qc.h(q)

def _swap_test(qc, anc, left, right, label):
    qc.barrier(label=label)
    qc.h(anc)
    qc.cswap(anc, left, right)
    qc.h(anc)

def build_composed_staged_emulation_candidate(input_state='0'):
    q = QuantumRegister(9, 'candidate')
    c = ClassicalRegister(3, 'out')
    qc = QuantumCircuit(q, c, name='composed_staged_emulation_candidate')
    _prepare_message_copies(qc, (0, 1, 2), input_state)
    qc.barrier(label='S1_three_message_copies')
    for a, b, label in ((3, 4, 'AB_S2'), (5, 6, 'BT_S4'), (7, 8, 'TB_S6')):
        qc.h(a)
        qc.cx(a, b)
        qc.barrier(label=f'I2_{label}')
    coherent_teleportation(qc, 0, 3, 4, 'S2')
    append_encrypted_record_roundtrip(qc, 3, FIXED_KEY, '01', 'S3_X1_record')
    coherent_teleportation(qc, 4, 5, 6, 'S4')
    append_encrypted_record_roundtrip(qc, 5, FIXED_KEY, '10', 'S4_X2_record')
    qc.reset(0)
    _swap_test(qc, 0, 6, 2, 'S5_swap_test')
    coherent_teleportation(qc, 6, 7, 8, 'S6')
    append_encrypted_record_roundtrip(qc, 7, FIXED_KEY, '11', 'S6_X3_record')
    qc.reset(4)
    _swap_test(qc, 4, 8, 1, 'S7_swap_test')
    if input_state in {'+', '-'}:
        qc.h(8)
    qc.measure(0, 0)
    qc.measure(4, 1)
    qc.measure(8, 2)
    qc.metadata = {'candidate_type': 'faithful_v7_three_explicit_bell_resources', 'input_state': input_state,
'valid_expected_outputs': ['100'] if input_state in {'1', '-'} else ['000'], 'measurement_order': 'out[2] out[1] out[0]',
'success_definition': 'sum over valid expected outputs', 'paper_resource_qubits': 9, 'swap_ancillas': 'reuse of
consumed/dead measured-stage wires', 'not_implemented': ['QKD', 'authentication', 'network separation', 'abstract
arbitration']}
    return qc

def build_faithful_v7_unitary_reference(input_state='0'):
    qc = QuantumCircuit(11, name='faithful_v7_unitary_reference')
    _prepare_message_copies(qc, (0, 1, 2), input_state)
    for a, b in ((3, 4), (5, 6), (7, 8)):
        qc.h(a)
        qc.cx(a, b)
    coherent_teleportation(qc, 0, 3, 4, 'S2')
    append_encrypted_record_roundtrip(qc, 3, FIXED_KEY, '01', 'S3_X1_record')
    coherent_teleportation(qc, 4, 5, 6, 'S4')
    append_encrypted_record_roundtrip(qc, 5, FIXED_KEY, '10', 'S4_X2_record')
    _swap_test(qc, 9, 6, 2, 'S5_swap_test')
    coherent_teleportation(qc, 6, 7, 8, 'S6')
    append_encrypted_record_roundtrip(qc, 7, FIXED_KEY, '11', 'S6_X3_record')
    _swap_test(qc, 10, 8, 1, 'S7_swap_test')
    if input_state in {'+', '-'}:
        qc.h(8)
    return qc

def build_optimized_staged_candidate(input_state='0'):
    q = QuantumRegister(7, 'opt')
    c = ClassicalRegister(3, 'out')
    qc = QuantumCircuit(q, c, name='optimized_v7_staged_candidate')
    _prepare_message_copies(qc, (0, 1, 2), input_state)
    qc.barrier(label='S1')
```

```
    def leg(label):
        qc.h(3)
        qc.cx(3, 4)
        coherent_teleportation(qc, 0, 3, 4, label)
        qc.swap(0, 4)
        qc.reset(3)
        qc.reset(4)
    leg('S2')
    append_encrypted_record_roundtrip(qc, 6, FIXED_KEY, '01', 'S3_record')
    leg('S4')
    qc.reset(5)
    _swap_test(qc, 5, 0, 2, 'S5_swap_test')
    leg('S6')
    qc.reset(4)
    _swap_test(qc, 4, 0, 1, 'S7_swap_test')
    if input_state in {'+', '-'}:
        qc.h(0)
    qc.measure(5, 0)
    qc.measure(4, 1)
    qc.measure(0, 2)
    qc.metadata = {'candidate_type': 'optimized_staged_reset_reuse', 'input_state': input_state, 'valid_expected_outputs':
['100'] if input_state in {'1', '-'} else ['000'], 'measurement_order': 'out[2] out[1] out[0]', 'success_definition':
'sum over valid expected outputs', 'not_paper_resource_count': True}
    return qc
import csv
import matplotlib.pyplot as plt
from qiskit import qasm3, transpile
from qiskit.qasm3 import dumps as qasm3_dumps

# Self-contained matrix definitions used by the strengthened-QOTP circuit.
import numpy as np

X_MATRIX = np.array([[0, 1], [1, 0]], dtype=complex)
Y_MATRIX = np.array([[0, -1j], [1j, 0]], dtype=complex)
Z_MATRIX = np.array([[1, 0], [0, -1]], dtype=complex)
T_STRENGTHENED = np.sqrt(1j / 3) * (X_MATRIX - Y_MATRIX + Z_MATRIX)


def encryption_unitary(key4):
    """Return the one-qubit strengthened-QOTP unitary for a four-bit key block."""
    if len(key4) != 4 or set(key4) - {"0", "1"}:
        raise ValueError("key block must contain four binary digits")
    a, b, c, d = map(int, key4)
    return (
        np.linalg.matrix_power(X_MATRIX, a)
        @ np.linalg.matrix_power(Z_MATRIX, b)
        @ T_STRENGTHENED
        @ np.linalg.matrix_power(X_MATRIX, c)
        @ np.linalg.matrix_power(Z_MATRIX, d)
    )


# Circuit collection used for the modular and complete folders.
def build_swap_test_verification_circuit(left="0", right="1"):
    qc = QuantumCircuit(3, 1, name="swap_test_verification")
    if left == "1":
        qc.x(1)
    elif left == "+":
        qc.h(1)
    elif left == "-":
        qc.x(1); qc.h(1)
    if right == "1":
        qc.x(2)
    elif right == "+":
        qc.h(2)
    elif right == "-":
        qc.x(2); qc.h(2)
    qc.h(0); qc.cswap(0, 1, 2); qc.h(0); qc.measure(0, 0)
    return qc


def build_swap_test_circuit(left="0", right="1", name="swap_test"):
    """Compatibility wrapper used by the repeated swap-test circuit builder."""
    circuit = build_swap_test_verification_circuit(left, right)
    circuit.name = name
```

```python
    return circuit


def circuit_collection():
    staged = build_staged_components()
    modular = {
        "Bell_Pair": build_bell_pair_circuit(),
        "Three_Bell_Pairs": build_three_bell_pair_circuit(),
        "Teleportation": build_teleportation_circuit(),
        "Three_Teleportation_Rounds": build_three_teleportation_rounds(),
        "Strengthened_QOTP_Roundtrip": build_qotp_roundtrip_circuit(),
        "Encrypted_Bell_Record": build_encrypted_bell_record_circuit(),
        "Swap_Test_Verification": build_swap_test_verification_circuit(),
        "Stage_S2": staged["S2"],
        "Stage_S3_Record": staged["S3_record"],
        "Stage_S5_QOTP": staged["S5_QOTP"],
        "Stage_S6": staged["S6"],
    }
    complete = {
        "Composed_Full_Candidate": build_composed_staged_emulation_candidate(),
        "Optimized_Staged_Candidate": build_optimized_staged_candidate(),
    }
    return modular, complete


def save_circuit(circuit, base_path):
    base_path.parent.mkdir(parents=True, exist_ok=True)
    (base_path.with_suffix(".qasm")).write_text(qasm3_dumps(circuit), encoding="utf-8")
    (base_path.with_suffix(".txt")).write_text(str(circuit.draw("text", fold=160)), encoding="utf-8")
    figure = circuit.draw("mpl", style="iqp", fold=80, idle_wires=False)
    for extension in ("pdf", "png", "svg"):
        figure.savefig(base_path.with_suffix("." + extension), dpi=300 if extension == "png" else None,
bbox_inches="tight", facecolor="white")
    plt.close(figure)

def generate_logical_circuits():
    root = Path(__file__).resolve().parents[1] / "results"
    modular, complete = circuit_collection()
    for name, circuit in modular.items():
        save_circuit(circuit, root / "Modular_Circuits" / name)
    for name, circuit in complete.items():
        save_circuit(circuit, root / "Complete_Circuits" / name)


if __name__ == "__main__":
    generate_logical_circuits()
```

qiskit_protocol_reference.py

```
"""Complete IBM/Qiskit circuit, retrieval, evaluation, and guarded-submission code."""

from __future__ import annotations

import argparse
import csv
import hashlib
import json
from math import sqrt
from pathlib import Path

from qiskit import ClassicalRegister, QuantumCircuit, QuantumRegister, transpile
from qiskit.qasm3 import dumps as qasm3_dumps
from qiskit.transpiler import generate_preset_pass_manager
try:
    from qiskit_ibm_runtime import QiskitRuntimeService, SamplerV2
except ImportError:  # Optional for local BasicSimulator execution.
    QiskitRuntimeService = None
    SamplerV2 = None


# 1. Bell resources

def build_bell_pair_circuit(name='bell_pair'):
    q = QuantumRegister(2, 'bell')
    qc = QuantumCircuit(q, name=name)
    qc.h(q[0])
    qc.cx(q[0], q[1])
    return qc

def build_three_bell_pair_circuit():
    q = QuantumRegister(6, 'bell')
    qc = QuantumCircuit(q, name='three_v7_bell_pairs')
    for a, b in ((0, 1), (2, 3), (4, 5)):
        qc.h(q[a])
        qc.cx(q[a], q[b])
    qc.metadata = {'pairs': {'AB_S2': [0, 1], 'BT_S4': [2, 3], 'TB_S6': [4, 5]}}
    return qc

# 2. Teleportation

def coherent_teleportation(qc, message, bell_sender, bell_receiver, label):
    qc.barrier(label=f'{label}_Bell_measurement')
    qc.cx(message, bell_sender)
    qc.h(message)
    qc.cx(bell_sender, bell_receiver)
    qc.cz(message, bell_receiver)


SUPPORTED_INPUT_STATES = {"0", "1", "+", "-", "i+", "i-"}


def prepare_named_state(qc, qubit, input_state):
    """Prepare one of the named single-qubit validation states."""
    if input_state not in SUPPORTED_INPUT_STATES:
        raise ValueError(f"unsupported input state: {input_state}")
    if input_state == "1":
        qc.x(qubit)
    elif input_state == "+":
        qc.h(qubit)
    elif input_state == "-":
        qc.x(qubit)
        qc.h(qubit)
    elif input_state == "i+":
        qc.h(qubit)
        qc.s(qubit)
    elif input_state == "i-":
        qc.h(qubit)
        qc.sdg(qubit)


def decode_named_state_for_measurement(qc, qubit, input_state):
    """Rotate phase/Hadamard-basis validation states before Z measurement."""
```

```
    if input_state in {"+", "-"}:
        qc.h(qubit)
    elif input_state == "i+":
        qc.sdg(qubit)
        qc.h(qubit)
    elif input_state == "i-":
        qc.s(qubit)
        qc.h(qubit)


def expected_message_measurement_bit(input_state):
    if input_state not in SUPPORTED_INPUT_STATES:
        raise ValueError(f"unsupported input state: {input_state}")
    return "1" if input_state in {"1", "-"} else "0"


def composed_candidate_expected_outputs(input_state):
    """Return valid Qiskit keys in displayed order out[2] out[1] out[0]."""
    return frozenset({expected_message_measurement_bit(input_state) + "00"})


def normalize_qiskit_counts_key(key):
    """Remove classical-register separators without reversing bit order."""
    return "".join(str(key).split())


def success_probability_from_counts(counts, valid_outputs):
    """Sum counts for explicitly valid logical outputs; never use the mode."""
    expected = {normalize_qiskit_counts_key(value) for value in valid_outputs}
    shots = sum(int(value) for value in counts.values())
    if shots <= 0:
        raise ValueError("counts must contain at least one shot")
    successes = sum(
        int(value)
        for key, value in counts.items()
        if normalize_qiskit_counts_key(key) in expected
    )
    return successes / shots

def build_teleportation_circuit(input_state='0'):
    qc = QuantumCircuit(3, 1, name=f'teleport_{input_state}')
    prepare_named_state(qc, 0, input_state)
    qc.h(1)
    qc.cx(1, 2)
    coherent_teleportation(qc, 0, 1, 2, 'S2')
    decode_named_state_for_measurement(qc, 2, input_state)
    qc.measure(2, 0)
    return qc

def build_three_teleportation_rounds(input_state='0'):
    qc = QuantumCircuit(7, 1, name='three_teleportation_rounds')
    prepare_named_state(qc, 0, input_state)
    for a, b in ((1, 2), (3, 4), (5, 6)):
        qc.h(a)
        qc.cx(a, b)
    coherent_teleportation(qc, 0, 1, 2, 'S2')
    coherent_teleportation(qc, 2, 3, 4, 'S4')
    coherent_teleportation(qc, 4, 5, 6, 'S6')
    decode_named_state_for_measurement(qc, 6, input_state)
    qc.measure(6, 0)
    return qc

# 3. Strengthened-QOTP

def append_qotp(qc, qubit, key4, label='E_K'):
    U = encryption_unitary(key4)
    qc.unitary(U, [qubit], label=label)

def append_qotp_inverse(qc, qubit, key4, label='E_K^-1'):
    U = encryption_unitary(key4)
    qc.unitary(U.conj().T, [qubit], label=label)

def build_qotp_roundtrip_circuit(key4='0101', input_state='0'):
    qc = QuantumCircuit(1, 1, name='strengthened_qotp_roundtrip')
    if input_state == '1':
```

```
        qc.x(0)
    elif input_state == '+':
        qc.h(0)
    elif input_state == '-':
        qc.x(0)
        qc.h(0)
    append_qotp(qc, 0, key4)
    append_qotp_inverse(qc, 0, key4)
    if input_state in {'+', '-'}:
        qc.h(0)
    qc.measure(0, 0)
    return qc

# 4. Encrypted Bell records

def append_encrypted_record_roundtrip(qc, carrier, key4, record, label):
    if record[1] == '1':
        qc.x(carrier)
    if record[0] == '1':
        qc.z(carrier)
    append_qotp(qc, carrier, key4, f'{label}_encrypt')
    append_qotp_inverse(qc, carrier, key4, f'{label}_decrypt')
    if record[0] == '1':
        qc.z(carrier)
    if record[1] == '1':
        qc.x(carrier)

def build_encrypted_bell_record_circuit(key4='1010', record='01'):
    qc = QuantumCircuit(2, 2, name='encrypted_bell_record_roundtrip')
    qc.h(0)
    qc.cx(0, 1)
    append_encrypted_record_roundtrip(qc, 1, key4, record, 'E_KU_X')
    qc.cx(0, 1)
    qc.h(0)
    qc.measure([0, 1], [0, 1])
    return qc

# 5. Repeated swap tests

def build_repeated_swap_test_circuits(left='0', right='1', lam=8):
    return [build_swap_test_circuit(left, right, name=f'swap_{index + 1}_of_{lam}') for index in range(lam)]

# 6. Staged components

def build_staged_components():
    return {'S2': build_teleportation_circuit(), 'S3_record': build_encrypted_bell_record_circuit(), 'S5_QOTP':
build_qotp_roundtrip_circuit(), 'S6': build_teleportation_circuit()}

# 7. Composed staged-emulation candidate

FIXED_KEY = '0101'

def _prepare_message_copies(qc, qubits, input_state):
    for q in qubits:
        prepare_named_state(qc, q, input_state)

def _swap_test(qc, anc, left, right, label):
    qc.barrier(label=label)
    qc.h(anc)
    qc.cswap(anc, left, right)
    qc.h(anc)

def build_composed_staged_emulation_candidate(input_state='0'):
    q = QuantumRegister(9, 'candidate')
    c = ClassicalRegister(3, 'out')
    qc = QuantumCircuit(q, c, name='composed_staged_emulation_candidate')
    _prepare_message_copies(qc, (0, 1, 2), input_state)
    qc.barrier(label='S1_three_message_copies')
    for a, b, label in ((3, 4, 'AB_S2'), (5, 6, 'BT_S4'), (7, 8, 'TB_S6')):
        qc.h(a)
        qc.cx(a, b)
        qc.barrier(label=f'I2_{label}')
    coherent_teleportation(qc, 0, 3, 4, 'S2')
    append_encrypted_record_roundtrip(qc, 3, FIXED_KEY, '01', 'S3_X1_record')
    coherent_teleportation(qc, 4, 5, 6, 'S4')
```

```
    append_encrypted_record_roundtrip(qc, 5, FIXED_KEY, '10', 'S4_X2_record')
    qc.reset(0)
    _swap_test(qc, 0, 6, 2, 'S5_swap_test')
    coherent_teleportation(qc, 6, 7, 8, 'S6')
    append_encrypted_record_roundtrip(qc, 7, FIXED_KEY, '11', 'S6_X3_record')
    qc.reset(4)
    _swap_test(qc, 4, 8, 1, 'S7_swap_test')
    decode_named_state_for_measurement(qc, 8, input_state)
    qc.measure(0, 0)
    qc.measure(4, 1)
    qc.measure(8, 2)
    valid_outputs = sorted(composed_candidate_expected_outputs(input_state))
    qc.metadata = {'candidate_type': 'faithful_v7_three_explicit_bell_resources', 'input_state': input_state,
'valid_expected_outputs': valid_outputs, 'measurement_order': 'out[2] out[1] out[0]', 'success_definition': 'sum of
counts over valid_expected_outputs', 'paper_resource_qubits': 9, 'swap_ancillas': 'reuse of consumed/dead measured-stage
wires', 'not_implemented': ['QKD', 'authentication', 'network separation', 'abstract arbitration']}
    return qc

def build_faithful_v7_unitary_reference(input_state='0'):
    qc = QuantumCircuit(11, name='faithful_v7_unitary_reference')
    _prepare_message_copies(qc, (0, 1, 2), input_state)
    for a, b in ((3, 4), (5, 6), (7, 8)):
        qc.h(a)
        qc.cx(a, b)
    coherent_teleportation(qc, 0, 3, 4, 'S2')
    append_encrypted_record_roundtrip(qc, 3, FIXED_KEY, '01', 'S3_X1_record')
    coherent_teleportation(qc, 4, 5, 6, 'S4')
    append_encrypted_record_roundtrip(qc, 5, FIXED_KEY, '10', 'S4_X2_record')
    _swap_test(qc, 9, 6, 2, 'S5_swap_test')
    coherent_teleportation(qc, 6, 7, 8, 'S6')
    append_encrypted_record_roundtrip(qc, 7, FIXED_KEY, '11', 'S6_X3_record')
    _swap_test(qc, 10, 8, 1, 'S7_swap_test')
    decode_named_state_for_measurement(qc, 8, input_state)
    return qc

def build_optimized_staged_candidate(input_state='0'):
    q = QuantumRegister(7, 'opt')
    c = ClassicalRegister(3, 'out')
    qc = QuantumCircuit(q, c, name='optimized_v7_staged_candidate')
    _prepare_message_copies(qc, (0, 1, 2), input_state)
    qc.barrier(label='S1')

    def leg(label):
        qc.h(3)
        qc.cx(3, 4)
        coherent_teleportation(qc, 0, 3, 4, label)
        qc.swap(0, 4)
        qc.reset(3)
        qc.reset(4)
    leg('S2')
    append_encrypted_record_roundtrip(qc, 6, FIXED_KEY, '01', 'S3_record')
    leg('S4')
    qc.reset(5)
    _swap_test(qc, 5, 0, 2, 'S5_swap_test')
    leg('S6')
    qc.reset(4)
    _swap_test(qc, 4, 0, 1, 'S7_swap_test')
    decode_named_state_for_measurement(qc, 0, input_state)
    qc.measure(5, 0)
    qc.measure(4, 1)
    qc.measure(0, 2)
    valid_outputs = sorted(composed_candidate_expected_outputs(input_state))
    qc.metadata = {'candidate_type': 'optimized_staged_reset_reuse', 'input_state': input_state, 'valid_expected_outputs':
valid_outputs, 'measurement_order': 'out[2] out[1] out[0]', 'success_definition': 'sum of counts over
valid_expected_outputs', 'not_paper_resource_count': True}
    return qc
import csv
import matplotlib.pyplot as plt
from qiskit import qasm3, transpile
from qiskit.qasm3 import dumps as qasm3_dumps

# Self-contained matrix definitions used by the strengthened-QOTP circuit.
import numpy as np

X_MATRIX = np.array([[0, 1], [1, 0]], dtype=complex)
```

```python
Y_MATRIX = np.array([[0, -1j], [1j, 0]], dtype=complex)
Z_MATRIX = np.array([[1, 0], [0, -1]], dtype=complex)
T_STRENGTHENED = np.sqrt(1j / 3) * (X_MATRIX - Y_MATRIX + Z_MATRIX)


def encryption_unitary(key4):
    """Return the one-qubit strengthened-QOTP unitary for a four-bit key block."""
    if len(key4) != 4 or set(key4) - {"0", "1"}:
        raise ValueError("key block must contain four binary digits")
    a, b, c, d = map(int, key4)
    return (
        np.linalg.matrix_power(X_MATRIX, a)
        @ np.linalg.matrix_power(Z_MATRIX, b)
        @ T_STRENGTHENED
        @ np.linalg.matrix_power(X_MATRIX, c)
        @ np.linalg.matrix_power(Z_MATRIX, d)
    )


# Circuit collection used for the modular and complete folders.
def build_swap_test_verification_circuit(left="0", right="1"):
    qc = QuantumCircuit(3, 1, name="swap_test_verification")
    if left == "1":
        qc.x(1)
    elif left == "+":
        qc.h(1)
    elif left == "-":
        qc.x(1); qc.h(1)
    if right == "1":
        qc.x(2)
    elif right == "+":
        qc.h(2)
    elif right == "-":
        qc.x(2); qc.h(2)
    qc.h(0); qc.cswap(0, 1, 2); qc.h(0); qc.measure(0, 0)
    return qc


def build_swap_test_circuit(left="0", right="1", name="swap_test"):
    """Compatibility wrapper used by the repeated swap-test circuit builder."""
    circuit = build_swap_test_verification_circuit(left, right)
    circuit.name = name
    return circuit


def circuit_collection():
    staged = build_staged_components()
    modular = {
        "Bell_Pair": build_bell_pair_circuit(),
        "Three_Bell_Pairs": build_three_bell_pair_circuit(),
        "Teleportation": build_teleportation_circuit(),
        "Three_Teleportation_Rounds": build_three_teleportation_rounds(),
        "Strengthened_QOTP_Roundtrip": build_qotp_roundtrip_circuit(),
        "Encrypted_Bell_Record": build_encrypted_bell_record_circuit(),
        "Swap_Test_Verification": build_swap_test_verification_circuit(),
        "Stage_S2": staged["S2"],
        "Stage_S3_Record": staged["S3_record"],
        "Stage_S5_QOTP": staged["S5_QOTP"],
        "Stage_S6": staged["S6"],
    }
    complete = {
        "Composed_Full_Candidate": build_composed_staged_emulation_candidate(),
        "Optimized_Staged_Candidate": build_optimized_staged_candidate(),
    }
    return modular, complete


def save_circuit(circuit, base_path):
    base_path.parent.mkdir(parents=True, exist_ok=True)
    (base_path.with_suffix(".qasm")).write_text(qasm3_dumps(circuit), encoding="utf-8")
    (base_path.with_suffix(".txt")).write_text(str(circuit.draw("text", fold=160)), encoding="utf-8")
    figure = circuit.draw("mpl", style="iqp", fold=80, idle_wires=False)
    for extension in ("pdf", "png", "svg"):
        figure.savefig(base_path.with_suffix("." + extension), dpi=300 if extension == "png" else None,
bbox_inches="tight", facecolor="white")
```

```python
    plt.close(figure)

def generate_logical_circuits():
    root = Path(__file__).resolve().parent
    modular, complete = circuit_collection()
    for name, circuit in modular.items():
        save_circuit(circuit, root / "Modular_Circuits" / name)
    for name, circuit in complete.items():
        save_circuit(circuit, root / "Complete_Circuits" / name)


if False and __name__ == "__main__":
    generate_logical_circuits()


# Qiskit reference execution and result plotting
from qiskit.providers.basic_provider import BasicSimulator


def _measured_copy(circuit):
    measured = circuit.copy()
    if measured.num_clbits == 0:
        measured.measure_all()
    return measured


def run_qiskit_reference_data(output_csv: Path, shots: int = 1024, seed: int = 20260803) -> None:
    """Run modular and complete circuits with Qiskit's basic reference simulator."""
    backend = BasicSimulator()
    modular, complete = circuit_collection()
    rows = []
    for group, circuits in (("modular", modular), ("complete", complete)):
        for name, logical in circuits.items():
            measured = _measured_copy(logical)
            executable = transpile(measured, backend=backend, optimization_level=0, seed_transpiler=seed)
            counts = backend.run(executable, shots=shots, seed_simulator=seed).result().get_counts()
            valid_outputs = logical.metadata.get("valid_expected_outputs") if logical.metadata else None
            if valid_outputs is None:
                valid_outputs = {"0" * measured.num_clbits}
            rows.append({
                "group": group, "circuit": name, "shots": shots,
                "depth": executable.depth(), "size": executable.size(),
                "valid_expected_outputs": json.dumps(sorted(valid_outputs)),
                "correct_output_probability": success_probability_from_counts(counts, valid_outputs),
                "counts_json": json.dumps(counts, sort_keys=True),
            })
    output_csv.parent.mkdir(parents=True, exist_ok=True)
    with output_csv.open("w", newline="", encoding="utf-8") as handle:
        writer = csv.DictWriter(handle, fieldnames=list(rows[0])); writer.writeheader(); writer.writerows(rows)


def generate_qiskit_result_figure(input_csv: Path, output_path: Path) -> None:
    rows = list(csv.DictReader(input_csv.open(encoding="utf-8")))
    fig, ax = plt.subplots(figsize=(9.0, 4.8))
    ax.bar(range(len(rows)), [float(row["correct_output_probability"]) for row in rows], color="0.35")
    ax.set(xticks=range(len(rows)), xticklabels=[row["circuit"] for row in rows],
           ylabel="Correct-output probability", ylim=(0, 1.05), title="Qiskit reference execution")
    ax.tick_params(axis="x", rotation=65); fig.tight_layout()
    output_path.parent.mkdir(parents=True, exist_ok=True)
    fig.savefig(output_path.with_suffix(".png"), dpi=300); fig.savefig(output_path.with_suffix(".pdf")); plt.close(fig)


def run_complete_qiskit_pipeline(output_dir: Path, shots: int = 1024) -> None:
    generate_logical_circuits()
    data = output_dir / "qiskit_reference_results.csv"
    run_qiskit_reference_data(data, shots)
    generate_qiskit_result_figure(data, output_dir / "qiskit_reference_results")


if __name__ == "__main__":
    parser = argparse.ArgumentParser(description="Generate Qiskit circuits, data, and result figures.")
    parser.add_argument("--output-dir", type=Path, default=Path("qiskit_results"))
    parser.add_argument("--shots", type=int, default=1024)
    arguments = parser.parse_args(); run_complete_qiskit_pipeline(arguments.output_dir, arguments.shots)
```

resource_operation_ledger.py

```
"""Step-derived resource accounting for one physical-copy protocol batch."""

from __future__ import annotations

from dataclasses import asdict, dataclass


EXECUTION_MODEL = (
    "one protocol execution with 2*lambda+1 p1 copies, lambda p2 copies, "
    "lambda p3 copies, and random S5/S7 subset verification"
)
REQUIRES_CONFIRMATION = "REQUIRES_MANUSCRIPT_CONFIRMATION"


@dataclass(frozen=True)
class EncryptedObjectSpec:
    encryption_step: str
    recovery_step: str
    purpose: str
    copy_id: str
    bit_length: int


@dataclass(frozen=True)
class StepContribution:
    step: str
    rationale: str
    message_state_preparations: int = 0
    message_qubit_instances: int = 0
    bell_pairs: int = 0
    bell_qubits: int = 0
    fresh_qotp_key_bits: int = 0
    teleportation_operations: int = 0
    controlled_swaps: int = 0
    teleportation_measurements: int = 0
    swap_measurements: int = 0
    message_preparation_operations: int = 0
    bell_pair_preparation_gates: int = 0
    qotp_unitary_applications: int = 0

    @property
    def measurements(self) -> int:
        return self.teleportation_measurements + self.swap_measurements

    @property
    def modeled_operations(self) -> int:
        return (
            self.message_preparation_operations
            + self.bell_pair_preparation_gates
            + self.teleportation_operations
            + self.controlled_swaps
            + self.measurements
            + self.qotp_unitary_applications
        )


def _validate_dimensions(n: int, lambda_parameter: int) -> tuple[int, int]:
    if isinstance(n, bool) or not isinstance(n, int) or n < 1:
        raise ValueError("n must be a positive integer")
    if isinstance(lambda_parameter, bool) or not isinstance(lambda_parameter, int) or lambda_parameter < 1:
        raise ValueError("lambda_parameter must be a positive integer")
    return n, lambda_parameter


def physical_copy_counts(lambda_parameter: int) -> dict[str, int]:
    _, lam = _validate_dimensions(1, lambda_parameter)
    return {"p1": 2 * lam + 1, "p2": lam, "p3": lam, "total": 4 * lam + 1}


def encrypted_object_ledger(lambda_parameter: int) -> list[EncryptedObjectSpec]:
    """Return every single-qubit encrypted-object key block in one batch.

    A block is independently allocated; sampled bit strings are not required to
```

```
    be pairwise different.
    """
    counts = physical_copy_counts(lambda_parameter)
    rows: list[EncryptedObjectSpec] = []
    rows.extend(EncryptedObjectSpec("S3", "S5", "p3_key", f"p3[{j}]", 4) for j in range(counts["p3"]))
    for i in range(counts["p1"]):
        rows.append(EncryptedObjectSpec("S3", "S5", "s3_bell_key", f"p1[{i}]", 4))
        rows.append(EncryptedObjectSpec("S3", "S5", "x1_record_key", f"p1[{i}]", 8))
        rows.append(EncryptedObjectSpec("S4", "S5", "s4_bell_key", f"p1[{i}]", 4))
        rows.append(EncryptedObjectSpec("S4", "S5", "x2_record_key", f"p1[{i}]", 8))
    for j in range(counts["p1"] - counts["p3"]):
        rows.append(EncryptedObjectSpec("S6", "S7", "s6_bell_key", f"returned_p1[{j}]", 4))
        rows.append(EncryptedObjectSpec("S6", "S7", "x3_record_key", f"returned_p1[{j}]", 8))
    return rows


def fresh_key_bits_per_message_qubit(lambda_parameter: int) -> int:
    return sum(item.bit_length for item in encrypted_object_ledger(lambda_parameter))


def protocol_step_ledger(n: int, lambda_parameter: int) -> tuple[StepContribution, ...]:
    """Derive resource contributions from the physical-copy protocol steps."""
    n, lam = _validate_dimensions(n, lambda_parameter)
    copies = physical_copy_counts(lam)
    objects = encrypted_object_ledger(lam)

    def key_bits(step: str) -> int:
        return n * sum(item.bit_length for item in objects if item.encryption_step == step)

    def encryption_count(step: str) -> int:
        return n * sum(1 for item in objects if item.encryption_step == step)

    def recovery_count(step: str) -> int:
        return n * sum(1 for item in objects if item.recovery_step == step)

    return (
        StepContribution(
            "S1",
            "Prepare all p1, p2, and p3 physical message copies.",
            message_state_preparations=copies["total"],
            message_qubit_instances=copies["total"] * n,
            message_preparation_operations=copies["total"] * n,
        ),
        StepContribution(
            "S2",
            "Teleport every one of the 2*lambda+1 p1 copies from Alice to Bob.",
            bell_pairs=copies["p1"] * n,
            bell_qubits=2 * copies["p1"] * n,
            teleportation_operations=copies["p1"] * n,
            teleportation_measurements=2 * copies["p1"] * n,
            bell_pair_preparation_gates=2 * copies["p1"] * n,
        ),
        StepContribution(
            "S3",
            "Encrypt p3 references, S3 Bell-path objects, and X1 records with fresh key blocks.",
            fresh_qotp_key_bits=key_bits("S3"),
            qotp_unitary_applications=encryption_count("S3"),
        ),
        StepContribution(
            "S4",
            "Teleport all received p1 copies from Bob to Trent and allocate fresh S4/X2 key blocks.",
            bell_pairs=copies["p1"] * n,
            bell_qubits=2 * copies["p1"] * n,
            fresh_qotp_key_bits=key_bits("S4"),
            teleportation_operations=copies["p1"] * n,
            teleportation_measurements=2 * copies["p1"] * n,
            bell_pair_preparation_gates=2 * copies["p1"] * n,
            qotp_unitary_applications=encryption_count("S4"),
        ),
        StepContribution(
            "S5",
            "Recover/check p3, X1, and X2 protected objects; sample lambda recovered p1 copies without replacement and compare them with p3 references.",
            controlled_swaps=lam * n,
            swap_measurements=lam,
```

```
            qotp_unitary_applications=recovery_count("S5"),
        ),
        StepContribution(
            "S6",
            "Teleport the lambda+1 remaining p1 copies from Trent to Bob and allocate fresh S6/X3 key blocks.",
            bell_pairs=(lam + 1) * n,
            bell_qubits=2 * (lam + 1) * n,
            fresh_qotp_key_bits=key_bits("S6"),
            teleportation_operations=(lam + 1) * n,
            teleportation_measurements=2 * (lam + 1) * n,
            bell_pair_preparation_gates=2 * (lam + 1) * n,
            qotp_unitary_applications=encryption_count("S6"),
        ),
        StepContribution(
            "S7",
            "Recover/check X3 protected objects, sample lambda returned p1 copies without replacement, compare with p2,
and retain the unique unsampled copy.",
            controlled_swaps=lam * n,
            swap_measurements=lam,
            qotp_unitary_applications=recovery_count("S7"),
        ),
    )


def step_rows(n: int, lambda_parameter: int) -> list[dict]:
    rows = []
    for item in protocol_step_ledger(n, lambda_parameter):
        row = asdict(item)
        row["n"] = n
        row["lambda_parameter"] = lambda_parameter
        row["measurements"] = item.measurements
        row["modeled_operations_for_step"] = item.modeled_operations
        rows.append(row)
    return rows


def summarize_resources(n: int, lambda_parameter: int) -> dict:
    n, lam = _validate_dimensions(n, lambda_parameter)
    steps = protocol_step_ledger(n, lam)
    copies = physical_copy_counts(lam)

    def total(field: str) -> int:
        return sum(getattr(item, field) for item in steps)

    key_bits = total("fresh_qotp_key_bits")
    expected_key_bits = fresh_key_bits_per_message_qubit(lam) * n
    if key_bits != expected_key_bits:
        raise RuntimeError("step ledger and encrypted-object key ledger disagree")

    return {
        "n": n,
        "lambda_parameter": lam,
        "execution_model": EXECUTION_MODEL,
        "p1_physical_copies": copies["p1"],
        "p2_physical_copies": copies["p2"],
        "p3_physical_copies": copies["p3"],
        "total_message_state_preparations": total("message_state_preparations"),
        "message_physical_qubit_instances": total("message_qubit_instances"),
        "s2_bell_pairs": copies["p1"] * n,
        "s4_bell_pairs": copies["p1"] * n,
        "s6_bell_pairs": (lam + 1) * n,
        "total_bell_pairs": total("bell_pairs"),
        "total_bell_qubits": total("bell_qubits"),
        "fresh_qotp_key_bits": key_bits,
        "quantum_transmitted_qubits": REQUIRES_CONFIRMATION,
        "classical_transmitted_bits": REQUIRES_CONFIRMATION,
        "teleportation_operations": total("teleportation_operations"),
        "s5_controlled_swap_gates": lam * n,
        "s7_controlled_swap_gates": lam * n,
        "total_controlled_swap_gates": total("controlled_swaps"),
        "teleportation_measurements": total("teleportation_measurements"),
        "swap_test_measurements": total("swap_measurements"),
        "total_measurements": total("teleportation_measurements") + total("swap_measurements"),
        "bell_pair_preparation_gates": total("bell_pair_preparation_gates"),
        "qotp_unitary_applications": total("qotp_unitary_applications"),
```

```
        "total_modeled_operations": sum(item.modeled_operations for item in steps),
        "peak_live_qubits": REQUIRES_CONFIRMATION,
        "fixed_lambda_scaling_in_n": "O(n)",
        "joint_lambda_n_scaling": "O(lambda*n)",
    }


def resource_scaling_rows(n_values=range(1, 21), lambda_values=(1, 2, 4, 8)) -> list[dict]:
    return [summarize_resources(n, lam) for n in n_values for lam in lambda_values]
```

verification_experiment_suite.py

```
"""Verification experiment suite for local numerical, Qiskit, Aer, data, and figure workflows.

This organized copy never connects to IBM Quantum and contains no submission path.
Protocol-level simulation uses one physical-copy batch parameterized by ``lambda``.
The generic repeated Swap-Test helper retains ``m`` for the number of independent
comparisons and accepts ``lambda_repetitions`` only as a compatibility alias.
"""

from __future__ import annotations

import argparse
import csv
import hashlib
import json
import math
import platform
import sys
from dataclasses import dataclass, field
from datetime import datetime, timezone
from pathlib import Path

import matplotlib

matplotlib.use("Agg")
import matplotlib.pyplot as plt
from matplotlib.ticker import StrMethodFormatter
import numpy as np
import pandas as pd
from qiskit import ClassicalRegister, QuantumCircuit, QuantumRegister, transpile
from qiskit.providers.basic_provider import BasicSimulator
from qiskit.quantum_info import Statevector
from qiskit_aer import AerSimulator


SEED = 20260903
SWAP_TRIALS = 50_000
PROTOCOL_TRIALS = 4_000
AER_SHOTS = 16_384
FIDELITIES = (0.0, 0.25, 0.5, 0.75, 0.9, 0.99)
M_VALUES = (1, 2, 4, 8, 16, 32, 40)
PROTOCOL_LAMBDA_VALUES = (1, 2, 4, 8)
NOISE_VALUES = (0.0, 0.02, 0.05)

I2 = np.eye(2, dtype=complex)
X = np.array([[0, 1], [1, 0]], dtype=complex)
Y = np.array([[0, -1j], [1j, 0]], dtype=complex)
Z = np.array([[1, 0], [0, -1]], dtype=complex)
PLUS = np.array([1, 1], dtype=complex) / np.sqrt(2)
T_STRENGTHENED = np.sqrt(1j / 3) * (X - Y + Z)


def write_rows(path: Path, rows: list[dict]) -> None:
    path.parent.mkdir(parents=True, exist_ok=True)
    with path.open("w", newline="", encoding="utf-8") as handle:
        writer = csv.DictWriter(handle, fieldnames=list(rows[0]))
        writer.writeheader()
        writer.writerows(rows)


def sha256(path: Path) -> str:
    digest = hashlib.sha256()
    with path.open("rb") as handle:
        for block in iter(lambda: handle.read(1024 * 1024), b""):
            digest.update(block)
    return digest.hexdigest()


def normalized(vector: np.ndarray) -> np.ndarray:
    value = np.asarray(vector, dtype=complex)
    norm = float(np.linalg.norm(value))
    if not np.isfinite(norm) or norm <= 1e-15:
        raise ValueError("state vector must have a finite, non-zero norm")
    return value / norm
```

```python
def fidelity(left: np.ndarray, right: np.ndarray) -> float:
    value = float(abs(np.vdot(normalized(left), normalized(right))) ** 2)
    if not np.isfinite(value):
        raise ValueError("fidelity must be finite")
    return float(np.clip(value, 0.0, 1.0))


def swap_accept_single(F: float) -> float:
    if not -1e-12 <= F <= 1.0 + 1e-12:
        raise ValueError("fidelity must be in [0, 1]")
    F = min(1.0, max(0.0, float(F)))
    return (1.0 + F) / 2.0


def swap_accept_all(fidelities: list[float] | tuple[float, ...] | np.ndarray) -> float:
    """General independent-instance model: product_j ((1 + F_j) / 2)."""
    values = np.asarray(fidelities, dtype=float)
    if values.ndim != 1 or values.size == 0 or np.any(values < -1e-12) or np.any(values > 1 + 1e-12):
        raise ValueError("fidelities must be a non-empty one-dimensional sequence in [0, 1]")
    values = np.clip(values, 0.0, 1.0)
    return float(np.prod((1.0 + values) / 2.0))


def swap_detection(fidelities: list[float] | tuple[float, ...] | np.ndarray) -> float:
    return 1.0 - swap_accept_all(fidelities)


def repeated_swap_accept_probability(
    F: float,
    m: int | None = None,
    *,
    lambda_repetitions: int | None = None,
) -> float:
    """Compatibility wrapper; ``lambda_repetitions`` is converted to ``m``."""
    if m is None:
        if lambda_repetitions is None:
            raise TypeError("m is required")
        m = lambda_repetitions
    elif lambda_repetitions is not None and m != lambda_repetitions:
        raise ValueError("conflicting m and lambda_repetitions values")
    if int(m) != m or m < 1:
        raise ValueError("m must be a positive integer")
    return swap_accept_all([F] * int(m))


def generate_swap_data(path: Path) -> float:
    rng = np.random.default_rng(SEED)
    rows = []
    for F in FIDELITIES:
        p_single = swap_accept_single(F)
        for m in M_VALUES:
            # Every Monte Carlo trial draws m separate Bernoulli outcomes.
            outcomes = rng.random((SWAP_TRIALS, m)) < p_single
            mc_accept = float(np.mean(np.all(outcomes, axis=1)))
            analytical_accept = swap_accept_all([F] * m)
            rows.append(
                {
                    "fidelity": F,
                    "m": m,
                    "trials": SWAP_TRIALS,
                    "analytical_accept_probability": analytical_accept,
                    "analytical_detection_probability": 1.0 - analytical_accept,
                    "monte_carlo_accept_probability": mc_accept,
                    "monte_carlo_detection_probability": 1.0 - mc_accept,
                    "absolute_error_accept": abs(mc_accept - analytical_accept),
                    "absolute_error_detection": abs(mc_accept - analytical_accept),
                }
            )
    write_rows(path, rows)
    return max(row["absolute_error_accept"] for row in rows)


def encryption_unitary(key4: str) -> np.ndarray:
```

```python
    a, b, c, d = map(int, key4)
    return (
        np.linalg.matrix_power(X, a)
        @ np.linalg.matrix_power(Z, b)
        @ T_STRENGTHENED
        @ np.linalg.matrix_power(X, c)
        @ np.linalg.matrix_power(Z, d)
    )


@dataclass
class StageComparison:
    stage: str
    p1_index: int
    reference_index: int
    fidelity: float
    effective_fidelity: float
    accept_probability: float
    outcome_accept: bool
    records_ok: bool


@dataclass
class FreshKeyBlock:
    block_id: str
    purpose: str
    copy_id: str
    bits: str
    consumed: bool = False
    consumption_count: int = 0


class FreshKeyPool:
    """Deterministic simulated QKD pool with single-consumption block IDs."""

    def __init__(self, rng: np.random.Generator):
        self._rng = rng
        self._counter = 0
        self.blocks: dict[str, FreshKeyBlock] = {}

    def allocate(self, length: int, purpose: str, copy_id: str) -> FreshKeyBlock:
        if isinstance(length, bool) or not isinstance(length, int) or length < 1:
            raise ValueError("key length must be a positive integer")
        self._counter += 1
        block_id = f"key-{self._counter:05d}"
        block = FreshKeyBlock(block_id, purpose, copy_id, random_bits(self._rng, length))
        self.blocks[block_id] = block
        return block

    def consume(self, block_id: str) -> FreshKeyBlock:
        block = self.blocks[block_id]
        if block.consumed:
            raise RuntimeError(f"fresh key block reused: {block_id}")
        block.consumed = True
        block.consumption_count += 1
        return block


@dataclass
class ProtocolBatchState:
    lambda_parameter: int
    p1_copies: list[np.ndarray]
    p2_copies: list[np.ndarray]
    p3_copies: list[np.ndarray]
    key_pool: FreshKeyPool
    encrypted_object_key_blocks: dict[str, str]
    x1_records: list[str]
    x2_records: list[str]
    x3_records: list[str]
    s2_outputs: list[np.ndarray] = field(default_factory=list)
    s4_outputs: list[np.ndarray] = field(default_factory=list)
    recovered_at_trent: list[np.ndarray] = field(default_factory=list)
    s5_test_indices: list[int] = field(default_factory=list)
    s5_remaining_indices: list[int] = field(default_factory=list)
    s6_outputs: list[np.ndarray] = field(default_factory=list)
```

```python
    s7_test_indices: list[int] = field(default_factory=list)
    retained_p1_index: int | None = None
    s5_comparisons: list[StageComparison] = field(default_factory=list)
    s7_comparisons: list[StageComparison] = field(default_factory=list)
    trent_accept: bool = False
    bob_accept: bool = False
    final_accept: bool = False
    attacked_index: int | str | None = None
    inputs_committed_before_sampling: bool = False
    protocol_state: dict = field(default_factory=dict)


def random_bits(rng: np.random.Generator, count: int) -> str:
    return "".join(str(int(value)) for value in rng.integers(0, 2, size=count))


def prepare_protocol_batch(lambda_parameter: int, rng: np.random.Generator) -> ProtocolBatchState:
    if isinstance(lambda_parameter, bool) or not isinstance(lambda_parameter, int) or lambda_parameter < 1:
        raise ValueError("lambda_parameter must be a positive integer")
    p1_count = 2 * lambda_parameter + 1
    p2_count = p3_count = lambda_parameter
    pool = FreshKeyPool(rng)
    bindings: dict[str, str] = {}

    from resource_operation_ledger import encrypted_object_ledger

    for item in encrypted_object_ledger(lambda_parameter):
        block = pool.allocate(item.bit_length, item.purpose, item.copy_id)
        pool.consume(block.block_id)
        bindings[f"{item.purpose}:{item.copy_id}"] = block.block_id

    batch = ProtocolBatchState(
        lambda_parameter=lambda_parameter,
        p1_copies=[PLUS.copy() for _ in range(p1_count)],
        p2_copies=[PLUS.copy() for _ in range(p2_count)],
        p3_copies=[PLUS.copy() for _ in range(p3_count)],
        key_pool=pool,
        encrypted_object_key_blocks=bindings,
        x1_records=[random_bits(rng, 2) for _ in range(p1_count)],
        x2_records=[random_bits(rng, 2) for _ in range(p1_count)],
        x3_records=[random_bits(rng, 2) for _ in range(lambda_parameter + 1)],
    )
    # Teleportation is ideal in this state-level model; each output is still a
    # distinct physical state allocation and each path has its own record.
    batch.s2_outputs = [state.copy() for state in batch.p1_copies]
    batch.s4_outputs = [state.copy() for state in batch.s2_outputs]
    batch.recovered_at_trent = [state.copy() for state in batch.s4_outputs]
    batch.protocol_state = {
        "all_states_received": True,
        "all_encrypted_records_received": True,
        "sampling_authority_s5": "Trent",
        "sampling_authority_s7": "Bob",
    }
    batch.inputs_committed_before_sampling = True
    return batch


def effective_fidelity(F: float, noise: float) -> float:
    """Phenomenological state-level replacement model, not a hardware noise model."""
    if not 0.0 <= noise <= 1.0:
        raise ValueError("noise must be in [0, 1]")
    return float(np.clip((1.0 - noise) * F + noise / 2.0, 0.0, 1.0))


def _sample_comparison(
    stage: str,
    p1_index: int,
    reference_index: int,
    left: np.ndarray,
    right: np.ndarray,
    records_ok: bool,
    noise: float,
    rng: np.random.Generator,
) -> StageComparison:
    ideal_F = fidelity(left, right)
```

```python
    noisy_F = effective_fidelity(ideal_F, noise)
    probability = swap_accept_single(noisy_F)
    return StageComparison(
        stage=stage,
        p1_index=p1_index,
        reference_index=reference_index,
        fidelity=ideal_F,
        effective_fidelity=noisy_F,
        accept_probability=probability,
        outcome_accept=bool(rng.random() < probability),
        records_ok=records_ok,
    )


def execute_protocol_batch(
    batch: ProtocolBatchState,
    scenario: str,
    noise: float,
    rng: np.random.Generator,
) -> bool:
    """Execute one physical-copy batch with explicit S5/S7 comparisons."""
    if not batch.inputs_committed_before_sampling:
        raise RuntimeError("S5 sampling requires all states and records to be committed")
    lam = batch.lambda_parameter
    recovered_s5 = [state.copy() for state in batch.recovered_at_trent]
    reference_s5 = [state.copy() for state in batch.p3_copies]
    reference_s7 = [state.copy() for state in batch.p2_copies]
    s5_records_ok = True
    s7_records_ok = True

    if scenario == "alice_scenario_1":
        # Consistency-only diagnostic: every physical copy is changed coherently.
        changed = Z @ PLUS
        recovered_s5 = [changed.copy() for _ in recovered_s5]
        reference_s5 = [changed.copy() for _ in reference_s5]
        reference_s7 = [changed.copy() for _ in reference_s7]
    elif scenario == "tamper_p2":
        reference_s7 = [Z @ state for state in reference_s7]
        batch.attacked_index = "ALL_P2"
    elif scenario == "tamper_p3":
        tampered_references = []
        for j, state in enumerate(reference_s5):
            block_id = batch.encrypted_object_key_blocks[f"p3_key:p3[{j}]"]
            unitary = encryption_unitary(batch.key_pool.blocks[block_id].bits)
            tampered_references.append(unitary.conj().T @ X @ unitary @ state)
        reference_s5 = tampered_references
        batch.attacked_index = "ALL_P3"
    elif scenario == "impersonation_attempt":
        replacement_ciphertext = np.array([1, 0], dtype=complex)
        replacements = []
        for j in range(lam):
            block_id = batch.encrypted_object_key_blocks[f"p3_key:p3[{j}]"]
            unitary = encryption_unitary(batch.key_pool.blocks[block_id].bits)
            replacements.append(unitary.conj().T @ replacement_ciphertext)
        reference_s5 = replacements
        batch.attacked_index = "ALL_P3"
    elif scenario == "bob_tampering_s6_record":
        s7_records_ok = False
        batch.attacked_index = 0
    elif scenario == "replay_record_mismatch":
        s5_records_ok = False
        batch.attacked_index = 0
    elif scenario != "honest":
        raise ValueError(f"unknown scenario: {scenario}")

    all_indices = list(range(2 * lam + 1))
    batch.s5_test_indices = sorted(int(i) for i in rng.choice(all_indices, size=lam, replace=False))
    batch.s5_remaining_indices = [i for i in all_indices if i not in set(batch.s5_test_indices)]
    batch.s5_comparisons = [
        _sample_comparison(
            "S5", p1_index, reference_index, recovered_s5[p1_index], reference_s5[reference_index],
            s5_records_ok, noise, rng,
        )
        for reference_index, p1_index in enumerate(batch.s5_test_indices)
    ]
```

```
    batch.trent_accept = bool(all(item.records_ok and item.outcome_accept for item in batch.s5_comparisons))
    if not batch.trent_accept:
        return False

    batch.s6_outputs = [recovered_s5[i].copy() for i in batch.s5_remaining_indices]
    s7_positions = sorted(int(i) for i in rng.choice(range(lam + 1), size=lam, replace=False))
    batch.s7_test_indices = [batch.s5_remaining_indices[position] for position in s7_positions]
    retained = [i for i in batch.s5_remaining_indices if i not in set(batch.s7_test_indices)]
    if len(retained) != 1:
        raise RuntimeError("S7 must retain exactly one p1 copy")
    batch.retained_p1_index = retained[0]
    returned_by_original_index = dict(zip(batch.s5_remaining_indices, batch.s6_outputs))
    batch.s7_comparisons = [
        _sample_comparison(
            "S7", p1_index, reference_index, returned_by_original_index[p1_index], reference_s7[reference_index],
            s7_records_ok, noise, rng,
        )
        for reference_index, p1_index in enumerate(batch.s7_test_indices)
    ]
    batch.bob_accept = bool(all(item.records_ok and item.outcome_accept for item in batch.s7_comparisons))
    batch.final_accept = bool(batch.trent_accept and batch.bob_accept)
    return batch.final_accept


SCENARIOS = (
    "honest",
    "alice_scenario_1",
    "tamper_p2",
    "tamper_p3",
    "impersonation_attempt",
    "bob_tampering_s6_record",
    "replay_record_mismatch",
)


def protocol_analytical_probability(scenario: str, noise: float, lambda_parameter: int) -> tuple[float, float, float]:
    lam = int(lambda_parameter)
    if lam != lambda_parameter or lam < 1:
        raise ValueError("lambda_parameter must be a positive integer")
    if scenario == "replay_record_mismatch":
        return 0.0, 0.0, 0.0
    if scenario == "bob_tampering_s6_record":
        s5 = swap_accept_single(effective_fidelity(1.0, noise)) ** lam
        return 0.0, s5, 0.0

    p_same = swap_accept_single(effective_fidelity(1.0, noise))
    s5_single = s7_single = p_same
    if scenario == "tamper_p2":
        s7_single = swap_accept_single(effective_fidelity(0.0, noise))
    elif scenario in {"tamper_p3", "impersonation_attempt"}:
        probabilities = []
        for key_value in range(16):
            unitary = encryption_unitary(f"{key_value:04b}")
            if scenario == "tamper_p3":
                compared = unitary.conj().T @ X @ unitary @ PLUS
            else:
                compared = unitary.conj().T @ np.array([1, 0], dtype=complex)
            probabilities.append(swap_accept_single(effective_fidelity(fidelity(PLUS, compared), noise)))
        s5_single = float(np.mean(probabilities))
    s5 = s5_single**lam
    s7 = s7_single**lam
    return s5 * s7, s5, s7


def generate_protocol_data(path: Path) -> None:
    rows = []
    for scenario_index, scenario in enumerate(SCENARIOS):
        for noise in NOISE_VALUES:
            for lambda_parameter in PROTOCOL_LAMBDA_VALUES:
                final_count = s5_all_count = s7_all_count = 0
                all_s5_fidelities = []
                all_s7_fidelities = []
                all_s5_effective_fidelities = []
                all_s7_effective_fidelities = []
                s7_reached_count = 0
```

```
            for trial in range(PROTOCOL_TRIALS):
                rng = np.random.default_rng(
                    SEED + scenario_index * 10_000_000 + int(noise * 1000) * 100_000 + lambda_parameter * 10_000 +
trial
                )
                batch = prepare_protocol_batch(lambda_parameter, rng)
                final = execute_protocol_batch(batch, scenario, noise, rng)
                s5_all = batch.trent_accept
                s7_all = batch.bob_accept
                all_s5_fidelities.extend(item.fidelity for item in batch.s5_comparisons)
                all_s5_effective_fidelities.extend(item.effective_fidelity for item in batch.s5_comparisons)
                if batch.s7_comparisons:
                    s7_reached_count += 1
                    all_s7_fidelities.extend(item.fidelity for item in batch.s7_comparisons)
                    all_s7_effective_fidelities.extend(item.effective_fidelity for item in batch.s7_comparisons)
                s5_all_count += int(s5_all)
                s7_all_count += int(s7_all)
                final_count += int(final)
            final_rate = final_count / PROTOCOL_TRIALS
            analytical, analytical_s5, analytical_s7 = protocol_analytical_probability(scenario, noise,
lambda_parameter)
            rows.append(
                {
                    "scenario": scenario,
                    "lambda_parameter": lambda_parameter,
                    "p1_physical_copies": 2 * lambda_parameter + 1,
                    "p2_physical_copies": lambda_parameter,
                    "p3_physical_copies": lambda_parameter,
                    "total_message_copies": 4 * lambda_parameter + 1,
                    "noise_parameter": noise,
                    "noise_model": "F_eff=(1-noise)*F+noise/2 (phenomenological state-level replacement)",
                    "trials": PROTOCOL_TRIALS,
                    "seed_base": SEED,
                    "execution_model": "one physical-copy batch with random S5/S7 sampling without replacement",
                    "physical_comparisons_per_reached_stage": lambda_parameter,
                    "s5_sample_without_replacement": True,
                    "s7_sample_without_replacement": True,
                    "analytical_final_acceptance": analytical,
                    "monte_carlo_final_acceptance": final_rate,
                    "monte_carlo_s5_batch_acceptance": s5_all_count / PROTOCOL_TRIALS,
                    "monte_carlo_s7_batch_acceptance": s7_all_count / PROTOCOL_TRIALS,
                    "s7_reached_rate": s7_reached_count / PROTOCOL_TRIALS,
                    "analytical_s5_batch_acceptance": analytical_s5,
                    "analytical_s7_batch_acceptance": analytical_s7,
                    "false_reject_rate": 1.0 - final_rate if scenario == "honest" else math.nan,
                    "undetected_attack_rate": final_rate if scenario != "honest" else math.nan,
                    "mean_s5_fidelity": float(np.mean(all_s5_fidelities)),
                    "mean_s5_effective_fidelity": float(np.mean(all_s5_effective_fidelities)),
                    "mean_s7_fidelity": float(np.mean(all_s7_fidelities)) if all_s7_fidelities else math.nan,
                    "mean_s7_effective_fidelity": float(np.mean(all_s7_effective_fidelities)) if
all_s7_effective_fidelities else math.nan,
                }
            )
    write_rows(path, rows)


def write_random_sampling_trace(path: Path, lambda_parameter: int = 4) -> None:
    def sample(seed: int) -> ProtocolBatchState:
        rng = np.random.default_rng(seed)
        batch = prepare_protocol_batch(lambda_parameter, rng)
        execute_protocol_batch(batch, "honest", 0.0, rng)
        return batch

    seed = SEED + 4242
    first = sample(seed)
    repeated = sample(seed)
    all_indices = list(range(2 * lambda_parameter + 1))

    def comparison_payload(item: StageComparison) -> dict:
        return {
            "stage": item.stage,
            "p1_index": item.p1_index,
            "reference_index": item.reference_index,
            "ideal_fidelity": item.fidelity,
            "effective_fidelity": item.effective_fidelity,
```

```
            "accept_probability": item.accept_probability,
            "sampled_outcome_accept": item.outcome_accept,
            "records_ok": item.records_ok,
        }

    payload = {
        "seed": seed,
        "lambda_parameter": lambda_parameter,
        "all_p1_indices": all_indices,
        "s5_test_indices": first.s5_test_indices,
        "s5_remaining_indices": first.s5_remaining_indices,
        "s7_test_indices": first.s7_test_indices,
        "retained_p1_index": first.retained_p1_index,
        "s5_comparisons": [comparison_payload(item) for item in first.s5_comparisons],
        "s7_comparisons": [comparison_payload(item) for item in first.s7_comparisons],
        "checks": {
            "s5_no_replacement": len(first.s5_test_indices) == len(set(first.s5_test_indices)),
            "s5_no_overlap": set(first.s5_test_indices).isdisjoint(first.s5_remaining_indices),
            "s5_correct_cardinalities": len(first.s5_test_indices) == lambda_parameter and len(first.s5_remaining_indices) 
== lambda_parameter + 1,
            "s5_partition_complete": sorted(first.s5_test_indices + first.s5_remaining_indices) == all_indices,
            "s7_no_replacement": len(first.s7_test_indices) == len(set(first.s7_test_indices)),
            "s7_correct_cardinality": len(first.s7_test_indices) == lambda_parameter,
            "one_retained_copy": first.retained_p1_index is not None and first.retained_p1_index not in 
first.s7_test_indices,
            "same_seed_deterministic": (first.s5_test_indices, first.s7_test_indices, first.retained_p1_index) == 
(repeated.s5_test_indices, repeated.s7_test_indices, repeated.retained_p1_index),
            "inputs_committed_before_sampling": first.inputs_committed_before_sampling,
        },
    }
    path.write_text(json.dumps(payload, indent=2), encoding="utf-8")


def write_fresh_key_allocation_trace(path: Path, lambda_parameter: int = 4) -> None:
    rng = np.random.default_rng(SEED + 5252)
    batch = prepare_protocol_batch(lambda_parameter, rng)
    blocks = [
        {
            "block_id": block.block_id,
            "purpose": block.purpose,
            "copy_id": block.copy_id,
            "bit_length": len(block.bits),
            "consumption_count": block.consumption_count,
        }
        for block in batch.key_pool.blocks.values()
    ]
    payload = {
        "seed": SEED + 5252,
        "lambda_parameter": lambda_parameter,
        "raw_key_bits_included": False,
        "blocks": blocks,
        "checks": {
            "each_encrypted_object_has_one_block_id": len(blocks) == len(batch.encrypted_object_key_blocks),
            "block_ids_unique": len({row["block_id"] for row in blocks}) == len(blocks),
            "each_block_consumed_once": all(row["consumption_count"] == 1 for row in blocks),
            "raw_bitstrings_required_unique": False,
        },
    }
    path.write_text(json.dumps(payload, indent=2), encoding="utf-8")


def exact_binomial(n: int, alpha: float, beta: float) -> float:
    p = 0.25 + alpha
    return float(sum(math.comb(n, k) * p**k * (1 - p) ** (n - k) for k in range(math.floor(n * beta) + 1)))


def chernoff(n: int, alpha: float, beta: float) -> float:
    p = 0.25 + alpha
    q = beta
    divergence = q * math.log(q / p) + (1 - q) * math.log((1 - q) / (1 - p))
    return math.exp(-n * divergence)


def generate_alice_data(path: Path) -> None:
    rng = np.random.default_rng(SEED)
```

```
    rows = []
    for n in range(10, 201, 10):
        samples = rng.binomial(n, 0.30, size=50_000)
        success = int(np.count_nonzero(samples <= math.floor(n * 0.06)))
        rows.append(
            {
                "n": n,
                "alpha": 0.05,
                "beta": 0.06,
                "p": 0.30,
                "trials": 50_000,
                "success_count": success,
                "monte_carlo_probability": success / 50_000,
                "exact_binomial_probability": exact_binomial(n, 0.05, 0.06),
                "chernoff_upper_bound": chernoff(n, 0.05, 0.06),
                "depends_on_m": False,
            }
        )
    write_rows(path, rows)


def rotation(pauli: np.ndarray, theta: float) -> np.ndarray:
    return math.cos(theta / 2) * I2 - 1j * math.sin(theta / 2) * pauli


def generate_bob_data(path: Path) -> None:
    attacks: list[tuple[str, str, float | None, np.ndarray]] = [
        ("I", "identity", None, I2),
        ("X", "Pauli", None, X),
        ("Y", "Pauli", None, Y),
        ("Z", "Pauli", None, Z),
    ]
    for axis, pauli in (("Rx", X), ("Rz", Z)):
        for theta in (0.01, 0.05, 0.1, 0.2, math.pi / 4, math.pi / 2, math.pi):
            attacks.append((f"{axis}({theta:.12g})", "near-identity rotation" if theta < 0.21 else "rotation", theta,
rotation(pauli, theta)))
    rows = []
    x1_operator = X
    for attack_name, attack_type, theta, G in attacks:
        transformed = x1_operator @ G @ x1_operator @ PLUS
        F_G = fidelity(PLUS, transformed)
        for m in M_VALUES:
            accept = swap_accept_all([F_G] * m)
            rows.append(
                {
                    "attack": attack_name,
                    "attack_type": attack_type,
                    "theta_radians": theta if theta is not None else math.nan,
                    "message_state": "+",
                    "x1_operator": "X",
                    "fidelity_F_G": F_G,
                    "m": m,
                    "acceptance_probability": accept,
                    "detection_probability": 1.0 - accept,
                    "uses_orthogonal_2_to_minus_m_special_case": bool(abs(F_G) < 1e-14),
                }
            )
    write_rows(path, rows)


def generate_resource_data(path: Path) -> None:
    from resource_operation_ledger import resource_scaling_rows

    rows = resource_scaling_rows(range(1, 21), PROTOCOL_LAMBDA_VALUES)
    write_rows(path, rows)


def prepare_fidelity_state(qc: QuantumCircuit, qubit: int, F: float) -> None:
    F = float(F)
    if not np.isfinite(F) or not -1e-12 <= F <= 1.0 + 1e-12:
        raise ValueError("fidelity must be in [0, 1]")
    F = float(np.clip(F, 0.0, 1.0))
    if F < 1.0:
        qc.ry(2.0 * math.acos(math.sqrt(F)), qubit)
```

```python
def build_explicit_independent_swap_circuit(F: float, m: int) -> QuantumCircuit:
    if isinstance(m, bool) or not isinstance(m, (int, np.integer)) or int(m) < 1:
        raise ValueError("m must be a positive integer")
    m = int(m)
    validated_F = float(F)
    if not np.isfinite(validated_F) or not -1e-12 <= validated_F <= 1.0 + 1e-12:
        raise ValueError("fidelity must be in [0, 1]")
    validated_F = float(np.clip(validated_F, 0.0, 1.0))
    q = QuantumRegister(3 * m, "q")
    c = ClassicalRegister(m, "accept")
    qc = QuantumCircuit(q, c, name=f"independent_swap_F_{F:g}_m_{m}")
    for j in range(m):
        ancilla, left, right = 3 * j, 3 * j + 1, 3 * j + 2
        prepare_fidelity_state(qc, right, validated_F)
        qc.h(ancilla)
        qc.cswap(ancilla, left, right)
        qc.h(ancilla)
        qc.measure(ancilla, j)
    qc.metadata = {
        "verification_instances": m,
        "independent_state_preparations": m,
        "fidelity_each_instance": validated_F,
        "direct_hardware": False,
    }
    return qc


def import_composed_candidate(repo_root: Path | None = None, input_state: str = "0"):
    """Build the candidate from the organized local reference module only."""
    del repo_root  # Retained for compatibility with the former call signature.
    from qiskit_protocol_reference import build_composed_staged_emulation_candidate

    return build_composed_staged_emulation_candidate(input_state=input_state)


def qiskit_aer_local_reference(repo_root: Path, output_dir: Path) -> None:
    rows = []
    aer = AerSimulator()
    for F in FIDELITIES:
        for m in PROTOCOL_LAMBDA_VALUES:
            qc = build_explicit_independent_swap_circuit(F, m)
            executable = transpile(qc, aer, optimization_level=1, seed_transpiler=SEED)
            counts = aer.run(executable, shots=AER_SHOTS, seed_simulator=SEED + m).result().get_counts()
            accept_count = int(counts.get("0" * m, 0))
            rows.append(
                {
                    "engine": "qiskit-aer",
                    "experiment": "explicit_independent_swap_test",
                    "fidelity": F,
                    "m": m,
                    "shots": AER_SHOTS,
                    "seed": SEED + m,
                    "logical_qubits": qc.num_qubits,
                    "instance_circuit_count": m,
                    "transpiled_depth": executable.depth(),
                    "accept_count": accept_count,
                    "acceptance_probability": accept_count / AER_SHOTS,
                    "analytical_acceptance_probability": swap_accept_all([F] * m),
                    "counts_json": json.dumps(counts, sort_keys=True),
                }
            )

    composed = import_composed_candidate(repo_root, input_state="0")
    from qiskit_protocol_reference import composed_candidate_expected_outputs, success_probability_from_counts
    valid_outputs = composed_candidate_expected_outputs("0")
    for engine_name, backend in (("qiskit-basic", BasicSimulator()), ("qiskit-aer", aer)):
        executable = transpile(composed, backend, optimization_level=1, seed_transpiler=SEED)
        result = backend.run(executable, shots=4096, seed_simulator=SEED).result()
        counts = result.get_counts()
        acceptance_probability = success_probability_from_counts(counts, valid_outputs)
        success = int(round(acceptance_probability * 4096))
        rows.append(
            {
                "engine": engine_name,
```

```
                "experiment": "composed_candidate_m1_reference",
                "fidelity": math.nan,
                "m": 1,
                "shots": 4096,
                "seed": SEED,
                "logical_qubits": composed.num_qubits,
                "instance_circuit_count": 1,
                "transpiled_depth": executable.depth(),
                "accept_count": success,
                "acceptance_probability": acceptance_probability,
                "analytical_acceptance_probability": 1.0,
                "counts_json": json.dumps(counts, sort_keys=True),
            }
        )

    # Execute fresh copies of the complete composed candidate serially. Each j has
    # its own circuit object and simulator seed; aligned shot records are combined
    # only after every instance has run, so this is not a power of one estimate.
    for m in PROTOCOL_LAMBDA_VALUES:
        per_instance_memories = []
        depths = []
        for j in range(m):
            fresh_candidate = import_composed_candidate(repo_root, input_state="0")
            executable = transpile(fresh_candidate, aer, optimization_level=1, seed_transpiler=SEED + j)
            result = aer.run(executable, shots=4096, seed_simulator=SEED + 1000 + j, memory=True).result()
            valid = composed_candidate_expected_outputs("0")
            per_instance_memories.append(
                np.asarray(["".join(value.split()) in valid for value in result.get_memory()], dtype=bool)
            )
            depths.append(executable.depth())
        all_accept = np.all(np.vstack(per_instance_memories), axis=0)
        accept_count = int(np.count_nonzero(all_accept))
        rows.append(
            {
                "engine": "qiskit-aer",
                "experiment": "composed_candidate_serial_reference",
                "fidelity": math.nan,
                "m": m,
                "shots": 4096,
                "seed": SEED,
                "logical_qubits": composed.num_qubits,
                "instance_circuit_count": m,
                "transpiled_depth": max(depths),
                "accept_count": accept_count,
                "acceptance_probability": accept_count / 4096,
                "analytical_acceptance_probability": 1.0,
                "counts_json": json.dumps({"all_instances_accept": accept_count, "one_or_more_reject": 4096 -
accept_count}),
            }
        )
    write_rows(output_dir / "qiskit_aer_local_reference.csv", rows)


def plot_swap(data_dir: Path, figure_dir: Path) -> None:
    data = pd.read_csv(data_dir / "swap_test_detection.csv")
    colors = plt.cm.viridis(np.linspace(0.08, 0.92, len(FIDELITIES)))
    fig, ax = plt.subplots(figsize=(9.2, 5.8))
    for F, color in zip(FIDELITIES, colors):
        part = data[data.fidelity == F]
        ax.plot(part.m, part.analytical_detection_probability, color=color, lw=1.8, label=f"F={F:g} analytical")
        ax.scatter(part.m, part.monte_carlo_detection_probability, facecolors="none", edgecolors=color, s=34,
label=f"F={F:g} Monte Carlo")
    ax.set(xlabel="Independent verification instances m", ylabel="Detection probability", ylim=(-0.02, 1.02))
    ax.set_xticks(M_VALUES)
    ax.grid(alpha=0.25)
    ax.legend(ncol=2, fontsize=8)
    fig.tight_layout()
    for ext in ("pdf", "png"):
        fig.savefig(figure_dir / f"swap_test_detection.{ext}", dpi=300, bbox_inches="tight")
    plt.close(fig)

    fig, ax = plt.subplots(figsize=(8.4, 5.2))
    for F, color, marker in ((0.9, "#2a6fbb", "o"), (0.99, "#c25b24", "s")):
        part = data[data.fidelity == F]
        ax.plot(part.m, part.analytical_detection_probability, color=color, lw=2, label=f"F={F:g} analytical")
```

```
        ax.scatter(part.m, part.monte_carlo_detection_probability, facecolors="none", edgecolors=color, marker=marker,
s=48, label=f"F={F:g} Monte Carlo")
    ax.set(xlabel="Independent verification instances m", ylabel="Detection probability", ylim=(-0.02, 1.02))
    ax.set_xticks(M_VALUES)
    ax.grid(alpha=0.25)
    ax.legend(fontsize=9)
    fig.tight_layout()
    for ext in ("pdf", "png"):
        fig.savefig(figure_dir / f"high_fidelity_swap_test_detection.{ext}", dpi=300, bbox_inches="tight")
    plt.close(fig)


def plot_bob(data_dir: Path, figure_dir: Path) -> None:
    data = pd.read_csv(data_dir / "bob_attack_fidelity_detection.csv")
    fig, ax = plt.subplots(figsize=(8.6, 5.4))
    for m, color in zip(PROTOCOL_LAMBDA_VALUES, ("#284b63", "#3c6e71", "#d9a441", "#c65d3b")):
        part = data[data.m == m].sort_values("fidelity_F_G")
        ax.plot(part.fidelity_F_G, part.detection_probability, marker="o", ms=3, color=color, label=f"m={m}")
    ax.set(xlabel=r"Attack-state fidelity $F_G$", ylabel="Detection probability", xlim=(-0.02, 1.02), ylim=(-0.02, 1.02))
    ax.grid(alpha=0.25)
    ax.legend()
    fig.tight_layout()
    for ext in ("pdf", "png"):
        fig.savefig(figure_dir / f"bob_attack_detection_vs_fidelity.{ext}", dpi=300, bbox_inches="tight")
    plt.close(fig)


def plot_figure6(data_dir: Path, figure_dir: Path) -> None:
    swap = pd.read_csv(data_dir / "swap_test_detection.csv")
    protocol = pd.read_csv(data_dir / "protocol_acceptance.csv")
    alice = pd.read_csv(data_dir / "alice_repudiation_probability.csv")
    resources = pd.read_csv(data_dir / "resource_scaling.csv")
    fig, axes = plt.subplots(2, 2, figsize=(13.0, 9.4))
    colors = plt.cm.viridis(np.linspace(0.08, 0.92, len(FIDELITIES)))
    for F, color in zip(FIDELITIES, colors):
        part = swap[swap.fidelity == F]
        axes[0, 0].plot(part.m, part.analytical_detection_probability, color=color, label=f"F={F:g}")
        axes[0, 0].scatter(part.m, part.monte_carlo_detection_probability, facecolors="none", edgecolors=color, s=22)
    axes[0, 0].set(title="(a) Swap-test detection", xlabel="Independent verification instances m", ylabel="Detection
probability", ylim=(-0.02, 1.02))
    axes[0, 0].set_xticks(M_VALUES)
    axes[0, 0].legend(ncol=2, fontsize=8, title="Lines: analytical; circles: Monte Carlo")

    selected = protocol[protocol.noise_parameter == 0.02]
    scenario_labels = {
        "honest": "Honest",
        "alice_scenario_1": "Alice scenario 1",
        "tamper_p2": "Tamper p2",
        "tamper_p3": "Tamper p3",
        "impersonation_attempt": "Impersonation",
        "bob_tampering_s6_record": "Bob record tamper",
        "replay_record_mismatch": "Replay mismatch",
    }
    overlap_styles = {
        "bob_tampering_s6_record": {
            "color": "#8c564b",
            "linestyle": "--",
            "marker": "x",
            "markersize": 7,
            "markeredgewidth": 1.6,
            "zorder": 6,
        },
        "replay_record_mismatch": {
            "color": "#4d4d4d",
            "linestyle": ":",
            "marker": "s",
            "markersize": 8,
            "markerfacecolor": "none",
            "markeredgewidth": 1.4,
            "zorder": 5,
        },
    }
    for scenario, label in scenario_labels.items():
        part = selected[selected.scenario == scenario]
        style = overlap_styles.get(scenario, {"marker": "o"})
```

```
        axes[0, 1].plot(
            part.lambda_parameter,
            part.monte_carlo_final_acceptance,
            label=label,
            **style,
        )
    axes[0, 1].set(title="(b) Protocol-batch acceptance (noise=0.02)", xlabel=r"Physical-copy batch parameter $\lambda$",
ylabel="Final acceptance", ylim=(-0.02, 1.02))
    axes[0, 1].set_xticks(PROTOCOL_LAMBDA_VALUES)
    axes[0, 1].legend(fontsize=7)

    axes[1, 0].semilogy(alice.n, alice.exact_binomial_probability, color="black", label="Exact binomial")
    axes[1, 0].scatter(alice.n, alice.monte_carlo_probability.clip(lower=1 / alice.trials), facecolors="none",
edgecolors="#356a9a", label="Monte Carlo")
    axes[1, 0].semilogy(alice.n, alice.chernoff_upper_bound, "--", color="#b45f3c", label="Chernoff bound")
    axes[1, 0].set(title=r"(c) Alice repudiation model (independent of $\lambda$)", xlabel="Transmission sample size n",
ylabel="Probability")
    axes[1, 0].legend(fontsize=8)

    part = resources[resources.lambda_parameter == 8]
    for column, label in (
        ("message_physical_qubit_instances", "Physical message-qubit instances"),
        ("total_bell_pairs", "Bell pairs"),
        ("total_controlled_swap_gates", "Controlled-SWAP gates"),
        ("fresh_qotp_key_bits", "Fresh QOTP key bits"),
    ):
        axes[1, 1].plot(part.n, part[column], label=label)
    axes[1, 1].set(title=r"(d) Physical-copy resource accounting ($\lambda=8$)", xlabel="Message qubits n",
ylabel="Count")
    axes[1, 1].yaxis.set_major_formatter(StrMethodFormatter("{x:,.0f}"))
    axes[1, 1].legend(fontsize=8)
    for ax in axes.ravel():
        ax.grid(alpha=0.22)
    fig.tight_layout()
    for ext in ("pdf", "png"):
        fig.savefig(figure_dir / f"Figure6_protocol_batch.{ext}", dpi=300, bbox_inches="tight")
    plt.close(fig)


def wilson(successes: int, shots: int, z: float = 1.959963984540054) -> tuple[float, float]:
    p = successes / shots
    denominator = 1 + z * z / shots
    center = (p + z * z / (2 * shots)) / denominator
    half = z * math.sqrt(p * (1 - p) / shots + z * z / (4 * shots * shots)) / denominator
    return center - half, center + half


def plot_figure7(repo_root: Path, data_dir: Path, figure_dir: Path) -> None:
    bundled_ibm_dir = repo_root / "data/ibm_hardware_existing"
    ibm_dir = bundled_ibm_dir if bundled_ibm_dir.exists() else repo_root / "paper/data/ibm"
    tele = pd.read_csv(ibm_dir / "teleportation_hardware.csv")
    qotp = pd.read_csv(ibm_dir / "qotp_hardware.csv")
    swap = pd.read_csv(ibm_dir / "swap_test_hardware.csv")
    aer = pd.read_csv(ibm_dir / "composed_candidate_aer.csv").iloc[0]
    pilot = pd.read_csv(ibm_dir / "composed_candidate_pilot.csv").iloc[0]
    final = pd.read_csv(ibm_dir / "composed_candidate_final.csv").iloc[0]

    classifications = []
    for _, row in swap.iterrows():
        direct = int(row["lambda"]) == 1
        classifications.append(
            {
                "source_file": str(ibm_dir / "swap_test_hardware.csv"),
                "execution": row["execution"],
                "case": row["case"],
                "reported_repetition": int(row["lambda"]),
                "DIRECT_HARDWARE": "YES" if direct else "NO",
                "DERIVED_FROM_SINGLE_TEST": "NO" if direct else "YES",
                "job_id": row["job_id"],
                "shots": int(row["shots"]),
                "classification_reason": "raw single-test estimate" if direct else "analytical composition from the same
m=1 hardware estimate",
            }
        )
    write_rows(data_dir / "ibm_hardware_data_classification.csv", classifications)
```

```
    fig, axes = plt.subplots(2, 2, figsize=(11.4, 8.6))
    labels = ["0", "1", "+", "-"]
    t = tele.set_index("input_state").loc[labels]
    axes[0, 0].bar(labels, t.correct_output_probability, color="#355d7a")
    axes[0, 0].set(title="(a) Quantum teleportation", ylabel="Correct-output probability", ylim=(0, 1.05))

    keys = sorted(qotp.key.unique())[:4]
    positions = np.arange(4)
    for index, key in enumerate(keys):
        part = qotp[qotp.key == key].set_index("input_state").reindex(labels)
        axes[0, 1].bar(positions + (index - 1.5) * 0.18, part.correct_output_probability, 0.18, label=f"K={int(key):04d}")
    axes[0, 1].set_xticks(positions, labels)
    axes[0, 1].set(title="(b) Strengthened-QOTP roundtrip", ylabel="Correct-output probability", ylim=(0.94, 1.005))
    axes[0, 1].legend(
        fontsize=8,
        loc="upper left",
        bbox_to_anchor=(1.02, 1.0),
        borderaxespad=0,
    )

    cases = ["identical_0_0", "identical_plus_plus", "orthogonal_0_1", "orthogonal_plus_minus", "fixed_overlap_F_0.25"]
    selected = swap[(swap.execution == "Final") & (swap["lambda"] == 1)].set_index("case").loc[cases]
    ideal_detection = (1 - selected.fidelity.to_numpy()) / 2
    axes[1, 0].bar(np.arange(5) - 0.18, ideal_detection, 0.36, label="Ideal", color="#c9ced3", edgecolor="black")
    axes[1, 0].bar(np.arange(5) + 0.18, selected.single_test_detection_probability, 0.36, label="IBM hardware (direct
m=1)", color="#34495e")
    axes[1, 0].set_xticks(range(5), ["0/0", "+/+", "0/1", "+/-", "F=0.25"])
    axes[1, 0].set(title="(c) Direct single Swap-Test circuits", ylabel="Detection probability", ylim=(0, 0.65))
    axes[1, 0].legend(fontsize=8)

    values = [float(aer.success_probability), float(pilot.success_probability), float(final.success_probability)]
    pilot_interval = wilson(int(pilot.success_count), int(pilot.shots))
    final_interval = wilson(int(final.success_count), int(final.shots))
    low = [0, values[1] - pilot_interval[0], values[2] - final_interval[0]]
    high = [0, pilot_interval[1] - values[1], final_interval[1] - values[2]]
    axes[1, 1].bar(["Aer ideal", "Pilot", "Final"], values, color=["#b8c4cc", "#708090", "#2f4554"])
    axes[1, 1].errorbar(range(3), values, yerr=[low, high], fmt="none", color="black", capsize=4)
    axes[1, 1].set(title="(d) Composed circuit candidate (m=1)", ylabel="Correct-output probability", ylim=(0, 1.05))
    for ax in axes.ravel():
        ax.grid(axis="y", alpha=0.2)
    fig.suptitle("IBM Quantum hardware results and local Aer reference", fontsize=15)
    fig.tight_layout(rect=(0, 0, 0.9, 0.96))
    for ext in ("pdf", "png"):
        fig.savefig(figure_dir / f"Figure7_hardware_scope.{ext}", dpi=300, bbox_inches="tight")
    plt.close(fig)

    write_rows(
        data_dir / "figure7_confidence_intervals.csv",
        [
            {"execution": "Pilot", "success_count": int(pilot.success_count), "shots": int(pilot.shots), "probability":
values[1], "wilson_95_low_recomputed": pilot_interval[0], "wilson_95_high_recomputed": pilot_interval[1]},
            {"execution": "Final", "success_count": int(final.success_count), "shots": int(final.shots), "probability":
values[2], "wilson_95_low_recomputed": final_interval[0], "wilson_95_high_recomputed": final_interval[1]},
        ],
    )


def copy_figure6_source_data(data_dir: Path) -> None:
    source = data_dir / "figure6_source_data"
    source.mkdir(parents=True, exist_ok=True)
    mapping = {
        "panel_a_swap_test.csv": "swap_test_detection.csv",
        "panel_b_protocol_acceptance.csv": "protocol_acceptance.csv",
        "panel_c_alice_repudiation.csv": "alice_repudiation_probability.csv",
        "panel_d_resource_scaling.csv": "resource_scaling.csv",
    }
    for target_name, source_name in mapping.items():
        # pandas round-trip creates standalone panel CSVs from the canonical data.
        pd.read_csv(data_dir / source_name).to_csv(source / target_name, index=False)


def write_run_metadata(output_dir: Path, maximum_swap_error: float) -> None:
    import qiskit
    import qiskit_aer
```

```python
    metadata = {
        "execution_timestamp_utc": datetime.now(timezone.utc).isoformat(),
        "python_version": sys.version,
        "qiskit_version": qiskit.__version__,
        "qiskit_aer_version": qiskit_aer.__version__,
        "numpy_version": np.__version__,
        "pandas_version": pd.__version__,
        "matplotlib_version": matplotlib.__version__,
        "seed": SEED,
        "swap_trials_per_point": SWAP_TRIALS,
        "protocol_trials_per_point": PROTOCOL_TRIALS,
        "aer_shots_per_point": AER_SHOTS,
        "platform": platform.platform(),
        "machine": platform.machine(),
        "maximum_swap_monte_carlo_absolute_error": maximum_swap_error,
        "ibm_submission_attempted": False,
    }
    (output_dir / "data/run_metadata.json").write_text(json.dumps(metadata, indent=2), encoding="utf-8")


def run(output_dir: Path, repo_root: Path) -> None:
    data_dir = output_dir / "data"
    figure_dir = output_dir / "figures"
    data_dir.mkdir(parents=True, exist_ok=True)
    figure_dir.mkdir(parents=True, exist_ok=True)
    maximum_error = generate_swap_data(data_dir / "swap_test_detection.csv")
    generate_protocol_data(data_dir / "protocol_acceptance.csv")
    write_random_sampling_trace(data_dir / "random_sampling_trace.json")
    write_fresh_key_allocation_trace(data_dir / "fresh_key_allocation_trace.json")
    generate_alice_data(data_dir / "alice_repudiation_probability.csv")
    generate_bob_data(data_dir / "bob_attack_fidelity_detection.csv")
    generate_resource_data(data_dir / "resource_scaling.csv")
    qiskit_aer_local_reference(repo_root, data_dir)
    plot_swap(data_dir, figure_dir)
    plot_bob(data_dir, figure_dir)
    plot_figure6(data_dir, figure_dir)
    plot_figure7(repo_root, data_dir, figure_dir)
    copy_figure6_source_data(data_dir)
    write_run_metadata(output_dir, maximum_error)


if __name__ == "__main__":
    parser = argparse.ArgumentParser()
    parser.add_argument("--output-dir", type=Path, required=True)
    parser.add_argument("--repo-root", type=Path, required=True)
    args = parser.parse_args()
    run(args.output_dir.resolve(), args.repo_root.resolve())
```

verification_experiment_validation.py

```
"""Regression validation for the physical-copy protocol-batch implementation."""

from __future__ import annotations

import sys
from pathlib import Path

import numpy as np
import pandas as pd
import pytest
from qiskit import transpile
from qiskit.providers.basic_provider import BasicSimulator
from qiskit_aer import AerSimulator


QISKIT_DIR = Path(__file__).resolve().parent
HARDWARE_DIR = QISKIT_DIR.parent / "ibm_hardware"
for directory in (QISKIT_DIR, HARDWARE_DIR):
    if str(directory) not in sys.path:
        sys.path.insert(0, str(directory))

import ibm_hardware_circuit_generation as HARDWARE_CIRCUITS
import ibm_offline_analysis as IBM_OFFLINE
import local_reference_evaluation as LOCAL
import qiskit_protocol_reference as REFERENCE
import resource_operation_ledger as LEDGER
import verification_experiment_suite as MODEL


def execute_honest_batch(lambda_parameter: int, seed: int = MODEL.SEED):
    rng = np.random.default_rng(seed)
    batch = MODEL.prepare_protocol_batch(lambda_parameter, rng)
    MODEL.execute_protocol_batch(batch, "honest", 0.0, rng)
    return batch


def test_general_fidelity_product():
    fidelities = [0.0, 0.25, 0.9]
    expected = np.prod([(1 + value) / 2 for value in fidelities])
    assert np.isclose(MODEL.swap_accept_all(fidelities), expected)


def test_orthogonal_special_case_only():
    for m in (1, 2, 4, 8, 16):
        assert np.isclose(MODEL.repeated_swap_accept_probability(0.0, m), 2.0**-m)
        assert not np.isclose(MODEL.repeated_swap_accept_probability(0.25, m), 2.0**-m)


def test_lambda_keyword_is_only_a_generic_swap_compatibility_alias():
    assert MODEL.repeated_swap_accept_probability(0.5, lambda_repetitions=4) ==
MODEL.repeated_swap_accept_probability(0.5, m=4)


@pytest.mark.parametrize("lambda_parameter", (1, 2, 4, 8))
def test_protocol_batch_physical_copy_cardinalities(lambda_parameter):
    batch = MODEL.prepare_protocol_batch(lambda_parameter, np.random.default_rng(MODEL.SEED))
    assert len(batch.p1_copies) == 2 * lambda_parameter + 1
    assert len(batch.p2_copies) == lambda_parameter
    assert len(batch.p3_copies) == lambda_parameter
    assert len(batch.p1_copies) + len(batch.p2_copies) + len(batch.p3_copies) == 4 * lambda_parameter + 1


@pytest.mark.parametrize("lambda_parameter", (1, 2, 4, 8))
def test_protocol_batch_records_match_physical_paths(lambda_parameter):
    batch = MODEL.prepare_protocol_batch(lambda_parameter, np.random.default_rng(MODEL.SEED))
    assert len(batch.x1_records) == 2 * lambda_parameter + 1
    assert len(batch.x2_records) == 2 * lambda_parameter + 1
    assert len(batch.x3_records) == lambda_parameter + 1


def test_all_physical_message_arrays_are_distinct_allocations():
    batch = MODEL.prepare_protocol_batch(8, np.random.default_rng(MODEL.SEED))
    arrays = batch.p1_copies + batch.p2_copies + batch.p3_copies
```

```python
    assert len({id(value) for value in arrays}) == len(arrays)


@pytest.mark.parametrize("lambda_parameter", (1, 2, 4, 8))
def test_s5_sampling_without_replacement(lambda_parameter):
    batch = execute_honest_batch(lambda_parameter)
    all_indices = set(range(2 * lambda_parameter + 1))
    assert len(batch.s5_test_indices) == lambda_parameter
    assert len(batch.s5_test_indices) == len(set(batch.s5_test_indices))
    assert len(batch.s5_remaining_indices) == lambda_parameter + 1
    assert set(batch.s5_test_indices).isdisjoint(batch.s5_remaining_indices)
    assert set(batch.s5_test_indices) | set(batch.s5_remaining_indices) == all_indices
    assert batch.inputs_committed_before_sampling


@pytest.mark.parametrize("lambda_parameter", (1, 2, 4, 8))
def test_s7_sampling_and_unique_retained_copy(lambda_parameter):
    batch = execute_honest_batch(lambda_parameter)
    assert len(batch.s7_test_indices) == lambda_parameter
    assert len(batch.s7_test_indices) == len(set(batch.s7_test_indices))
    assert set(batch.s7_test_indices).issubset(batch.s5_remaining_indices)
    retained = set(batch.s5_remaining_indices) - set(batch.s7_test_indices)
    assert retained == {batch.retained_p1_index}


def test_same_seed_reproduces_sampling():
    first = execute_honest_batch(4, MODEL.SEED + 100)
    second = execute_honest_batch(4, MODEL.SEED + 100)
    assert (first.s5_test_indices, first.s7_test_indices, first.retained_p1_index) == (
        second.s5_test_indices,
        second.s7_test_indices,
        second.retained_p1_index,
    )


def test_different_seeds_can_produce_different_sampling():
    selections = {
        (
            tuple(execute_honest_batch(4, MODEL.SEED + offset).s5_test_indices),
            tuple(execute_honest_batch(4, MODEL.SEED + offset).s7_test_indices),
        )
        for offset in range(8)
    }
    assert len(selections) > 1


@pytest.mark.parametrize("lambda_parameter", (1, 2, 4, 8))
def test_each_stage_executes_lambda_explicit_comparisons(lambda_parameter):
    batch = execute_honest_batch(lambda_parameter)
    assert len(batch.s5_comparisons) == lambda_parameter
    assert len(batch.s7_comparisons) == lambda_parameter
    assert all(item.stage == "S5" for item in batch.s5_comparisons)
    assert all(item.stage == "S7" for item in batch.s7_comparisons)


@pytest.mark.parametrize("lambda_parameter", (1, 2, 4, 8))
def test_honest_ideal_batch_accepts(lambda_parameter):
    batch = execute_honest_batch(lambda_parameter)
    assert batch.trent_accept and batch.bob_accept and batch.final_accept


def test_noise_is_applied_per_physical_comparison():
    rng = np.random.default_rng(MODEL.SEED)
    batch = MODEL.prepare_protocol_batch(4, rng)
    MODEL.execute_protocol_batch(batch, "honest", 0.02, rng)
    for item in batch.s5_comparisons + batch.s7_comparisons:
        assert np.isclose(item.effective_fidelity, 0.99)
        assert np.isclose(item.accept_probability, 0.995)


def test_record_mismatch_scenarios_retain_zero_final_acceptance():
    for scenario in ("bob_tampering_s6_record", "replay_record_mismatch"):
        rng = np.random.default_rng(MODEL.SEED)
        batch = MODEL.prepare_protocol_batch(4, rng)
        assert MODEL.execute_protocol_batch(batch, scenario, 0.0, rng) is False
```

```python
        assert batch.attacked_index == 0


def test_alice_consistency_only_scenario_remains_indistinguishable_in_ideal_model():
    rng = np.random.default_rng(MODEL.SEED)
    batch = MODEL.prepare_protocol_batch(4, rng)
    assert MODEL.execute_protocol_batch(batch, "alice_scenario_1", 0.0, rng) is True


@pytest.mark.parametrize("lambda_parameter", (1, 2, 4, 8))
def test_bell_allocation_by_stage(lambda_parameter):
    row = LEDGER.summarize_resources(1, lambda_parameter)
    assert row["s2_bell_pairs"] == 2 * lambda_parameter + 1
    assert row["s4_bell_pairs"] == 2 * lambda_parameter + 1
    assert row["s6_bell_pairs"] == lambda_parameter + 1
    assert row["total_bell_pairs"] == 5 * lambda_parameter + 3


@pytest.mark.parametrize(("n", "lambda_parameter"), ((1, 1), (1, 8), (7, 4), (20, 8)))
def test_resource_physical_copy_batch_formulas(n, lambda_parameter):
    row = LEDGER.summarize_resources(n, lambda_parameter)
    assert row["message_physical_qubit_instances"] == (4 * lambda_parameter + 1) * n
    assert row["total_bell_pairs"] == (5 * lambda_parameter + 3) * n
    assert row["total_bell_qubits"] == 2 * (5 * lambda_parameter + 3) * n
    assert row["teleportation_operations"] == (5 * lambda_parameter + 3) * n
    assert row["total_controlled_swap_gates"] == 2 * lambda_parameter * n


@pytest.mark.parametrize("lambda_parameter", (1, 2, 4, 8))
def test_fresh_key_total_is_derived_from_encrypted_objects(lambda_parameter):
    objects = LEDGER.encrypted_object_ledger(lambda_parameter)
    derived = sum(item.bit_length for item in objects)
    assert derived == 64 * lambda_parameter + 36
    assert LEDGER.summarize_resources(1, lambda_parameter)["fresh_qotp_key_bits"] == derived


def test_qotp_step_attribution_preserves_total_and_matches_protocol_flow():
    lambda_parameter = 4
    n = 3
    steps = {row.step: row for row in LEDGER.protocol_step_ledger(n, lambda_parameter)}
    objects = LEDGER.encrypted_object_ledger(lambda_parameter)
    assert all(item.encryption_step == "S3" and item.recovery_step == "S5" for item in objects if item.purpose ==
"p3_key")
    assert steps["S1"].fresh_qotp_key_bits == 0
    assert steps["S3"].fresh_qotp_key_bits == n * sum(item.bit_length for item in objects if item.encryption_step == "S3")
    assert steps["S5"].qotp_unitary_applications == n * sum(1 for item in objects if item.recovery_step == "S5")
    assert steps["S7"].qotp_unitary_applications == n * sum(1 for item in objects if item.recovery_step == "S7")
    assert sum(row.qotp_unitary_applications for row in steps.values()) == 2 * n * len(objects)


def test_resource_total_is_sum_of_step_ledger():
    n, lambda_parameter = 5, 4
    steps = LEDGER.protocol_step_ledger(n, lambda_parameter)
    row = LEDGER.summarize_resources(n, lambda_parameter)
    assert row["total_modeled_operations"] == sum(item.modeled_operations for item in steps)
    assert row["total_modeled_operations"] != lambda_parameter * (40 * n + 2)


def test_ambiguous_communication_and_peak_resources_are_not_guessed():
    row = LEDGER.summarize_resources(3, 2)
    assert row["quantum_transmitted_qubits"] == LEDGER.REQUIRES_CONFIRMATION
    assert row["classical_transmitted_bits"] == LEDGER.REQUIRES_CONFIRMATION
    assert row["peak_live_qubits"] == LEDGER.REQUIRES_CONFIRMATION


@pytest.mark.parametrize("lambda_parameter", (1, 2, 4, 8))
def test_fresh_key_allocator_assigns_unique_single_use_block_ids(lambda_parameter):
    batch = MODEL.prepare_protocol_batch(lambda_parameter, np.random.default_rng(MODEL.SEED))
    blocks = list(batch.key_pool.blocks.values())
    assert len(blocks) == len(batch.encrypted_object_key_blocks)
    assert len({block.block_id for block in blocks}) == len(blocks)
    assert all(block.consumption_count == 1 and block.consumed for block in blocks)


def test_fresh_key_block_reuse_raises():
```

```python
    pool = MODEL.FreshKeyPool(np.random.default_rng(MODEL.SEED))
    block = pool.allocate(4, "diagnostic", "copy[0]")
    pool.consume(block.block_id)
    with pytest.raises(RuntimeError, match="reused"):
        pool.consume(block.block_id)


def test_fresh_key_policy_does_not_require_unique_bit_patterns():
    pool = MODEL.FreshKeyPool(np.random.default_rng(MODEL.SEED))
    blocks = [pool.allocate(1, "diagnostic", f"copy[{i}]") for i in range(16)]
    for block in blocks:
        pool.consume(block.block_id)
    assert len({block.block_id for block in blocks}) == len(blocks)
    assert {block.bits for block in blocks}.issubset({"0", "1"})
    assert all(block.consumption_count == 1 for block in blocks)


def test_fidelity_zero_vector_rejected():
    with pytest.raises(ValueError):
        MODEL.fidelity(np.zeros(2), np.array([1, 0]))


@pytest.mark.parametrize(
    ("left", "right", "expected"),
    [
        (np.array([2, 0]), np.array([7, 0]), 1.0),
        (np.array([1, 0]), np.array([0, 1]), 0.0),
        (np.array([1, 1]) * (1 + 1e-14), np.array([1, 1]), 1.0),
        (np.array([1, 1j]), np.exp(0.73j) * np.array([1, 1j]), 1.0),
    ],
)
def test_fidelity_valid_and_global_phase_invariant(left, right, expected):
    value = MODEL.fidelity(left, right)
    assert 0.0 <= value <= 1.0
    assert np.isclose(value, expected)


@pytest.mark.parametrize(("F", "m", "expected"), ((1.0, 8, 0.0), (0.0, 8, 1 - 2**-8)))
def test_swap_test_endpoints(F, m, expected):
    assert np.isclose(MODEL.swap_detection([F] * m), expected)


@pytest.mark.parametrize(("F", "m"), ((-0.1, 1), (1.1, 1), (0.5, 0), (0.5, -1), (0.5, 1.5)))
def test_explicit_swap_test_invalid_parameters(F, m):
    with pytest.raises(ValueError):
        MODEL.build_explicit_independent_swap_circuit(F, m)


def test_explicit_qiskit_swap_circuit_has_m_preparations_and_measurements():
    circuit = MODEL.build_explicit_independent_swap_circuit(0.9, 8)
    assert circuit.metadata["verification_instances"] == 8
    assert circuit.num_qubits == 24
    assert circuit.num_clbits == 8


@pytest.mark.parametrize("state", LOCAL.VALIDATION_STATES)
def test_single_teleportation_six_states(state):
    assert np.isclose(LOCAL.coherent_chain_fidelity(state, 1), 1.0, atol=1e-12)
    assert min(LOCAL.conditioned_outcome_fidelities(state).values()) >= 1 - 1e-12


@pytest.mark.parametrize("state", LOCAL.VALIDATION_STATES)
def test_three_sequential_teleportations_six_states(state):
    for rounds in (1, 2, 3):
        assert np.isclose(LOCAL.coherent_chain_fidelity(state, rounds), 1.0, atol=1e-12)


@pytest.mark.parametrize("state", ("0", "1", "+", "-", "i+"))
def test_composed_candidate_expected_outputs_aer(state):
    backend = AerSimulator()
    circuit = REFERENCE.build_composed_staged_emulation_candidate(state)
    executable = transpile(circuit, backend, optimization_level=1, seed_transpiler=MODEL.SEED)
    counts = backend.run(executable, shots=256, seed_simulator=MODEL.SEED).result().get_counts()
    expected = REFERENCE.composed_candidate_expected_outputs(state)
    assert REFERENCE.success_probability_from_counts(counts, expected) == 1.0
```

```python
@pytest.mark.parametrize("state", ("0", "1", "+", "-"))
def test_composed_candidate_expected_outputs_basic_simulator(state):
    backend = BasicSimulator()
    circuit = REFERENCE.build_composed_staged_emulation_candidate(state)
    executable = transpile(circuit, backend, optimization_level=0, seed_transpiler=MODEL.SEED)
    counts = backend.run(executable, shots=128, seed_simulator=MODEL.SEED).result().get_counts()
    expected = REFERENCE.composed_candidate_expected_outputs(state)
    assert REFERENCE.success_probability_from_counts(counts, expected) == 1.0


def test_composed_candidate_global_phase_equivalence():
    state = LOCAL.named_statevector("i+")
    assert MODEL.fidelity(state, np.exp(1j * np.pi / 3) * state) == 1.0


def test_success_probability_sums_multiple_valid_outputs_and_normalizes_spaces():
    counts = {"0 00": 3, "1 00": 5, "0 01": 2}
    assert REFERENCE.success_probability_from_counts(counts, {"000", "100"}) == 0.8


def test_organized_composed_import_does_not_require_experiments_tree():
    circuit = MODEL.import_composed_candidate(Path("/path/without/experiments"), input_state="1")
    assert circuit.metadata["valid_expected_outputs"] == ["100"]


def test_ibm_offline_m1_and_derived_classification(tmp_path):
    output = tmp_path / "ibm_offline.csv"
    IBM_OFFLINE.analyze_existing_hardware(output)
    data = pd.read_csv(output)
    composed = data[data.record_type == "composed_candidate_final"].iloc[0]
    assert composed.m == 1 and composed.DIRECT_HARDWARE == "YES"
    swap = data[data.record_type == "swap_test"]
    assert set(swap.loc[swap.DIRECT_HARDWARE == "YES", "m"]) == {1}
    assert set(swap.loc[swap.DERIVED_FROM_SINGLE_TEST == "YES", "m"]) == {2, 4, 8}
    assert data.absolute_difference.max() < 1e-12


def test_default_hardware_target_generation_never_initializes_ibm(monkeypatch):
    initialized = False

    class ForbiddenService:
        def __init__(self, *args, **kwargs):
            nonlocal initialized
            initialized = True

    monkeypatch.setattr(HARDWARE_CIRCUITS, "QiskitRuntimeService", ForbiddenService)
    with pytest.raises(RuntimeError, match="offline mode is the default"):
        HARDWARE_CIRCUITS.generate_ibm_target_circuits(connect_ibm=False)
    assert initialized is False
```
