# Supplementary material for: On a Grover-Based Quantum Signature Scheme and a Teleportation-Based Design
Source: Entropy (Basel). 2026 Sep 18;28(9):1030. doi: 10.3390/e28091030 (PMC13605562; doi:10.3390/e28091030)
Supplement: Supplementary file 1 [file entropy-28-01030-s001.zip › Supplementary Material-S3-Figure_7_Full Python Code.pdf]

Figure 7 Full Code

IBM Aer Simulation

aer_circuit_generation.py

```
"""Complete IBM/Qiskit circuit, retrieval, evaluation, and guarded-submission code."""

from __future__ import annotations

import argparse
import csv
import hashlib
import json
from math import sqrt
from pathlib import Path

from qiskit import ClassicalRegister, QuantumCircuit, QuantumRegister, transpile
from qiskit.qasm3 import dumps as qasm3_dumps
from qiskit.transpiler import generate_preset_pass_manager


# 1. Bell resources

def build_bell_pair_circuit(name='bell_pair'):
    q = QuantumRegister(2, 'bell')
    qc = QuantumCircuit(q, name=name)
    qc.h(q[0])
    qc.cx(q[0], q[1])
    return qc

def build_three_bell_pair_circuit():
    q = QuantumRegister(6, 'bell')
    qc = QuantumCircuit(q, name='three_v7_bell_pairs')
    for a, b in ((0, 1), (2, 3), (4, 5)):
        qc.h(q[a])
        qc.cx(q[a], q[b])
    qc.metadata = {'pairs': {'AB_S2': [0, 1], 'BT_S4': [2, 3], 'TB_S6': [4, 5]}}
    return qc

# 2. Teleportation

def coherent_teleportation(qc, message, bell_sender, bell_receiver, label):
    qc.barrier(label=f'{label}_Bell_measurement')
    qc.cx(message, bell_sender)
    qc.h(message)
    qc.cx(bell_sender, bell_receiver)
    qc.cz(message, bell_receiver)

def build_teleportation_circuit(input_state='0'):
    qc = QuantumCircuit(3, 1, name=f'teleport_{input_state}')
    if input_state == '1':
        qc.x(0)
    elif input_state == '+':
        qc.h(0)
    elif input_state == '-':
        qc.x(0)
        qc.h(0)
    qc.h(1)
    qc.cx(1, 2)
    coherent_teleportation(qc, 0, 1, 2, 'S2')
    if input_state in {'+', '-'}:
        qc.h(2)
    qc.measure(2, 0)
    return qc

def build_three_teleportation_rounds(input_state='0'):
    qc = QuantumCircuit(7, 1, name='three_teleportation_rounds')
    if input_state == '1':
        qc.x(0)
    elif input_state == '+':
        qc.h(0)
    elif input_state == '-':
```

```
        qc.x(0)
        qc.h(0)
    for a, b in ((1, 2), (3, 4), (5, 6)):
        qc.h(a)
        qc.cx(a, b)
    coherent_teleportation(qc, 0, 1, 2, 'S2')
    coherent_teleportation(qc, 2, 3, 4, 'S4')
    coherent_teleportation(qc, 4, 5, 6, 'S6')
    if input_state in {'+', '-'}:
        qc.h(6)
    qc.measure(6, 0)
    return qc

# 3. Strengthened-QOTP

def append_qotp(qc, qubit, key4, label='E_K'):
    U = encryption_unitary(key4)
    qc.unitary(U, [qubit], label=label)

def append_qotp_inverse(qc, qubit, key4, label='E_K^-1'):
    U = encryption_unitary(key4)
    qc.unitary(U.conj().T, [qubit], label=label)

def build_qotp_roundtrip_circuit(key4='0101', input_state='0'):
    qc = QuantumCircuit(1, 1, name='strengthened_qotp_roundtrip')
    if input_state == '1':
        qc.x(0)
    elif input_state == '+':
        qc.h(0)
    elif input_state == '-':
        qc.x(0)
        qc.h(0)
    append_qotp(qc, 0, key4)
    append_qotp_inverse(qc, 0, key4)
    if input_state in {'+', '-'}:
        qc.h(0)
    qc.measure(0, 0)
    return qc

# 4. Encrypted Bell records

def append_encrypted_record_roundtrip(qc, carrier, key4, record, label):
    if record[1] == '1':
        qc.x(carrier)
    if record[0] == '1':
        qc.z(carrier)
    append_qotp(qc, carrier, key4, f'{label}_encrypt')
    append_qotp_inverse(qc, carrier, key4, f'{label}_decrypt')
    if record[0] == '1':
        qc.z(carrier)
    if record[1] == '1':
        qc.x(carrier)

def build_encrypted_bell_record_circuit(key4='1010', record='01'):
    qc = QuantumCircuit(2, 2, name='encrypted_bell_record_roundtrip')
    qc.h(0)
    qc.cx(0, 1)
    append_encrypted_record_roundtrip(qc, 1, key4, record, 'E_KU_X')
    qc.cx(0, 1)
    qc.h(0)
    qc.measure([0, 1], [0, 1])
    return qc

# 5. Repeated swap tests

def build_repeated_swap_test_circuits(left='0', right='1', lam=8):
    return [build_swap_test_circuit(left, right, name=f'swap_{index + 1}_of_{lam}') for index in range(lam)]

# 6. Staged components

def build_staged_components():
    return {'S2': build_teleportation_circuit(), 'S3_record': build_encrypted_bell_record_circuit(), 'S5_QOTP':
build_qotp_roundtrip_circuit(), 'S6': build_teleportation_circuit()}

# 7. Composed staged-emulation candidate
```

```
FIXED_KEY = '0101'

def _prepare_message_copies(qc, qubits, input_state):
    for q in qubits:
        if input_state == '1':
            qc.x(q)
        elif input_state == '+':
            qc.h(q)
        elif input_state == '-':
            qc.x(q)
            qc.h(q)

def _swap_test(qc, anc, left, right, label):
    qc.barrier(label=label)
    qc.h(anc)
    qc.cswap(anc, left, right)
    qc.h(anc)

def build_composed_staged_emulation_candidate(input_state='0'):
    q = QuantumRegister(9, 'candidate')
    c = ClassicalRegister(3, 'out')
    qc = QuantumCircuit(q, c, name='composed_staged_emulation_candidate')
    _prepare_message_copies(qc, (0, 1, 2), input_state)
    qc.barrier(label='S1_three_message_copies')
    for a, b, label in ((3, 4, 'AB_S2'), (5, 6, 'BT_S4'), (7, 8, 'TB_S6')):
        qc.h(a)
        qc.cx(a, b)
        qc.barrier(label=f'I2_{label}')
    coherent_teleportation(qc, 0, 3, 4, 'S2')
    append_encrypted_record_roundtrip(qc, 3, FIXED_KEY, '01', 'S3_X1_record')
    coherent_teleportation(qc, 4, 5, 6, 'S4')
    append_encrypted_record_roundtrip(qc, 5, FIXED_KEY, '10', 'S4_X2_record')
    qc.reset(0)
    _swap_test(qc, 0, 6, 2, 'S5_swap_test')
    coherent_teleportation(qc, 6, 7, 8, 'S6')
    append_encrypted_record_roundtrip(qc, 7, FIXED_KEY, '11', 'S6_X3_record')
    qc.reset(4)
    _swap_test(qc, 4, 8, 1, 'S7_swap_test')
    if input_state in {'+', '-'}:
        qc.h(8)
    qc.measure(0, 0)
    qc.measure(4, 1)
    qc.measure(8, 2)
    qc.metadata = {'candidate_type': 'faithful_v7_three_explicit_bell_resources', 'input_state': input_state,
'valid_expected_outputs': ['100'] if input_state in {'1', '-'} else ['000'], 'measurement_order': 'out[2] out[1] out[0]',
'success_definition': 'sum over valid expected outputs', 'paper_resource_qubits': 9, 'swap_ancillas': 'reuse of
consumed/dead measured-stage wires', 'not_implemented': ['QKD', 'authentication', 'network separation', 'abstract
arbitration']}
    return qc

def build_faithful_v7_unitary_reference(input_state='0'):
    qc = QuantumCircuit(11, name='faithful_v7_unitary_reference')
    _prepare_message_copies(qc, (0, 1, 2), input_state)
    for a, b in ((3, 4), (5, 6), (7, 8)):
        qc.h(a)
        qc.cx(a, b)
    coherent_teleportation(qc, 0, 3, 4, 'S2')
    append_encrypted_record_roundtrip(qc, 3, FIXED_KEY, '01', 'S3_X1_record')
    coherent_teleportation(qc, 4, 5, 6, 'S4')
    append_encrypted_record_roundtrip(qc, 5, FIXED_KEY, '10', 'S4_X2_record')
    _swap_test(qc, 9, 6, 2, 'S5_swap_test')
    coherent_teleportation(qc, 6, 7, 8, 'S6')
    append_encrypted_record_roundtrip(qc, 7, FIXED_KEY, '11', 'S6_X3_record')
    _swap_test(qc, 10, 8, 1, 'S7_swap_test')
    if input_state in {'+', '-'}:
        qc.h(8)
    return qc

def build_optimized_staged_candidate(input_state='0'):
    q = QuantumRegister(7, 'opt')
    c = ClassicalRegister(3, 'out')
    qc = QuantumCircuit(q, c, name='optimized_v7_staged_candidate')
    _prepare_message_copies(qc, (0, 1, 2), input_state)
    qc.barrier(label='S1')
```

```
    def leg(label):
        qc.h(3)
        qc.cx(3, 4)
        coherent_teleportation(qc, 0, 3, 4, label)
        qc.swap(0, 4)
        qc.reset(3)
        qc.reset(4)
    leg('S2')
    append_encrypted_record_roundtrip(qc, 6, FIXED_KEY, '01', 'S3_record')
    leg('S4')
    qc.reset(5)
    _swap_test(qc, 5, 0, 2, 'S5_swap_test')
    leg('S6')
    qc.reset(4)
    _swap_test(qc, 4, 0, 1, 'S7_swap_test')
    if input_state in {'+', '-'}:
        qc.h(0)
    qc.measure(5, 0)
    qc.measure(4, 1)
    qc.measure(0, 2)
    qc.metadata = {'candidate_type': 'optimized_staged_reset_reuse', 'input_state': input_state, 'valid_expected_outputs':
['100'] if input_state in {'1', '-'} else ['000'], 'measurement_order': 'out[2] out[1] out[0]', 'success_definition':
'sum over valid expected outputs', 'not_paper_resource_count': True}
    return qc
import csv
import matplotlib.pyplot as plt
from qiskit import qasm3, transpile
from qiskit.qasm3 import dumps as qasm3_dumps

from qiskit_aer import AerSimulator

# Self-contained matrix definitions used by the strengthened-QOTP circuit.
import numpy as np

X_MATRIX = np.array([[0, 1], [1, 0]], dtype=complex)
Y_MATRIX = np.array([[0, -1j], [1j, 0]], dtype=complex)
Z_MATRIX = np.array([[1, 0], [0, -1]], dtype=complex)
T_STRENGTHENED = np.sqrt(1j / 3) * (X_MATRIX - Y_MATRIX + Z_MATRIX)


def encryption_unitary(key4):
    """Return the one-qubit strengthened-QOTP unitary for a four-bit key block."""
    if len(key4) != 4 or set(key4) - {"0", "1"}:
        raise ValueError("key block must contain four binary digits")
    a, b, c, d = map(int, key4)
    return (
        np.linalg.matrix_power(X_MATRIX, a)
        @ np.linalg.matrix_power(Z_MATRIX, b)
        @ T_STRENGTHENED
        @ np.linalg.matrix_power(X_MATRIX, c)
        @ np.linalg.matrix_power(Z_MATRIX, d)
    )


# Circuit collection used for the modular and complete folders.
def build_swap_test_verification_circuit(left="0", right="1"):
    qc = QuantumCircuit(3, 1, name="swap_test_verification")
    if left == "1":
        qc.x(1)
    elif left == "+":
        qc.h(1)
    elif left == "-":
        qc.x(1); qc.h(1)
    if right == "1":
        qc.x(2)
    elif right == "+":
        qc.h(2)
    elif right == "-":
        qc.x(2); qc.h(2)
    qc.h(0); qc.cswap(0, 1, 2); qc.h(0); qc.measure(0, 0)
    return qc


def build_swap_test_circuit(left="0", right="1", name="swap_test"):
```

```python
    """Compatibility wrapper used by the repeated swap-test circuit builder."""
    circuit = build_swap_test_verification_circuit(left, right)
    circuit.name = name
    return circuit


def circuit_collection():
    staged = build_staged_components()
    modular = {
        "Bell_Pair": build_bell_pair_circuit(),
        "Three_Bell_Pairs": build_three_bell_pair_circuit(),
        "Teleportation": build_teleportation_circuit(),
        "Three_Teleportation_Rounds": build_three_teleportation_rounds(),
        "Strengthened_QOTP_Roundtrip": build_qotp_roundtrip_circuit(),
        "Encrypted_Bell_Record": build_encrypted_bell_record_circuit(),
        "Swap_Test_Verification": build_swap_test_verification_circuit(),
        "Stage_S2": staged["S2"],
        "Stage_S3_Record": staged["S3_record"],
        "Stage_S5_QOTP": staged["S5_QOTP"],
        "Stage_S6": staged["S6"],
    }
    complete = {
        "Composed_Full_Candidate": build_composed_staged_emulation_candidate(),
        "Optimized_Staged_Candidate": build_optimized_staged_candidate(),
    }
    return modular, complete


def save_circuit(circuit, base_path):
    base_path.parent.mkdir(parents=True, exist_ok=True)
    (base_path.with_suffix(".qasm")).write_text(qasm3_dumps(circuit), encoding="utf-8")
    (base_path.with_suffix(".txt")).write_text(str(circuit.draw("text", fold=160)), encoding="utf-8")
    figure = circuit.draw("mpl", style="iqp", fold=80, idle_wires=False)
    for extension in ("pdf", "png", "svg"):
        figure.savefig(base_path.with_suffix("." + extension), dpi=300 if extension == "png" else None,
bbox_inches="tight", facecolor="white")
    plt.close(figure)

def generate_aer_circuits():
    root = Path(__file__).resolve().parents[1] / "results"
    backend = AerSimulator()
    modular, complete = circuit_collection()
    rows = []
    for group, circuits in (("Modular_Circuits", modular), ("Complete_Circuits", complete)):
        for name, logical in circuits.items():
            aer_circuit = transpile(logical, backend=backend, optimization_level=1, seed_transpiler=20260803)
            save_circuit(aer_circuit, root / group / name)
            rows.append({"group": group, "circuit": name, "qubits": aer_circuit.num_qubits, "depth": aer_circuit.depth(),
"size": aer_circuit.size()})
    with (root / "Aer_Transpilation_Metrics.csv").open("w", newline="", encoding="utf-8") as handle:
        writer = csv.DictWriter(handle, fieldnames=list(rows[0])); writer.writeheader(); writer.writerows(rows)


if __name__ == "__main__":
    generate_aer_circuits()
```

aer_complete_pipeline.py

```
"""Complete IBM/Qiskit circuit, retrieval, evaluation, and guarded-submission code."""

from __future__ import annotations

import argparse
import csv
import hashlib
import json
from math import sqrt
from pathlib import Path

from qiskit import ClassicalRegister, QuantumCircuit, QuantumRegister, transpile
from qiskit.qasm3 import dumps as qasm3_dumps
from qiskit.transpiler import generate_preset_pass_manager
try:
    from qiskit_ibm_runtime import QiskitRuntimeService, SamplerV2
except ImportError:  # Optional for local Aer execution.
    QiskitRuntimeService = None
    SamplerV2 = None


# 1. Bell resources

def build_bell_pair_circuit(name='bell_pair'):
    q = QuantumRegister(2, 'bell')
    qc = QuantumCircuit(q, name=name)
    qc.h(q[0])
    qc.cx(q[0], q[1])
    return qc

def build_three_bell_pair_circuit():
    q = QuantumRegister(6, 'bell')
    qc = QuantumCircuit(q, name='three_v7_bell_pairs')
    for a, b in ((0, 1), (2, 3), (4, 5)):
        qc.h(q[a])
        qc.cx(q[a], q[b])
    qc.metadata = {'pairs': {'AB_S2': [0, 1], 'BT_S4': [2, 3], 'TB_S6': [4, 5]}}
    return qc

# 2. Teleportation

def coherent_teleportation(qc, message, bell_sender, bell_receiver, label):
    qc.barrier(label=f'{label}_Bell_measurement')
    qc.cx(message, bell_sender)
    qc.h(message)
    qc.cx(bell_sender, bell_receiver)
    qc.cz(message, bell_receiver)

def build_teleportation_circuit(input_state='0'):
    qc = QuantumCircuit(3, 1, name=f'teleport_{input_state}')
    if input_state == '1':
        qc.x(0)
    elif input_state == '+':
        qc.h(0)
    elif input_state == '-':
        qc.x(0)
        qc.h(0)
    qc.h(1)
    qc.cx(1, 2)
    coherent_teleportation(qc, 0, 1, 2, 'S2')
    if input_state in {'+', '-'}:
        qc.h(2)
    qc.measure(2, 0)
    return qc

def build_three_teleportation_rounds(input_state='0'):
    qc = QuantumCircuit(7, 1, name='three_teleportation_rounds')
    if input_state == '1':
        qc.x(0)
    elif input_state == '+':
        qc.h(0)
    elif input_state == '-':
        qc.x(0)
```

```
        qc.h(0)
    for a, b in ((1, 2), (3, 4), (5, 6)):
        qc.h(a)
        qc.cx(a, b)
    coherent_teleportation(qc, 0, 1, 2, 'S2')
    coherent_teleportation(qc, 2, 3, 4, 'S4')
    coherent_teleportation(qc, 4, 5, 6, 'S6')
    if input_state in {'+', '-'}:
        qc.h(6)
    qc.measure(6, 0)
    return qc

# 3. Strengthened-QOTP

def append_qotp(qc, qubit, key4, label='E_K'):
    U = encryption_unitary(key4)
    qc.unitary(U, [qubit], label=label)

def append_qotp_inverse(qc, qubit, key4, label='E_K^-1'):
    U = encryption_unitary(key4)
    qc.unitary(U.conj().T, [qubit], label=label)

def build_qotp_roundtrip_circuit(key4='0101', input_state='0'):
    qc = QuantumCircuit(1, 1, name='strengthened_qotp_roundtrip')
    if input_state == '1':
        qc.x(0)
    elif input_state == '+':
        qc.h(0)
    elif input_state == '-':
        qc.x(0)
        qc.h(0)
    append_qotp(qc, 0, key4)
    append_qotp_inverse(qc, 0, key4)
    if input_state in {'+', '-'}:
        qc.h(0)
    qc.measure(0, 0)
    return qc

# 4. Encrypted Bell records

def append_encrypted_record_roundtrip(qc, carrier, key4, record, label):
    if record[1] == '1':
        qc.x(carrier)
    if record[0] == '1':
        qc.z(carrier)
    append_qotp(qc, carrier, key4, f'{label}_encrypt')
    append_qotp_inverse(qc, carrier, key4, f'{label}_decrypt')
    if record[0] == '1':
        qc.z(carrier)
    if record[1] == '1':
        qc.x(carrier)

def build_encrypted_bell_record_circuit(key4='1010', record='01'):
    qc = QuantumCircuit(2, 2, name='encrypted_bell_record_roundtrip')
    qc.h(0)
    qc.cx(0, 1)
    append_encrypted_record_roundtrip(qc, 1, key4, record, 'E_KU_X')
    qc.cx(0, 1)
    qc.h(0)
    qc.measure([0, 1], [0, 1])
    return qc

# 5. Repeated swap tests

def build_repeated_swap_test_circuits(left='0', right='1', lam=8):
    return [build_swap_test_circuit(left, right, name=f'swap_{index + 1}_of_{lam}') for index in range(lam)]

# 6. Staged components

def build_staged_components():
    return {'S2': build_teleportation_circuit(), 'S3_record': build_encrypted_bell_record_circuit(), 'S5_QOTP':
build_qotp_roundtrip_circuit(), 'S6': build_teleportation_circuit()}

# 7. Composed staged-emulation candidate
```

```
FIXED_KEY = '0101'

def _prepare_message_copies(qc, qubits, input_state):
    for q in qubits:
        if input_state == '1':
            qc.x(q)
        elif input_state == '+':
            qc.h(q)
        elif input_state == '-':
            qc.x(q)
            qc.h(q)

def _swap_test(qc, anc, left, right, label):
    qc.barrier(label=label)
    qc.h(anc)
    qc.cswap(anc, left, right)
    qc.h(anc)

def build_composed_staged_emulation_candidate(input_state='0'):
    q = QuantumRegister(9, 'candidate')
    c = ClassicalRegister(3, 'out')
    qc = QuantumCircuit(q, c, name='composed_staged_emulation_candidate')
    _prepare_message_copies(qc, (0, 1, 2), input_state)
    qc.barrier(label='S1_three_message_copies')
    for a, b, label in ((3, 4, 'AB_S2'), (5, 6, 'BT_S4'), (7, 8, 'TB_S6')):
        qc.h(a)
        qc.cx(a, b)
        qc.barrier(label=f'I2_{label}')
    coherent_teleportation(qc, 0, 3, 4, 'S2')
    append_encrypted_record_roundtrip(qc, 3, FIXED_KEY, '01', 'S3_X1_record')
    coherent_teleportation(qc, 4, 5, 6, 'S4')
    append_encrypted_record_roundtrip(qc, 5, FIXED_KEY, '10', 'S4_X2_record')
    qc.reset(0)
    _swap_test(qc, 0, 6, 2, 'S5_swap_test')
    coherent_teleportation(qc, 6, 7, 8, 'S6')
    append_encrypted_record_roundtrip(qc, 7, FIXED_KEY, '11', 'S6_X3_record')
    qc.reset(4)
    _swap_test(qc, 4, 8, 1, 'S7_swap_test')
    if input_state in {'+', '-'}:
        qc.h(8)
    qc.measure(0, 0)
    qc.measure(4, 1)
    qc.measure(8, 2)
    qc.metadata = {'candidate_type': 'faithful_v7_three_explicit_bell_resources', 'input_state': input_state,
'valid_expected_outputs': ['100'] if input_state in {'1', '-'} else ['000'], 'measurement_order': 'out[2] out[1] out[0]',
'success_definition': 'sum over valid expected outputs', 'paper_resource_qubits': 9, 'swap_ancillas': 'reuse of
consumed/dead measured-stage wires', 'not_implemented': ['QKD', 'authentication', 'network separation', 'abstract
arbitration']}
    return qc

def build_faithful_v7_unitary_reference(input_state='0'):
    qc = QuantumCircuit(11, name='faithful_v7_unitary_reference')
    _prepare_message_copies(qc, (0, 1, 2), input_state)
    for a, b in ((3, 4), (5, 6), (7, 8)):
        qc.h(a)
        qc.cx(a, b)
    coherent_teleportation(qc, 0, 3, 4, 'S2')
    append_encrypted_record_roundtrip(qc, 3, FIXED_KEY, '01', 'S3_X1_record')
    coherent_teleportation(qc, 4, 5, 6, 'S4')
    append_encrypted_record_roundtrip(qc, 5, FIXED_KEY, '10', 'S4_X2_record')
    _swap_test(qc, 9, 6, 2, 'S5_swap_test')
    coherent_teleportation(qc, 6, 7, 8, 'S6')
    append_encrypted_record_roundtrip(qc, 7, FIXED_KEY, '11', 'S6_X3_record')
    _swap_test(qc, 10, 8, 1, 'S7_swap_test')
    if input_state in {'+', '-'}:
        qc.h(8)
    return qc

def build_optimized_staged_candidate(input_state='0'):
    q = QuantumRegister(7, 'opt')
    c = ClassicalRegister(3, 'out')
    qc = QuantumCircuit(q, c, name='optimized_v7_staged_candidate')
    _prepare_message_copies(qc, (0, 1, 2), input_state)
    qc.barrier(label='S1')
```

```
    def leg(label):
        qc.h(3)
        qc.cx(3, 4)
        coherent_teleportation(qc, 0, 3, 4, label)
        qc.swap(0, 4)
        qc.reset(3)
        qc.reset(4)
    leg('S2')
    append_encrypted_record_roundtrip(qc, 6, FIXED_KEY, '01', 'S3_record')
    leg('S4')
    qc.reset(5)
    _swap_test(qc, 5, 0, 2, 'S5_swap_test')
    leg('S6')
    qc.reset(4)
    _swap_test(qc, 4, 0, 1, 'S7_swap_test')
    if input_state in {'+', '-'}:
        qc.h(0)
    qc.measure(5, 0)
    qc.measure(4, 1)
    qc.measure(0, 2)
    qc.metadata = {'candidate_type': 'optimized_staged_reset_reuse', 'input_state': input_state, 'valid_expected_outputs':
['100'] if input_state in {'1', '-'} else ['000'], 'measurement_order': 'out[2] out[1] out[0]', 'success_definition':
'sum over valid expected outputs', 'not_paper_resource_count': True}
    return qc
import csv
import matplotlib.pyplot as plt
from qiskit import qasm3, transpile
from qiskit.qasm3 import dumps as qasm3_dumps

from qiskit_aer import AerSimulator

# Self-contained matrix definitions used by the strengthened-QOTP circuit.
import numpy as np

X_MATRIX = np.array([[0, 1], [1, 0]], dtype=complex)
Y_MATRIX = np.array([[0, -1j], [1j, 0]], dtype=complex)
Z_MATRIX = np.array([[1, 0], [0, -1]], dtype=complex)
T_STRENGTHENED = np.sqrt(1j / 3) * (X_MATRIX - Y_MATRIX + Z_MATRIX)


def encryption_unitary(key4):
    """Return the one-qubit strengthened-QOTP unitary for a four-bit key block."""
    if len(key4) != 4 or set(key4) - {"0", "1"}:
        raise ValueError("key block must contain four binary digits")
    a, b, c, d = map(int, key4)
    return (
        np.linalg.matrix_power(X_MATRIX, a)
        @ np.linalg.matrix_power(Z_MATRIX, b)
        @ T_STRENGTHENED
        @ np.linalg.matrix_power(X_MATRIX, c)
        @ np.linalg.matrix_power(Z_MATRIX, d)
    )


# Circuit collection used for the modular and complete folders.
def build_swap_test_verification_circuit(left="0", right="1"):
    qc = QuantumCircuit(3, 1, name="swap_test_verification")
    if left == "1":
        qc.x(1)
    elif left == "+":
        qc.h(1)
    elif left == "-":
        qc.x(1); qc.h(1)
    if right == "1":
        qc.x(2)
    elif right == "+":
        qc.h(2)
    elif right == "-":
        qc.x(2); qc.h(2)
    qc.h(0); qc.cswap(0, 1, 2); qc.h(0); qc.measure(0, 0)
    return qc


def build_swap_test_circuit(left="0", right="1", name="swap_test"):
    """Compatibility wrapper used by the repeated swap-test circuit builder."""
```

```
    circuit = build_swap_test_verification_circuit(left, right)
    circuit.name = name
    return circuit


def circuit_collection():
    staged = build_staged_components()
    modular = {
        "Bell_Pair": build_bell_pair_circuit(),
        "Three_Bell_Pairs": build_three_bell_pair_circuit(),
        "Teleportation": build_teleportation_circuit(),
        "Three_Teleportation_Rounds": build_three_teleportation_rounds(),
        "Strengthened_QOTP_Roundtrip": build_qotp_roundtrip_circuit(),
        "Encrypted_Bell_Record": build_encrypted_bell_record_circuit(),
        "Swap_Test_Verification": build_swap_test_verification_circuit(),
        "Stage_S2": staged["S2"],
        "Stage_S3_Record": staged["S3_record"],
        "Stage_S5_QOTP": staged["S5_QOTP"],
        "Stage_S6": staged["S6"],
    }
    complete = {
        "Composed_Full_Candidate": build_composed_staged_emulation_candidate(),
        "Optimized_Staged_Candidate": build_optimized_staged_candidate(),
    }
    return modular, complete


def save_circuit(circuit, base_path):
    base_path.parent.mkdir(parents=True, exist_ok=True)
    (base_path.with_suffix(".qasm")).write_text(qasm3_dumps(circuit), encoding="utf-8")
    (base_path.with_suffix(".txt")).write_text(str(circuit.draw("text", fold=160)), encoding="utf-8")
    figure = circuit.draw("mpl", style="iqp", fold=80, idle_wires=False)
    for extension in ("pdf", "png", "svg"):
        figure.savefig(base_path.with_suffix("." + extension), dpi=300 if extension == "png" else None,
bbox_inches="tight", facecolor="white")
    plt.close(figure)

def generate_aer_circuits():
    root = Path(__file__).resolve().parent
    backend = AerSimulator()
    modular, complete = circuit_collection()
    rows = []
    for group, circuits in (("Modular_Circuits", modular), ("Complete_Circuits", complete)):
        for name, logical in circuits.items():
            aer_circuit = transpile(logical, backend=backend, optimization_level=1, seed_transpiler=20260803)
            save_circuit(aer_circuit, root / group / name)
            rows.append({"group": group, "circuit": name, "qubits": aer_circuit.num_qubits, "depth": aer_circuit.depth(),
"size": aer_circuit.size()})
    with (root / "Aer_Transpilation_Metrics.csv").open("w", newline="", encoding="utf-8") as handle:
        writer = csv.DictWriter(handle, fieldnames=list(rows[0])); writer.writeheader(); writer.writerows(rows)


if False and __name__ == "__main__":
    generate_aer_circuits()


# Aer execution, CSV generation, and result plotting
def run_aer_data(output_csv: Path, shots: int = 1024, seed: int = 20260803) -> None:
    backend = AerSimulator()
    modular, complete = circuit_collection()
    rows = []
    for group, circuits in (("modular", modular), ("complete", complete)):
        for name, logical in circuits.items():
            measured = logical.copy()
            if measured.num_clbits == 0:
                measured.measure_all()
            executable = transpile(measured, backend=backend, optimization_level=1, seed_transpiler=seed)
            counts = backend.run(executable, shots=shots, seed_simulator=seed).result().get_counts()
            valid_outputs = set(logical.metadata.get("valid_expected_outputs", ["0" * measured.num_clbits])) if
logical.metadata else {"0" * measured.num_clbits}
            correct = sum(int(value) for key, value in counts.items() if "".join(key.split()) in valid_outputs)
            rows.append({
                "group": group, "circuit": name, "shots": shots,
                "depth": executable.depth(), "size": executable.size(),
                "valid_expected_outputs": json.dumps(sorted(valid_outputs)),
```

```
                "correct_output_probability": correct / shots,
                "counts_json": json.dumps(counts, sort_keys=True),
            })
    output_csv.parent.mkdir(parents=True, exist_ok=True)
    with output_csv.open("w", newline="", encoding="utf-8") as handle:
        writer = csv.DictWriter(handle, fieldnames=list(rows[0])); writer.writeheader(); writer.writerows(rows)


def generate_aer_result_figure(input_csv: Path, output_path: Path) -> None:
    rows = list(csv.DictReader(input_csv.open(encoding="utf-8")))
    fig, ax = plt.subplots(figsize=(9.0, 4.8))
    ax.bar(range(len(rows)), [float(row["correct_output_probability"]) for row in rows], color="0.3")
    ax.set(xticks=range(len(rows)), xticklabels=[row["circuit"] for row in rows],
           ylabel="Correct-output probability", ylim=(0, 1.05), title="IBM Aer ideal execution")
    ax.tick_params(axis="x", rotation=65); fig.tight_layout()
    output_path.parent.mkdir(parents=True, exist_ok=True)
    fig.savefig(output_path.with_suffix(".png"), dpi=300); fig.savefig(output_path.with_suffix(".pdf")); plt.close(fig)


def run_complete_aer_pipeline(output_dir: Path, shots: int = 1024) -> None:
    generate_aer_circuits()
    data = output_dir / "aer_results.csv"
    run_aer_data(data, shots)
    generate_aer_result_figure(data, output_dir / "aer_results")


if __name__ == "__main__":
    parser = argparse.ArgumentParser(description="Generate Aer circuits, data, and result figures.")
    parser.add_argument("--output-dir", type=Path, default=Path("aer_results"))
    parser.add_argument("--shots", type=int, default=1024)
    arguments = parser.parse_args(); run_complete_aer_pipeline(arguments.output_dir, arguments.shots)
```

aer_verification_experiments.py

```python
"""Aer-specific entry point for the organized verification experiment suite.

The numerical implementation is imported from the shared Qiskit simulation module so
that the Aer wrapper does not duplicate or alter the validated computational logic.
Running this module writes only to the explicitly supplied data directory.
"""

from __future__ import annotations

import argparse
import sys
from pathlib import Path


QISKIT_MODULE_DIR = Path(__file__).resolve().parents[1] / "qiskit_simulation"
if str(QISKIT_MODULE_DIR) not in sys.path:
    sys.path.insert(0, str(QISKIT_MODULE_DIR))

from verification_experiment_suite import qiskit_aer_local_reference


def run_aer_verification_experiments(repo_root: Path, data_dir: Path) -> None:
    """Run the existing local BasicSimulator/Aer reference workflow."""
    qiskit_aer_local_reference(repo_root.resolve(), data_dir.resolve())


if __name__ == "__main__":
    parser = argparse.ArgumentParser(description="Run local Qiskit Aer verification experiments.")
    parser.add_argument("--repo-root", type=Path, required=True)
    parser.add_argument("--data-dir", type=Path, required=True)
    arguments = parser.parse_args()
    run_aer_verification_experiments(arguments.repo_root, arguments.data_dir)
```

IBM Hardware and Offline Processing

ibm_hardware_circuit_generation.py

```
"""Complete IBM/Qiskit circuit, retrieval, evaluation, and guarded-submission code."""

from __future__ import annotations

import argparse
import csv
import hashlib
import json
from math import sqrt
from pathlib import Path

from qiskit import ClassicalRegister, QuantumCircuit, QuantumRegister, transpile
from qiskit.qasm3 import dumps as qasm3_dumps
from qiskit.transpiler import generate_preset_pass_manager
try:
    from qiskit_ibm_runtime import QiskitRuntimeService, SamplerV2
except ImportError:  # Required only for explicitly selected IBM online modes.
    QiskitRuntimeService = None
    SamplerV2 = None


# 1. Bell resources

def build_bell_pair_circuit(name='bell_pair'):
    q = QuantumRegister(2, 'bell')
    qc = QuantumCircuit(q, name=name)
    qc.h(q[0])
    qc.cx(q[0], q[1])
    return qc

def build_three_bell_pair_circuit():
    q = QuantumRegister(6, 'bell')
    qc = QuantumCircuit(q, name='three_v7_bell_pairs')
    for a, b in ((0, 1), (2, 3), (4, 5)):
        qc.h(q[a])
        qc.cx(q[a], q[b])
    qc.metadata = {'pairs': {'AB_S2': [0, 1], 'BT_S4': [2, 3], 'TB_S6': [4, 5]}}
    return qc

# 2. Teleportation

def coherent_teleportation(qc, message, bell_sender, bell_receiver, label):
    qc.barrier(label=f'{label}_Bell_measurement')
    qc.cx(message, bell_sender)
    qc.h(message)
    qc.cx(bell_sender, bell_receiver)
    qc.cz(message, bell_receiver)

def build_teleportation_circuit(input_state='0'):
    qc = QuantumCircuit(3, 1, name=f'teleport_{input_state}')
    if input_state == '1':
        qc.x(0)
    elif input_state == '+':
        qc.h(0)
    elif input_state == '-':
        qc.x(0)
        qc.h(0)
    qc.h(1)
    qc.cx(1, 2)
    coherent_teleportation(qc, 0, 1, 2, 'S2')
    if input_state in {'+', '-'}:
        qc.h(2)
    qc.measure(2, 0)
    return qc

def build_three_teleportation_rounds(input_state='0'):
    qc = QuantumCircuit(7, 1, name='three_teleportation_rounds')
    if input_state == '1':
        qc.x(0)
    elif input_state == '+':
```

```python
        qc.h(0)
    elif input_state == '-':
        qc.x(0)
        qc.h(0)
    for a, b in ((1, 2), (3, 4), (5, 6)):
        qc.h(a)
        qc.cx(a, b)
    coherent_teleportation(qc, 0, 1, 2, 'S2')
    coherent_teleportation(qc, 2, 3, 4, 'S4')
    coherent_teleportation(qc, 4, 5, 6, 'S6')
    if input_state in {'+', '-'}:
        qc.h(6)
    qc.measure(6, 0)
    return qc

# 3. Strengthened-QOTP

def append_qotp(qc, qubit, key4, label='E_K'):
    U = encryption_unitary(key4)
    qc.unitary(U, [qubit], label=label)

def append_qotp_inverse(qc, qubit, key4, label='E_K^-1'):
    U = encryption_unitary(key4)
    qc.unitary(U.conj().T, [qubit], label=label)

def build_qotp_roundtrip_circuit(key4='0101', input_state='0'):
    qc = QuantumCircuit(1, 1, name='strengthened_qotp_roundtrip')
    if input_state == '1':
        qc.x(0)
    elif input_state == '+':
        qc.h(0)
    elif input_state == '-':
        qc.x(0)
        qc.h(0)
    append_qotp(qc, 0, key4)
    append_qotp_inverse(qc, 0, key4)
    if input_state in {'+', '-'}:
        qc.h(0)
    qc.measure(0, 0)
    return qc

# 4. Encrypted Bell records

def append_encrypted_record_roundtrip(qc, carrier, key4, record, label):
    if record[1] == '1':
        qc.x(carrier)
    if record[0] == '1':
        qc.z(carrier)
    append_qotp(qc, carrier, key4, f'{label}_encrypt')
    append_qotp_inverse(qc, carrier, key4, f'{label}_decrypt')
    if record[0] == '1':
        qc.z(carrier)
    if record[1] == '1':
        qc.x(carrier)

def build_encrypted_bell_record_circuit(key4='1010', record='01'):
    qc = QuantumCircuit(2, 2, name='encrypted_bell_record_roundtrip')
    qc.h(0)
    qc.cx(0, 1)
    append_encrypted_record_roundtrip(qc, 1, key4, record, 'E_KU_X')
    qc.cx(0, 1)
    qc.h(0)
    qc.measure([0, 1], [0, 1])
    return qc

# 5. Repeated swap tests

def build_repeated_swap_test_circuits(left='0', right='1', lam=8):
    return [build_swap_test_circuit(left, right, name=f'swap_{index + 1}_of_{lam}') for index in range(lam)]

# 6. Staged components

def build_staged_components():
    return {'S2': build_teleportation_circuit(), 'S3_record': build_encrypted_bell_record_circuit(), 'S5_QOTP':
build_qotp_roundtrip_circuit(), 'S6': build_teleportation_circuit()}
```

```
# 7. Composed staged-emulation candidate

FIXED_KEY = '0101'

def _prepare_message_copies(qc, qubits, input_state):
    for q in qubits:
        if input_state == '1':
            qc.x(q)
        elif input_state == '+':
            qc.h(q)
        elif input_state == '-':
            qc.x(q)
            qc.h(q)

def _swap_test(qc, anc, left, right, label):
    qc.barrier(label=label)
    qc.h(anc)
    qc.cswap(anc, left, right)
    qc.h(anc)

def build_composed_staged_emulation_candidate(input_state='0'):
    q = QuantumRegister(9, 'candidate')
    c = ClassicalRegister(3, 'out')
    qc = QuantumCircuit(q, c, name='composed_staged_emulation_candidate')
    _prepare_message_copies(qc, (0, 1, 2), input_state)
    qc.barrier(label='S1_three_message_copies')
    for a, b, label in ((3, 4, 'AB_S2'), (5, 6, 'BT_S4'), (7, 8, 'TB_S6')):
        qc.h(a)
        qc.cx(a, b)
        qc.barrier(label=f'I2_{label}')
    coherent_teleportation(qc, 0, 3, 4, 'S2')
    append_encrypted_record_roundtrip(qc, 3, FIXED_KEY, '01', 'S3_X1_record')
    coherent_teleportation(qc, 4, 5, 6, 'S4')
    append_encrypted_record_roundtrip(qc, 5, FIXED_KEY, '10', 'S4_X2_record')
    qc.reset(0)
    _swap_test(qc, 0, 6, 2, 'S5_swap_test')
    coherent_teleportation(qc, 6, 7, 8, 'S6')
    append_encrypted_record_roundtrip(qc, 7, FIXED_KEY, '11', 'S6_X3_record')
    qc.reset(4)
    _swap_test(qc, 4, 8, 1, 'S7_swap_test')
    if input_state in {'+', '-'}:
        qc.h(8)
    qc.measure(0, 0)
    qc.measure(4, 1)
    qc.measure(8, 2)
    qc.metadata = {'candidate_type': 'faithful_v7_three_explicit_bell_resources', 'input_state': input_state,
'valid_expected_outputs': ['100'] if input_state in {'1', '-'} else ['000'], 'measurement_order': 'out[2] out[1] out[0]',
'success_definition': 'sum over valid expected outputs', 'paper_resource_qubits': 9, 'swap_ancillas': 'reuse of
consumed/dead measured-stage wires', 'not_implemented': ['QKD', 'authentication', 'network separation', 'abstract
arbitration']}
    return qc

def build_faithful_v7_unitary_reference(input_state='0'):
    qc = QuantumCircuit(11, name='faithful_v7_unitary_reference')
    _prepare_message_copies(qc, (0, 1, 2), input_state)
    for a, b in ((3, 4), (5, 6), (7, 8)):
        qc.h(a)
        qc.cx(a, b)
    coherent_teleportation(qc, 0, 3, 4, 'S2')
    append_encrypted_record_roundtrip(qc, 3, FIXED_KEY, '01', 'S3_X1_record')
    coherent_teleportation(qc, 4, 5, 6, 'S4')
    append_encrypted_record_roundtrip(qc, 5, FIXED_KEY, '10', 'S4_X2_record')
    _swap_test(qc, 9, 6, 2, 'S5_swap_test')
    coherent_teleportation(qc, 6, 7, 8, 'S6')
    append_encrypted_record_roundtrip(qc, 7, FIXED_KEY, '11', 'S6_X3_record')
    _swap_test(qc, 10, 8, 1, 'S7_swap_test')
    if input_state in {'+', '-'}:
        qc.h(8)
    return qc

def build_optimized_staged_candidate(input_state='0'):
    q = QuantumRegister(7, 'opt')
    c = ClassicalRegister(3, 'out')
    qc = QuantumCircuit(q, c, name='optimized_v7_staged_candidate')
```

```
    _prepare_message_copies(qc, (0, 1, 2), input_state)
    qc.barrier(label='S1')

    def leg(label):
        qc.h(3)
        qc.cx(3, 4)
        coherent_teleportation(qc, 0, 3, 4, label)
        qc.swap(0, 4)
        qc.reset(3)
        qc.reset(4)
    leg('S2')
    append_encrypted_record_roundtrip(qc, 6, FIXED_KEY, '01', 'S3_record')
    leg('S4')
    qc.reset(5)
    _swap_test(qc, 5, 0, 2, 'S5_swap_test')
    leg('S6')
    qc.reset(4)
    _swap_test(qc, 4, 0, 1, 'S7_swap_test')
    if input_state in {'+', '-'}:
        qc.h(0)
    qc.measure(5, 0)
    qc.measure(4, 1)
    qc.measure(0, 2)
    qc.metadata = {'candidate_type': 'optimized_staged_reset_reuse', 'input_state': input_state, 'valid_expected_outputs':
['100'] if input_state in {'1', '-'} else ['000'], 'measurement_order': 'out[2] out[1] out[0]', 'success_definition':
'sum over valid expected outputs', 'not_paper_resource_count': True}
    return qc


# 8. IBM Runtime utilities
PACKAGE_ROOT = Path(__file__).resolve().parents[2]
JOB_INDEX = PACKAGE_ROOT / "data/ibm_hardware_existing/ibm_job_index.csv"


def runtime_service() -> QiskitRuntimeService:
    if QiskitRuntimeService is None:
        raise RuntimeError("qiskit-ibm-runtime is required only for explicitly selected IBM online modes")
    return QiskitRuntimeService(name="entropy-project")


def available_backends(service: QiskitRuntimeService):
    rows = []
    for backend in service.backends(operational=True, simulator=False):
        status = backend.status()
        rows.append({"backend": backend.name, "qubits": backend.num_qubits, "operational": status.operational,
"pending_jobs": status.pending_jobs})
    return sorted(rows, key=lambda row: (row["pending_jobs"], -row["qubits"]))


def select_backend(service: QiskitRuntimeService, name: str = "ibm_fez"):
    backend = service.backend(name)
    if getattr(backend.configuration(), "simulator", True):
        raise RuntimeError("A real QPU is required.")
    return backend


def transpile_for_backend(circuit: QuantumCircuit, backend, optimization_level: int = 2):
    pass_manager = generate_preset_pass_manager(backend=backend, optimization_level=optimization_level)
    return pass_manager.run(circuit)


def extract_counts(pub_result) -> dict[str, int]:
    for name in dir(pub_result.data):
        if name.startswith("_"):
            continue
        value = getattr(pub_result.data, name)
        if hasattr(value, "get_counts"):
            return dict(value.get_counts())
    raise RuntimeError("No classical result register with get_counts() was found.")


def wilson_interval(successes: int, shots: int, z: float = 1.959963984540054) -> tuple[float, float]:
    p = successes / shots
    denominator = 1 + z * z / shots
    center = (p + z * z / (2 * shots)) / denominator
```

```
    half = z * sqrt(p * (1 - p) / shots + z * z / (4 * shots * shots)) / denominator
    return center - half, center + half


def read_job_index(path: Path = JOB_INDEX) -> list[dict[str, str]]:
    with path.open(encoding="utf-8", newline="") as handle:
        return list(csv.DictReader(handle))


def retrieve_existing_jobs(output_csv: Path) -> None:
    service = runtime_service(); rows = []
    for record in read_job_index():
        job = service.job(record["job_id"]); result = job.result()
        rows.append({"job_id": record["job_id"], "backend": record["backend"], "shots": record["shots"], "status":
str(job.status()), "pub_count": len(result)})
    with output_csv.open("w", newline="", encoding="utf-8") as handle:
        writer = csv.DictWriter(handle, fieldnames=list(rows[0])); writer.writeheader(); writer.writerows(rows)


def submit_one_candidate(shots: int, backend_name: str, confirm: bool) -> str:
    if not confirm:
        raise RuntimeError("Submission requires both --submit and --confirm-submit.")
    service = runtime_service(); backend = select_backend(service, backend_name)
    circuit = build_composed_staged_emulation_candidate(); circuit.name = "composed_staged_emulation_candidate"
    isa = transpile_for_backend(circuit, backend, optimization_level=2)
    two_qubit = sum(count for name, count in isa.count_ops().items() if name in {"cz", "cx", "ecr", "rzz"})
    print(json.dumps({"backend": backend.name, "shots": shots, "qubits": isa.num_qubits, "depth": isa.depth(), "size":
isa.size(), "two_qubit_gates": two_qubit}, indent=2))
    sampler = SamplerV2(mode=backend); job = sampler.run([isa], shots=shots)
    print(job.job_id()); return job.job_id()


def save_probability(counts: dict[str, int], expected: str, output_csv: Path) -> None:
    shots = sum(counts.values()); success = counts.get(expected, 0); low, high = wilson_interval(success, shots)
    row = {"expected": expected, "shots": shots, "success_count": success, "correct_output_probability": success / shots,
"wilson_95_low": low, "wilson_95_high": high, "counts_json": json.dumps(counts, sort_keys=True)}
    with output_csv.open("w", newline="", encoding="utf-8") as handle:
        writer = csv.DictWriter(handle, fieldnames=list(row)); writer.writeheader(); writer.writerow(row)


def main() -> None:
    parser = argparse.ArgumentParser(description="Process existing IBM job results or explicitly submit one circuit
candidate.")
    parser.add_argument("--mode", choices=["retrieve_existing_jobs", "list_backends"], default="retrieve_existing_jobs")
    parser.add_argument("--output", type=Path, default=Path("retrieved_jobs.csv"))
    parser.add_argument("--backend", default="ibm_fez"); parser.add_argument("--shots", type=int, default=128)
    parser.add_argument("--submit", action="store_true"); parser.add_argument("--confirm-submit", action="store_true")
    args = parser.parse_args()
    if args.submit:
        submit_one_candidate(args.shots, args.backend, args.confirm_submit)
    elif args.mode == "list_backends":
        print(json.dumps(available_backends(runtime_service()), indent=2))
    else:
        retrieve_existing_jobs(args.output); print(args.output)
import csv
import matplotlib.pyplot as plt
from qiskit import qasm3, transpile
from qiskit.qasm3 import dumps as qasm3_dumps

# Self-contained matrix definitions used by the strengthened-QOTP circuit.
import numpy as np

X_MATRIX = np.array([[0, 1], [1, 0]], dtype=complex)
Y_MATRIX = np.array([[0, -1j], [1j, 0]], dtype=complex)
Z_MATRIX = np.array([[1, 0], [0, -1]], dtype=complex)
T_STRENGTHENED = np.sqrt(1j / 3) * (X_MATRIX - Y_MATRIX + Z_MATRIX)


def encryption_unitary(key4):
    """Return the one-qubit strengthened-QOTP unitary for a four-bit key block."""
    if len(key4) != 4 or set(key4) - {"0", "1"}:
        raise ValueError("key block must contain four binary digits")
    a, b, c, d = map(int, key4)
    return (
        np.linalg.matrix_power(X_MATRIX, a)
```

```
        @ np.linalg.matrix_power(Z_MATRIX, b)
        @ T_STRENGTHENED
        @ np.linalg.matrix_power(X_MATRIX, c)
        @ np.linalg.matrix_power(Z_MATRIX, d)
    )


# Circuit collection used for the modular and complete folders.
def build_swap_test_verification_circuit(left="0", right="1"):
    qc = QuantumCircuit(3, 1, name="swap_test_verification")
    if left == "1":
        qc.x(1)
    elif left == "+":
        qc.h(1)
    elif left == "-":
        qc.x(1); qc.h(1)
    if right == "1":
        qc.x(2)
    elif right == "+":
        qc.h(2)
    elif right == "-":
        qc.x(2); qc.h(2)
    qc.h(0); qc.cswap(0, 1, 2); qc.h(0); qc.measure(0, 0)
    return qc


def build_swap_test_circuit(left="0", right="1", name="swap_test"):
    """Compatibility wrapper used by the repeated swap-test circuit builder."""
    circuit = build_swap_test_verification_circuit(left, right)
    circuit.name = name
    return circuit


def circuit_collection():
    staged = build_staged_components()
    modular = {
        "Bell_Pair": build_bell_pair_circuit(),
        "Three_Bell_Pairs": build_three_bell_pair_circuit(),
        "Teleportation": build_teleportation_circuit(),
        "Three_Teleportation_Rounds": build_three_teleportation_rounds(),
        "Strengthened_QOTP_Roundtrip": build_qotp_roundtrip_circuit(),
        "Encrypted_Bell_Record": build_encrypted_bell_record_circuit(),
        "Swap_Test_Verification": build_swap_test_verification_circuit(),
        "Stage_S2": staged["S2"],
        "Stage_S3_Record": staged["S3_record"],
        "Stage_S5_QOTP": staged["S5_QOTP"],
        "Stage_S6": staged["S6"],
    }
    complete = {
        "Composed_Full_Candidate": build_composed_staged_emulation_candidate(),
        "Optimized_Staged_Candidate": build_optimized_staged_candidate(),
    }
    return modular, complete


def save_circuit(circuit, base_path):
    base_path.parent.mkdir(parents=True, exist_ok=True)
    (base_path.with_suffix(".qasm")).write_text(qasm3_dumps(circuit), encoding="utf-8")
    (base_path.with_suffix(".txt")).write_text(str(circuit.draw("text", fold=160)), encoding="utf-8")
    figure = circuit.draw("mpl", style="iqp", fold=80, idle_wires=False)
    for extension in ("pdf", "png", "svg"):
        figure.savefig(base_path.with_suffix("." + extension), dpi=300 if extension == "png" else None,
bbox_inches="tight", facecolor="white")
    plt.close(figure)

def generate_ibm_target_circuits(backend_name="ibm_fez", *, connect_ibm=False):
    # This function connects only to obtain the backend target; it never submits a job.
    import os
    if not connect_ibm:
        raise RuntimeError("offline mode is the default; pass connect_ibm=True or --connect-ibm to query a backend
target")
    if QiskitRuntimeService is None:
        raise RuntimeError("qiskit-ibm-runtime is required only for explicitly selected IBM online modes")
    root = Path(__file__).resolve().parent
    try:
```

```python
        service = QiskitRuntimeService(name="entropy-project")
    except Exception:
        # Credentials remain in environment variables and are never written to output.
        service = QiskitRuntimeService(
            channel="ibm_quantum_platform",
            token=os.environ["QISKIT_IBM_TOKEN"],
            instance=os.environ["QISKIT_IBM_CRN"],
        )
    backend = service.backend(backend_name)
    if getattr(backend.configuration(), "simulator", True):
        raise RuntimeError("A real QPU target is required.")
    modular, complete = circuit_collection()
    rows = []
    for group, circuits in (("Modular_Circuits", modular), ("Complete_Circuits", complete)):
        for name, logical in circuits.items():
            pass_manager = generate_preset_pass_manager(backend=backend, optimization_level=1)
            isa = pass_manager.run(logical)
            save_circuit(isa, root / group / name)
            two_qubit = sum(count for gate, count in isa.count_ops().items() if gate in {"cz", "cx", "ecr", "rzz"})
            rows.append({
                "group": group, "circuit": name, "backend": backend.name,
                "simulator": False, "physical_qubits": isa.num_qubits,
                "depth": isa.depth(), "size": isa.size(), "two_qubit_gates": two_qubit,
            })
    with (root / "IBM_Target_Transpilation_Metrics.csv").open("w", newline="", encoding="utf-8") as handle:
        writer = csv.DictWriter(handle, fieldnames=list(rows[0])); writer.writeheader(); writer.writerows(rows)


if __name__ == "__main__":
    parser = argparse.ArgumentParser(description="Generate target-transpiled circuit diagrams only after explicit IBM
connection opt-in.")
    parser.add_argument("--backend", default="ibm_fez")
    parser.add_argument("--connect-ibm", action="store_true", help="Explicitly permit IBM account/backend access; never
submits a job.")
    args = parser.parse_args()
    generate_ibm_target_circuits(args.backend, connect_ibm=args.connect_ibm)
```

ibm_hardware_complete_pipeline.py

```
"""Complete IBM/Qiskit circuit, retrieval, evaluation, and guarded-submission code."""

from __future__ import annotations

import argparse
import csv
import hashlib
import json
from math import sqrt
from pathlib import Path

from qiskit import ClassicalRegister, QuantumCircuit, QuantumRegister, transpile
from qiskit.qasm3 import dumps as qasm3_dumps
from qiskit.transpiler import generate_preset_pass_manager
try:
    from qiskit_ibm_runtime import QiskitRuntimeService, SamplerV2
except ImportError:  # Required only for explicitly selected IBM online modes.
    QiskitRuntimeService = None
    SamplerV2 = None


# 1. Bell resources

def build_bell_pair_circuit(name='bell_pair'):
    q = QuantumRegister(2, 'bell')
    qc = QuantumCircuit(q, name=name)
    qc.h(q[0])
    qc.cx(q[0], q[1])
    return qc

def build_three_bell_pair_circuit():
    q = QuantumRegister(6, 'bell')
    qc = QuantumCircuit(q, name='three_v7_bell_pairs')
    for a, b in ((0, 1), (2, 3), (4, 5)):
        qc.h(q[a])
        qc.cx(q[a], q[b])
    qc.metadata = {'pairs': {'AB_S2': [0, 1], 'BT_S4': [2, 3], 'TB_S6': [4, 5]}}
    return qc

# 2. Teleportation

def coherent_teleportation(qc, message, bell_sender, bell_receiver, label):
    qc.barrier(label=f'{label}_Bell_measurement')
    qc.cx(message, bell_sender)
    qc.h(message)
    qc.cx(bell_sender, bell_receiver)
    qc.cz(message, bell_receiver)

def build_teleportation_circuit(input_state='0'):
    qc = QuantumCircuit(3, 1, name=f'teleport_{input_state}')
    if input_state == '1':
        qc.x(0)
    elif input_state == '+':
        qc.h(0)
    elif input_state == '-':
        qc.x(0)
        qc.h(0)
    qc.h(1)
    qc.cx(1, 2)
    coherent_teleportation(qc, 0, 1, 2, 'S2')
    if input_state in {'+', '-'}:
        qc.h(2)
    qc.measure(2, 0)
    return qc

def build_three_teleportation_rounds(input_state='0'):
    qc = QuantumCircuit(7, 1, name='three_teleportation_rounds')
    if input_state == '1':
        qc.x(0)
    elif input_state == '+':
        qc.h(0)
    elif input_state == '-':
        qc.x(0)
```

```
        qc.h(0)
    for a, b in ((1, 2), (3, 4), (5, 6)):
        qc.h(a)
        qc.cx(a, b)
    coherent_teleportation(qc, 0, 1, 2, 'S2')
    coherent_teleportation(qc, 2, 3, 4, 'S4')
    coherent_teleportation(qc, 4, 5, 6, 'S6')
    if input_state in {'+', '-'}:
        qc.h(6)
    qc.measure(6, 0)
    return qc

# 3. Strengthened-QOTP

def append_qotp(qc, qubit, key4, label='E_K'):
    U = encryption_unitary(key4)
    qc.unitary(U, [qubit], label=label)

def append_qotp_inverse(qc, qubit, key4, label='E_K^-1'):
    U = encryption_unitary(key4)
    qc.unitary(U.conj().T, [qubit], label=label)

def build_qotp_roundtrip_circuit(key4='0101', input_state='0'):
    qc = QuantumCircuit(1, 1, name='strengthened_qotp_roundtrip')
    if input_state == '1':
        qc.x(0)
    elif input_state == '+':
        qc.h(0)
    elif input_state == '-':
        qc.x(0)
        qc.h(0)
    append_qotp(qc, 0, key4)
    append_qotp_inverse(qc, 0, key4)
    if input_state in {'+', '-'}:
        qc.h(0)
    qc.measure(0, 0)
    return qc

# 4. Encrypted Bell records

def append_encrypted_record_roundtrip(qc, carrier, key4, record, label):
    if record[1] == '1':
        qc.x(carrier)
    if record[0] == '1':
        qc.z(carrier)
    append_qotp(qc, carrier, key4, f'{label}_encrypt')
    append_qotp_inverse(qc, carrier, key4, f'{label}_decrypt')
    if record[0] == '1':
        qc.z(carrier)
    if record[1] == '1':
        qc.x(carrier)

def build_encrypted_bell_record_circuit(key4='1010', record='01'):
    qc = QuantumCircuit(2, 2, name='encrypted_bell_record_roundtrip')
    qc.h(0)
    qc.cx(0, 1)
    append_encrypted_record_roundtrip(qc, 1, key4, record, 'E_KU_X')
    qc.cx(0, 1)
    qc.h(0)
    qc.measure([0, 1], [0, 1])
    return qc

# 5. Repeated swap tests

def build_repeated_swap_test_circuits(left='0', right='1', lam=8):
    return [build_swap_test_circuit(left, right, name=f'swap_{index + 1}_of_{lam}') for index in range(lam)]

# 6. Staged components

def build_staged_components():
    return {'S2': build_teleportation_circuit(), 'S3_record': build_encrypted_bell_record_circuit(), 'S5_QOTP':
build_qotp_roundtrip_circuit(), 'S6': build_teleportation_circuit()}

# 7. Composed staged-emulation candidate
```

```
FIXED_KEY = '0101'

def _prepare_message_copies(qc, qubits, input_state):
    for q in qubits:
        if input_state == '1':
            qc.x(q)
        elif input_state == '+':
            qc.h(q)
        elif input_state == '-':
            qc.x(q)
            qc.h(q)

def _swap_test(qc, anc, left, right, label):
    qc.barrier(label=label)
    qc.h(anc)
    qc.cswap(anc, left, right)
    qc.h(anc)

def build_composed_staged_emulation_candidate(input_state='0'):
    q = QuantumRegister(9, 'candidate')
    c = ClassicalRegister(3, 'out')
    qc = QuantumCircuit(q, c, name='composed_staged_emulation_candidate')
    _prepare_message_copies(qc, (0, 1, 2), input_state)
    qc.barrier(label='S1_three_message_copies')
    for a, b, label in ((3, 4, 'AB_S2'), (5, 6, 'BT_S4'), (7, 8, 'TB_S6')):
        qc.h(a)
        qc.cx(a, b)
        qc.barrier(label=f'I2_{label}')
    coherent_teleportation(qc, 0, 3, 4, 'S2')
    append_encrypted_record_roundtrip(qc, 3, FIXED_KEY, '01', 'S3_X1_record')
    coherent_teleportation(qc, 4, 5, 6, 'S4')
    append_encrypted_record_roundtrip(qc, 5, FIXED_KEY, '10', 'S4_X2_record')
    qc.reset(0)
    _swap_test(qc, 0, 6, 2, 'S5_swap_test')
    coherent_teleportation(qc, 6, 7, 8, 'S6')
    append_encrypted_record_roundtrip(qc, 7, FIXED_KEY, '11', 'S6_X3_record')
    qc.reset(4)
    _swap_test(qc, 4, 8, 1, 'S7_swap_test')
    if input_state in {'+', '-'}:
        qc.h(8)
    qc.measure(0, 0)
    qc.measure(4, 1)
    qc.measure(8, 2)
    qc.metadata = {'candidate_type': 'faithful_v7_three_explicit_bell_resources', 'input_state': input_state,
'valid_expected_outputs': ['100'] if input_state in {'1', '-'} else ['000'], 'measurement_order': 'out[2] out[1] out[0]',
'success_definition': 'sum over valid expected outputs', 'paper_resource_qubits': 9, 'swap_ancillas': 'reuse of
consumed/dead measured-stage wires', 'not_implemented': ['QKD', 'authentication', 'network separation', 'abstract
arbitration']}
    return qc

def build_faithful_v7_unitary_reference(input_state='0'):
    qc = QuantumCircuit(11, name='faithful_v7_unitary_reference')
    _prepare_message_copies(qc, (0, 1, 2), input_state)
    for a, b in ((3, 4), (5, 6), (7, 8)):
        qc.h(a)
        qc.cx(a, b)
    coherent_teleportation(qc, 0, 3, 4, 'S2')
    append_encrypted_record_roundtrip(qc, 3, FIXED_KEY, '01', 'S3_X1_record')
    coherent_teleportation(qc, 4, 5, 6, 'S4')
    append_encrypted_record_roundtrip(qc, 5, FIXED_KEY, '10', 'S4_X2_record')
    _swap_test(qc, 9, 6, 2, 'S5_swap_test')
    coherent_teleportation(qc, 6, 7, 8, 'S6')
    append_encrypted_record_roundtrip(qc, 7, FIXED_KEY, '11', 'S6_X3_record')
    _swap_test(qc, 10, 8, 1, 'S7_swap_test')
    if input_state in {'+', '-'}:
        qc.h(8)
    return qc

def build_optimized_staged_candidate(input_state='0'):
    q = QuantumRegister(7, 'opt')
    c = ClassicalRegister(3, 'out')
    qc = QuantumCircuit(q, c, name='optimized_v7_staged_candidate')
    _prepare_message_copies(qc, (0, 1, 2), input_state)
    qc.barrier(label='S1')
```

```
    def leg(label):
        qc.h(3)
        qc.cx(3, 4)
        coherent_teleportation(qc, 0, 3, 4, label)
        qc.swap(0, 4)
        qc.reset(3)
        qc.reset(4)
    leg('S2')
    append_encrypted_record_roundtrip(qc, 6, FIXED_KEY, '01', 'S3_record')
    leg('S4')
    qc.reset(5)
    _swap_test(qc, 5, 0, 2, 'S5_swap_test')
    leg('S6')
    qc.reset(4)
    _swap_test(qc, 4, 0, 1, 'S7_swap_test')
    if input_state in {'+', '-'}:
        qc.h(0)
    qc.measure(5, 0)
    qc.measure(4, 1)
    qc.measure(0, 2)
    qc.metadata = {'candidate_type': 'optimized_staged_reset_reuse', 'input_state': input_state, 'valid_expected_outputs':
['100'] if input_state in {'1', '-'} else ['000'], 'measurement_order': 'out[2] out[1] out[0]', 'success_definition':
'sum over valid expected outputs', 'not_paper_resource_count': True}
    return qc


# 8. IBM Runtime utilities
PACKAGE_ROOT = Path(__file__).resolve().parents[2]
JOB_INDEX = PACKAGE_ROOT / "data/ibm_hardware_existing/ibm_job_index.csv"


def runtime_service() -> QiskitRuntimeService:
    if QiskitRuntimeService is None:
        raise RuntimeError("qiskit-ibm-runtime is required only for explicitly selected IBM online modes")
    return QiskitRuntimeService(name="entropy-project")


def available_backends(service: QiskitRuntimeService):
    rows = []
    for backend in service.backends(operational=True, simulator=False):
        status = backend.status()
        rows.append({"backend": backend.name, "qubits": backend.num_qubits, "operational": status.operational,
"pending_jobs": status.pending_jobs})
    return sorted(rows, key=lambda row: (row["pending_jobs"], -row["qubits"]))


def select_backend(service: QiskitRuntimeService, name: str = "ibm_fez"):
    backend = service.backend(name)
    if getattr(backend.configuration(), "simulator", True):
        raise RuntimeError("A real QPU is required.")
    return backend


def transpile_for_backend(circuit: QuantumCircuit, backend, optimization_level: int = 2):
    pass_manager = generate_preset_pass_manager(backend=backend, optimization_level=optimization_level)
    return pass_manager.run(circuit)


def extract_counts(pub_result) -> dict[str, int]:
    for name in dir(pub_result.data):
        if name.startswith("_"):
            continue
        value = getattr(pub_result.data, name)
        if hasattr(value, "get_counts"):
            return dict(value.get_counts())
    raise RuntimeError("No classical result register with get_counts() was found.")


def wilson_interval(successes: int, shots: int, z: float = 1.959963984540054) -> tuple[float, float]:
    p = successes / shots
    denominator = 1 + z * z / shots
    center = (p + z * z / (2 * shots)) / denominator
    half = z * sqrt(p * (1 - p) / shots + z * z / (4 * shots * shots)) / denominator
    return center - half, center + half
```

```
def read_job_index(path: Path = JOB_INDEX) -> list[dict[str, str]]:
    with path.open(encoding="utf-8", newline="") as handle:
        return list(csv.DictReader(handle))


def retrieve_existing_jobs(output_csv: Path) -> None:
    service = runtime_service(); rows = []
    for record in read_job_index():
        job = service.job(record["job_id"]); result = job.result()
        rows.append({"job_id": record["job_id"], "backend": record["backend"], "shots": record["shots"], "status":
str(job.status()), "pub_count": len(result)})
    with output_csv.open("w", newline="", encoding="utf-8") as handle:
        writer = csv.DictWriter(handle, fieldnames=list(rows[0])); writer.writeheader(); writer.writerows(rows)


def submit_one_candidate(shots: int, backend_name: str, confirm: bool) -> str:
    if not confirm:
        raise RuntimeError("Submission requires both --submit and --confirm-submit.")
    service = runtime_service(); backend = select_backend(service, backend_name)
    circuit = build_composed_staged_emulation_candidate(); circuit.name = "composed_staged_emulation_candidate"
    isa = transpile_for_backend(circuit, backend, optimization_level=2)
    two_qubit = sum(count for name, count in isa.count_ops().items() if name in {"cz", "cx", "ecr", "rzz"})
    print(json.dumps({"backend": backend.name, "shots": shots, "qubits": isa.num_qubits, "depth": isa.depth(), "size":
isa.size(), "two_qubit_gates": two_qubit}, indent=2))
    sampler = SamplerV2(mode=backend); job = sampler.run([isa], shots=shots)
    print(job.job_id()); return job.job_id()


def save_probability(counts: dict[str, int], expected: str, output_csv: Path) -> None:
    shots = sum(counts.values()); success = counts.get(expected, 0); low, high = wilson_interval(success, shots)
    row = {"expected": expected, "shots": shots, "success_count": success, "correct_output_probability": success / shots,
"wilson_95_low": low, "wilson_95_high": high, "counts_json": json.dumps(counts, sort_keys=True)}
    with output_csv.open("w", newline="", encoding="utf-8") as handle:
        writer = csv.DictWriter(handle, fieldnames=list(row)); writer.writeheader(); writer.writerow(row)


def main() -> None:
    parser = argparse.ArgumentParser(description="Process existing IBM job results or explicitly submit one circuit
candidate.")
    parser.add_argument("--mode", choices=["retrieve_existing_jobs", "list_backends"], default="retrieve_existing_jobs")
    parser.add_argument("--output", type=Path, default=Path("retrieved_jobs.csv"))
    parser.add_argument("--backend", default="ibm_fez"); parser.add_argument("--shots", type=int, default=128)
    parser.add_argument("--submit", action="store_true"); parser.add_argument("--confirm-submit", action="store_true")
    args = parser.parse_args()
    if args.submit:
        submit_one_candidate(args.shots, args.backend, args.confirm_submit)
    elif args.mode == "list_backends":
        print(json.dumps(available_backends(runtime_service()), indent=2))
    else:
        retrieve_existing_jobs(args.output); print(args.output)
import csv
import matplotlib.pyplot as plt
from qiskit import qasm3, transpile
from qiskit.qasm3 import dumps as qasm3_dumps

# Self-contained matrix definitions used by the strengthened-QOTP circuit.
import numpy as np

X_MATRIX = np.array([[0, 1], [1, 0]], dtype=complex)
Y_MATRIX = np.array([[0, -1j], [1j, 0]], dtype=complex)
Z_MATRIX = np.array([[1, 0], [0, -1]], dtype=complex)
T_STRENGTHENED = np.sqrt(1j / 3) * (X_MATRIX - Y_MATRIX + Z_MATRIX)


def encryption_unitary(key4):
    """Return the one-qubit strengthened-QOTP unitary for a four-bit key block."""
    if len(key4) != 4 or set(key4) - {"0", "1"}:
        raise ValueError("key block must contain four binary digits")
    a, b, c, d = map(int, key4)
    return (
        np.linalg.matrix_power(X_MATRIX, a)
        @ np.linalg.matrix_power(Z_MATRIX, b)
        @ T_STRENGTHENED
        @ np.linalg.matrix_power(X_MATRIX, c)
```

```
        @ np.linalg.matrix_power(Z_MATRIX, d)
    )


# Circuit collection used for the modular and complete folders.
def build_swap_test_verification_circuit(left="0", right="1"):
    qc = QuantumCircuit(3, 1, name="swap_test_verification")
    if left == "1":
        qc.x(1)
    elif left == "+":
        qc.h(1)
    elif left == "-":
        qc.x(1); qc.h(1)
    if right == "1":
        qc.x(2)
    elif right == "+":
        qc.h(2)
    elif right == "-":
        qc.x(2); qc.h(2)
    qc.h(0); qc.cswap(0, 1, 2); qc.h(0); qc.measure(0, 0)
    return qc


def build_swap_test_circuit(left="0", right="1", name="swap_test"):
    """Compatibility wrapper used by the repeated swap-test circuit builder."""
    circuit = build_swap_test_verification_circuit(left, right)
    circuit.name = name
    return circuit


def circuit_collection():
    staged = build_staged_components()
    modular = {
        "Bell_Pair": build_bell_pair_circuit(),
        "Three_Bell_Pairs": build_three_bell_pair_circuit(),
        "Teleportation": build_teleportation_circuit(),
        "Three_Teleportation_Rounds": build_three_teleportation_rounds(),
        "Strengthened_QOTP_Roundtrip": build_qotp_roundtrip_circuit(),
        "Encrypted_Bell_Record": build_encrypted_bell_record_circuit(),
        "Swap_Test_Verification": build_swap_test_verification_circuit(),
        "Stage_S2": staged["S2"],
        "Stage_S3_Record": staged["S3_record"],
        "Stage_S5_QOTP": staged["S5_QOTP"],
        "Stage_S6": staged["S6"],
    }
    complete = {
        "Composed_Full_Candidate": build_composed_staged_emulation_candidate(),
        "Optimized_Staged_Candidate": build_optimized_staged_candidate(),
    }
    return modular, complete


def save_circuit(circuit, base_path):
    base_path.parent.mkdir(parents=True, exist_ok=True)
    (base_path.with_suffix(".qasm")).write_text(qasm3_dumps(circuit), encoding="utf-8")
    (base_path.with_suffix(".txt")).write_text(str(circuit.draw("text", fold=160)), encoding="utf-8")
    figure = circuit.draw("mpl", style="iqp", fold=80, idle_wires=False)
    for extension in ("pdf", "png", "svg"):
        figure.savefig(base_path.with_suffix("." + extension), dpi=300 if extension == "png" else None,
bbox_inches="tight", facecolor="white")
    plt.close(figure)

def generate_ibm_target_circuits(backend_name="ibm_fez", *, connect_ibm=False):
    # This function connects only to obtain the backend target; it never submits a job.
    import os
    if not connect_ibm:
        raise RuntimeError("offline mode is the default; pass connect_ibm=True or --connect-ibm to query a backend
target")
    if QiskitRuntimeService is None:
        raise RuntimeError("qiskit-ibm-runtime is required only for explicitly selected IBM online modes")
    root = Path(__file__).resolve().parent
    try:
        service = QiskitRuntimeService(name="entropy-project")
    except Exception:
        # Credentials remain in environment variables and are never written to output.
```

```
        service = QiskitRuntimeService(
            channel="ibm_quantum_platform",
            token=os.environ["QISKIT_IBM_TOKEN"],
            instance=os.environ["QISKIT_IBM_CRN"],
        )
    backend = service.backend(backend_name)
    if getattr(backend.configuration(), "simulator", True):
        raise RuntimeError("A real QPU target is required.")
    modular, complete = circuit_collection()
    rows = []
    for group, circuits in (("Modular_Circuits", modular), ("Complete_Circuits", complete)):
        for name, logical in circuits.items():
            pass_manager = generate_preset_pass_manager(backend=backend, optimization_level=1)
            isa = pass_manager.run(logical)
            save_circuit(isa, root / group / name)
            two_qubit = sum(count for gate, count in isa.count_ops().items() if gate in {"cz", "cx", "ecr", "rzz"})
            rows.append({
                "group": group, "circuit": name, "backend": backend.name,
                "simulator": False, "physical_qubits": isa.num_qubits,
                "depth": isa.depth(), "size": isa.size(), "two_qubit_gates": two_qubit,
            })
    with (root / "IBM_Target_Transpilation_Metrics.csv").open("w", newline="", encoding="utf-8") as handle:
        writer = csv.DictWriter(handle, fieldnames=list(rows[0])); writer.writeheader(); writer.writerows(rows)


if False and __name__ == "__main__":
    parser = argparse.ArgumentParser(description="Generate target-transpiled circuit diagrams without submitting a job.")
    parser.add_argument("--backend", default="ibm_fez")
    args = parser.parse_args()
    generate_ibm_target_circuits(args.backend)


# Existing IBM result-data processing and figure generation
import pandas as pd


IBM_DATA_ROOT = PACKAGE_ROOT / "data/ibm_hardware_existing"


def generate_ibm_hardware_figure(output_path: Path) -> None:
    """Plot included IBM results. This function does not connect to IBM or submit jobs."""
    teleportation = pd.read_csv(IBM_DATA_ROOT / "teleportation_hardware.csv")
    qotp = pd.read_csv(IBM_DATA_ROOT / "qotp_hardware.csv")
    swap = pd.read_csv(IBM_DATA_ROOT / "swap_test_hardware.csv")
    aer = pd.read_csv(IBM_DATA_ROOT / "composed_candidate_aer.csv").iloc[0]
    pilot = pd.read_csv(IBM_DATA_ROOT / "composed_candidate_pilot.csv").iloc[0]
    final = pd.read_csv(IBM_DATA_ROOT / "composed_candidate_final.csv").iloc[0]
    fig, axes = plt.subplots(2, 2, figsize=(12, 9))
    axes[0, 0].bar(teleportation["input_state"], teleportation["correct_output_probability"], color="0.35")
    axes[0, 0].set(title="(a) Quantum teleportation", ylabel="Correct-output probability", ylim=(0, 1.05))
    states = ["0", "1", "+", "-"]; positions = np.arange(4); width = 0.18
    keys = [str(value) for value in sorted(qotp["key"].unique())[:4]]
    for index, key in enumerate(keys):
        part = qotp[qotp["key"].astype(str) == key].set_index("input_state").reindex(states)
        axes[0, 1].bar(positions + (index - 1.5) * width, part["correct_output_probability"], width, label=f"K={key}")
    axes[0, 1].set_xticks(positions, states); axes[0, 1].set(title="(b) Strengthened-QOTP roundtrip", ylabel="Correct-
output probability", ylim=(0.94, 1.005)); axes[0, 1].legend(fontsize=8)
    cases = ["identical_0_0", "identical_plus_plus", "orthogonal_0_1", "orthogonal_plus_minus", "fixed_overlap_F_0.25"]
    selected = swap[(swap["execution"] == "Final") & (swap["lambda"] == 1)].set_index("case").loc[cases]
    ideal = (1 - selected["fidelity"].to_numpy()) / 2
    axes[1, 0].bar(np.arange(5) - 0.18, ideal, 0.36, label="Ideal", color="0.8", edgecolor="black")
    axes[1, 0].bar(np.arange(5) + 0.18, selected["single_test_detection_probability"], 0.36, label="IBM hardware",
color="0.25")
    axes[1, 0].set_xticks(range(5), ["0/0", "+/+", "0/1", "+/-", "F=.25"]); axes[1, 0].set(title="(c) Swap-test
verification", ylabel="Single-test detection probability", ylim=(0, 0.65)); axes[1, 0].legend(fontsize=8)
    values = [aer["success_probability"], pilot["success_probability"], final["success_probability"]]
    axes[1, 1].bar(["Aer ideal", "Pilot", "Final"], values, color=["0.75", "0.45", "0.15"])
    axes[1, 1].set(title="(d) Composed staged-emulation candidate", ylabel="Combined correct-output probability", ylim=(0,
1.05))
    fig.suptitle("IBM Quantum hardware results", fontsize=16); fig.tight_layout()
    output_path.parent.mkdir(parents=True, exist_ok=True)
    for extension in ("pdf", "png", "eps"):
        fig.savefig(output_path.with_suffix("." + extension), dpi=300, bbox_inches="tight")
    plt.close(fig)
```

```python
def main_complete_ibm_code() -> None:
    parser = argparse.ArgumentParser(description="Process existing IBM results; connection and submission require explicit flags.")
    parser.add_argument("--mode", choices=["plot-existing", "retrieve-existing", "list-backends", "generate-target-circuits"], default="plot-existing")
    parser.add_argument("--output", type=Path, default=Path("ibm_hardware_results"))
    parser.add_argument("--backend", default="ibm_fez"); parser.add_argument("--shots", type=int, default=128)
    parser.add_argument("--connect-ibm", action="store_true", help="Explicitly permit IBM account/backend access; never implies submission.")
    parser.add_argument("--submit", action="store_true"); parser.add_argument("--confirm-submit", action="store_true")
    args = parser.parse_args()
    if args.submit:
        submit_one_candidate(args.shots, args.backend, args.confirm_submit)
    elif args.mode == "retrieve-existing":
        retrieve_existing_jobs(args.output.with_suffix(".csv"))
    elif args.mode == "list-backends":
        print(json.dumps(available_backends(runtime_service()), indent=2))
    elif args.mode == "generate-target-circuits":
        generate_ibm_target_circuits(args.backend, connect_ibm=args.connect_ibm)
    else:
        generate_ibm_hardware_figure(args.output)


if __name__ == "__main__":
    main_complete_ibm_code()
```

ibm_hardware_result_analysis.py

```
"""Analysis entry point for existing IBM hardware result files.

This module has no IBM account connection and no job-submission function. It delegates
the existing CSV classification, Wilson-interval calculation, and Figure 7 rendering
logic to the shared verification suite. Output generation is guarded by an explicit
command-line flag to prevent accidental replacement of reviewed artifacts.
"""

from __future__ import annotations

import argparse
import sys
from pathlib import Path


QISKIT_MODULE_DIR = Path(__file__).resolve().parents[1] / "qiskit_simulation"
if str(QISKIT_MODULE_DIR) not in sys.path:
    sys.path.insert(0, str(QISKIT_MODULE_DIR))

from verification_experiment_suite import plot_figure6, wilson


def analyze_existing_hardware_results(repo_root: Path, data_dir: Path, figure_dir: Path) -> None:
    """Process included CSV files only; no IBM service call is made."""
    plot_figure6(repo_root.resolve(), data_dir.resolve(), figure_dir.resolve())


if __name__ == "__main__":
    parser = argparse.ArgumentParser(description="Analyze existing IBM hardware result CSV files.")
    parser.add_argument("--repo-root", type=Path, required=True)
    parser.add_argument("--data-dir", type=Path, required=True)
    parser.add_argument("--figure-dir", type=Path, required=True)
    parser.add_argument(
        "--write-derived-output",
        action="store_true",
        help="Required guard for writing classifications, confidence intervals, and Figure 7 files.",
    )
    arguments = parser.parse_args()
    if not arguments.write_derived_output:
        parser.error("no output written: pass --write-derived-output after selecting non-original target directories")
    analyze_existing_hardware_results(arguments.repo_root, arguments.data_dir, arguments.figure_dir)
```

ibm_hardware_result_pipeline.py

```
"""Complete IBM/Qiskit circuit, retrieval, evaluation, and guarded-submission code."""

from __future__ import annotations

import argparse
import csv
import hashlib
import json
from math import sqrt
from pathlib import Path

from qiskit import ClassicalRegister, QuantumCircuit, QuantumRegister, transpile
from qiskit.qasm3 import dumps as qasm3_dumps
from qiskit.transpiler import generate_preset_pass_manager
try:
    from qiskit_ibm_runtime import QiskitRuntimeService, SamplerV2
except ImportError:  # Offline plotting and circuit construction do not need Runtime.
    QiskitRuntimeService = None
    SamplerV2 = None


# 1. Bell resources

def build_bell_pair_circuit(name='bell_pair'):
    q = QuantumRegister(2, 'bell')
    qc = QuantumCircuit(q, name=name)
    qc.h(q[0])
    qc.cx(q[0], q[1])
    return qc

def build_three_bell_pair_circuit():
    q = QuantumRegister(6, 'bell')
    qc = QuantumCircuit(q, name='three_v7_bell_pairs')
    for a, b in ((0, 1), (2, 3), (4, 5)):
        qc.h(q[a])
        qc.cx(q[a], q[b])
    qc.metadata = {'pairs': {'AB_S2': [0, 1], 'BT_S4': [2, 3], 'TB_S6': [4, 5]}}
    return qc

# 2. Teleportation

def coherent_teleportation(qc, message, bell_sender, bell_receiver, label):
    qc.barrier(label=f'{label}_Bell_measurement')
    qc.cx(message, bell_sender)
    qc.h(message)
    qc.cx(bell_sender, bell_receiver)
    qc.cz(message, bell_receiver)

def build_teleportation_circuit(input_state='0'):
    qc = QuantumCircuit(3, 1, name=f'teleport_{input_state}')
    if input_state == '1':
        qc.x(0)
    elif input_state == '+':
        qc.h(0)
    elif input_state == '-':
        qc.x(0)
        qc.h(0)
    qc.h(1)
    qc.cx(1, 2)
    coherent_teleportation(qc, 0, 1, 2, 'S2')
    if input_state in {'+', '-'}:
        qc.h(2)
    qc.measure(2, 0)
    return qc

def build_three_teleportation_rounds(input_state='0'):
    qc = QuantumCircuit(7, 1, name='three_teleportation_rounds')
    if input_state == '1':
        qc.x(0)
    elif input_state == '+':
        qc.h(0)
    elif input_state == '-':
        qc.x(0)
```

```
        qc.h(0)
    for a, b in ((1, 2), (3, 4), (5, 6)):
        qc.h(a)
        qc.cx(a, b)
    coherent_teleportation(qc, 0, 1, 2, 'S2')
    coherent_teleportation(qc, 2, 3, 4, 'S4')
    coherent_teleportation(qc, 4, 5, 6, 'S6')
    if input_state in {'+', '-'}:
        qc.h(6)
    qc.measure(6, 0)
    return qc

# 3. Strengthened-QOTP

def append_qotp(qc, qubit, key4, label='E_K'):
    U = encryption_unitary(key4)
    qc.unitary(U, [qubit], label=label)

def append_qotp_inverse(qc, qubit, key4, label='E_K^-1'):
    U = encryption_unitary(key4)
    qc.unitary(U.conj().T, [qubit], label=label)

def build_qotp_roundtrip_circuit(key4='0101', input_state='0'):
    qc = QuantumCircuit(1, 1, name='strengthened_qotp_roundtrip')
    if input_state == '1':
        qc.x(0)
    elif input_state == '+':
        qc.h(0)
    elif input_state == '-':
        qc.x(0)
        qc.h(0)
    append_qotp(qc, 0, key4)
    append_qotp_inverse(qc, 0, key4)
    if input_state in {'+', '-'}:
        qc.h(0)
    qc.measure(0, 0)
    return qc

# 4. Encrypted Bell records

def append_encrypted_record_roundtrip(qc, carrier, key4, record, label):
    if record[1] == '1':
        qc.x(carrier)
    if record[0] == '1':
        qc.z(carrier)
    append_qotp(qc, carrier, key4, f'{label}_encrypt')
    append_qotp_inverse(qc, carrier, key4, f'{label}_decrypt')
    if record[0] == '1':
        qc.z(carrier)
    if record[1] == '1':
        qc.x(carrier)

def build_encrypted_bell_record_circuit(key4='1010', record='01'):
    qc = QuantumCircuit(2, 2, name='encrypted_bell_record_roundtrip')
    qc.h(0)
    qc.cx(0, 1)
    append_encrypted_record_roundtrip(qc, 1, key4, record, 'E_KU_X')
    qc.cx(0, 1)
    qc.h(0)
    qc.measure([0, 1], [0, 1])
    return qc

# 5. Repeated swap tests

def build_repeated_swap_test_circuits(left='0', right='1', lam=8):
    return [build_swap_test_circuit(left, right, name=f'swap_{index + 1}_of_{lam}') for index in range(lam)]

# 6. Staged components

def build_staged_components():
    return {'S2': build_teleportation_circuit(), 'S3_record': build_encrypted_bell_record_circuit(), 'S5_QOTP':
build_qotp_roundtrip_circuit(), 'S6': build_teleportation_circuit()}

# 7. Composed staged-emulation candidate
```

```python
FIXED_KEY = '0101'

def _prepare_message_copies(qc, qubits, input_state):
    for q in qubits:
        if input_state == '1':
            qc.x(q)
        elif input_state == '+':
            qc.h(q)
        elif input_state == '-':
            qc.x(q)
            qc.h(q)

def _swap_test(qc, anc, left, right, label):
    qc.barrier(label=label)
    qc.h(anc)
    qc.cswap(anc, left, right)
    qc.h(anc)

def build_composed_staged_emulation_candidate(input_state='0'):
    q = QuantumRegister(9, 'candidate')
    c = ClassicalRegister(3, 'out')
    qc = QuantumCircuit(q, c, name='composed_staged_emulation_candidate')
    _prepare_message_copies(qc, (0, 1, 2), input_state)
    qc.barrier(label='S1_three_message_copies')
    for a, b, label in ((3, 4, 'AB_S2'), (5, 6, 'BT_S4'), (7, 8, 'TB_S6')):
        qc.h(a)
        qc.cx(a, b)
        qc.barrier(label=f'I2_{label}')
    coherent_teleportation(qc, 0, 3, 4, 'S2')
    append_encrypted_record_roundtrip(qc, 3, FIXED_KEY, '01', 'S3_X1_record')
    coherent_teleportation(qc, 4, 5, 6, 'S4')
    append_encrypted_record_roundtrip(qc, 5, FIXED_KEY, '10', 'S4_X2_record')
    qc.reset(0)
    _swap_test(qc, 0, 6, 2, 'S5_swap_test')
    coherent_teleportation(qc, 6, 7, 8, 'S6')
    append_encrypted_record_roundtrip(qc, 7, FIXED_KEY, '11', 'S6_X3_record')
    qc.reset(4)
    _swap_test(qc, 4, 8, 1, 'S7_swap_test')
    if input_state in {'+', '-'}:
        qc.h(8)
    qc.measure(0, 0)
    qc.measure(4, 1)
    qc.measure(8, 2)
    qc.metadata = {'candidate_type': 'faithful_v7_three_explicit_bell_resources', 'input_state': input_state,
'valid_expected_outputs': ['100'] if input_state in {'1', '-'} else ['000'], 'measurement_order': 'out[2] out[1] out[0]',
'success_definition': 'sum over valid expected outputs', 'paper_resource_qubits': 9, 'swap_ancillas': 'reuse of
consumed/dead measured-stage wires', 'not_implemented': ['QKD', 'authentication', 'network separation', 'abstract
arbitration']}
    return qc

def build_faithful_v7_unitary_reference(input_state='0'):
    qc = QuantumCircuit(11, name='faithful_v7_unitary_reference')
    _prepare_message_copies(qc, (0, 1, 2), input_state)
    for a, b in ((3, 4), (5, 6), (7, 8)):
        qc.h(a)
        qc.cx(a, b)
    coherent_teleportation(qc, 0, 3, 4, 'S2')
    append_encrypted_record_roundtrip(qc, 3, FIXED_KEY, '01', 'S3_X1_record')
    coherent_teleportation(qc, 4, 5, 6, 'S4')
    append_encrypted_record_roundtrip(qc, 5, FIXED_KEY, '10', 'S4_X2_record')
    _swap_test(qc, 9, 6, 2, 'S5_swap_test')
    coherent_teleportation(qc, 6, 7, 8, 'S6')
    append_encrypted_record_roundtrip(qc, 7, FIXED_KEY, '11', 'S6_X3_record')
    _swap_test(qc, 10, 8, 1, 'S7_swap_test')
    if input_state in {'+', '-'}:
        qc.h(8)
    return qc

def build_optimized_staged_candidate(input_state='0'):
    q = QuantumRegister(7, 'opt')
    c = ClassicalRegister(3, 'out')
    qc = QuantumCircuit(q, c, name='optimized_v7_staged_candidate')
    _prepare_message_copies(qc, (0, 1, 2), input_state)
    qc.barrier(label='S1')
```

```
    def leg(label):
        qc.h(3)
        qc.cx(3, 4)
        coherent_teleportation(qc, 0, 3, 4, label)
        qc.swap(0, 4)
        qc.reset(3)
        qc.reset(4)
    leg('S2')
    append_encrypted_record_roundtrip(qc, 6, FIXED_KEY, '01', 'S3_record')
    leg('S4')
    qc.reset(5)
    _swap_test(qc, 5, 0, 2, 'S5_swap_test')
    leg('S6')
    qc.reset(4)
    _swap_test(qc, 4, 0, 1, 'S7_swap_test')
    if input_state in {'+', '-'}:
        qc.h(0)
    qc.measure(5, 0)
    qc.measure(4, 1)
    qc.measure(0, 2)
    qc.metadata = {'candidate_type': 'optimized_staged_reset_reuse', 'input_state': input_state, 'valid_expected_outputs':
['100'] if input_state in {'1', '-'} else ['000'], 'measurement_order': 'out[2] out[1] out[0]', 'success_definition':
'sum over valid expected outputs', 'not_paper_resource_count': True}
    return qc


# 8. IBM Runtime utilities
PACKAGE_ROOT = Path(__file__).resolve().parents[2]
JOB_INDEX = PACKAGE_ROOT / "data/ibm_hardware_existing/ibm_job_index.csv"


def runtime_service() -> QiskitRuntimeService:
    if QiskitRuntimeService is None:
        raise RuntimeError('qiskit-ibm-runtime is required only for online retrieval/submission modes')
    return QiskitRuntimeService(name="entropy-project")


def available_backends(service: QiskitRuntimeService):
    rows = []
    for backend in service.backends(operational=True, simulator=False):
        status = backend.status()
        rows.append({"backend": backend.name, "qubits": backend.num_qubits, "operational": status.operational,
"pending_jobs": status.pending_jobs})
    return sorted(rows, key=lambda row: (row["pending_jobs"], -row["qubits"]))


def select_backend(service: QiskitRuntimeService, name: str = "ibm_fez"):
    backend = service.backend(name)
    if getattr(backend.configuration(), "simulator", True):
        raise RuntimeError("A real QPU is required.")
    return backend


def transpile_for_backend(circuit: QuantumCircuit, backend, optimization_level: int = 2):
    pass_manager = generate_preset_pass_manager(backend=backend, optimization_level=optimization_level)
    return pass_manager.run(circuit)


def extract_counts(pub_result) -> dict[str, int]:
    for name in dir(pub_result.data):
        if name.startswith("_"):
            continue
        value = getattr(pub_result.data, name)
        if hasattr(value, "get_counts"):
            return dict(value.get_counts())
    raise RuntimeError("No classical result register with get_counts() was found.")


def wilson_interval(successes: int, shots: int, z: float = 1.959963984540054) -> tuple[float, float]:
    p = successes / shots
    denominator = 1 + z * z / shots
    center = (p + z * z / (2 * shots)) / denominator
    half = z * sqrt(p * (1 - p) / shots + z * z / (4 * shots * shots)) / denominator
    return center - half, center + half
```

```python
def read_job_index(path: Path = JOB_INDEX) -> list[dict[str, str]]:
    with path.open(encoding="utf-8", newline="") as handle:
        return list(csv.DictReader(handle))


def retrieve_existing_jobs(output_csv: Path) -> None:
    service = runtime_service(); rows = []
    for record in read_job_index():
        job = service.job(record["job_id"]); result = job.result()
        rows.append({"job_id": record["job_id"], "backend": record["backend"], "shots": record["shots"], "status":
str(job.status()), "pub_count": len(result)})
    with output_csv.open("w", newline="", encoding="utf-8") as handle:
        writer = csv.DictWriter(handle, fieldnames=list(rows[0])); writer.writeheader(); writer.writerows(rows)


def submit_one_candidate(shots: int, backend_name: str, confirm: bool) -> str:
    if not confirm:
        raise RuntimeError("Submission requires both --submit and --confirm-submit.")
    service = runtime_service(); backend = select_backend(service, backend_name)
    circuit = build_composed_staged_emulation_candidate(); circuit.name = "composed_staged_emulation_candidate"
    isa = transpile_for_backend(circuit, backend, optimization_level=2)
    two_qubit = sum(count for name, count in isa.count_ops().items() if name in {"cz", "cx", "ecr", "rzz"})
    print(json.dumps({"backend": backend.name, "shots": shots, "qubits": isa.num_qubits, "depth": isa.depth(), "size":
isa.size(), "two_qubit_gates": two_qubit}, indent=2))
    sampler = SamplerV2(mode=backend); job = sampler.run([isa], shots=shots)
    print(job.job_id()); return job.job_id()


def save_probability(counts: dict[str, int], expected: str, output_csv: Path) -> None:
    shots = sum(counts.values()); success = counts.get(expected, 0); low, high = wilson_interval(success, shots)
    row = {"expected": expected, "shots": shots, "success_count": success, "correct_output_probability": success / shots,
"wilson_95_low": low, "wilson_95_high": high, "counts_json": json.dumps(counts, sort_keys=True)}
    with output_csv.open("w", newline="", encoding="utf-8") as handle:
        writer = csv.DictWriter(handle, fieldnames=list(row)); writer.writeheader(); writer.writerow(row)


def main() -> None:
    parser = argparse.ArgumentParser(description="Process existing IBM job results or explicitly submit one circuit
candidate.")
    parser.add_argument("--mode", choices=["retrieve_existing_jobs", "list_backends"], default="retrieve_existing_jobs")
    parser.add_argument("--output", type=Path, default=Path("retrieved_jobs.csv"))
    parser.add_argument("--backend", default="ibm_fez"); parser.add_argument("--shots", type=int, default=128)
    parser.add_argument("--submit", action="store_true"); parser.add_argument("--confirm-submit", action="store_true")
    args = parser.parse_args()
    if args.submit:
        submit_one_candidate(args.shots, args.backend, args.confirm_submit)
    elif args.mode == "list_backends":
        print(json.dumps(available_backends(runtime_service()), indent=2))
    else:
        retrieve_existing_jobs(args.output); print(args.output)
import csv
import matplotlib.pyplot as plt
from qiskit import qasm3, transpile
from qiskit.qasm3 import dumps as qasm3_dumps

# Self-contained matrix definitions used by the strengthened-QOTP circuit.
import numpy as np

X_MATRIX = np.array([[0, 1], [1, 0]], dtype=complex)
Y_MATRIX = np.array([[0, -1j], [1j, 0]], dtype=complex)
Z_MATRIX = np.array([[1, 0], [0, -1]], dtype=complex)
T_STRENGTHENED = np.sqrt(1j / 3) * (X_MATRIX - Y_MATRIX + Z_MATRIX)


def encryption_unitary(key4):
    """Return the one-qubit strengthened-QOTP unitary for a four-bit key block."""
    if len(key4) != 4 or set(key4) - {"0", "1"}:
        raise ValueError("key block must contain four binary digits")
    a, b, c, d = map(int, key4)
    return (
        np.linalg.matrix_power(X_MATRIX, a)
        @ np.linalg.matrix_power(Z_MATRIX, b)
        @ T_STRENGTHENED
        @ np.linalg.matrix_power(X_MATRIX, c)
```

```
        @ np.linalg.matrix_power(Z_MATRIX, d)
    )


# Circuit collection used for the modular and complete folders.
def build_swap_test_verification_circuit(left="0", right="1"):
    qc = QuantumCircuit(3, 1, name="swap_test_verification")
    if left == "1":
        qc.x(1)
    elif left == "+":
        qc.h(1)
    elif left == "-":
        qc.x(1); qc.h(1)
    if right == "1":
        qc.x(2)
    elif right == "+":
        qc.h(2)
    elif right == "-":
        qc.x(2); qc.h(2)
    qc.h(0); qc.cswap(0, 1, 2); qc.h(0); qc.measure(0, 0)
    return qc


def build_swap_test_circuit(left="0", right="1", name="swap_test"):
    """Compatibility wrapper used by the repeated swap-test circuit builder."""
    circuit = build_swap_test_verification_circuit(left, right)
    circuit.name = name
    return circuit


def circuit_collection():
    staged = build_staged_components()
    modular = {
        "Bell_Pair": build_bell_pair_circuit(),
        "Three_Bell_Pairs": build_three_bell_pair_circuit(),
        "Teleportation": build_teleportation_circuit(),
        "Three_Teleportation_Rounds": build_three_teleportation_rounds(),
        "Strengthened_QOTP_Roundtrip": build_qotp_roundtrip_circuit(),
        "Encrypted_Bell_Record": build_encrypted_bell_record_circuit(),
        "Swap_Test_Verification": build_swap_test_verification_circuit(),
        "Stage_S2": staged["S2"],
        "Stage_S3_Record": staged["S3_record"],
        "Stage_S5_QOTP": staged["S5_QOTP"],
        "Stage_S6": staged["S6"],
    }
    complete = {
        "Composed_Full_Candidate": build_composed_staged_emulation_candidate(),
        "Optimized_Staged_Candidate": build_optimized_staged_candidate(),
    }
    return modular, complete


def save_circuit(circuit, base_path):
    base_path.parent.mkdir(parents=True, exist_ok=True)
    (base_path.with_suffix(".qasm")).write_text(qasm3_dumps(circuit), encoding="utf-8")
    (base_path.with_suffix(".txt")).write_text(str(circuit.draw("text", fold=160)), encoding="utf-8")
    figure = circuit.draw("mpl", style="iqp", fold=80, idle_wires=False)
    for extension in ("pdf", "png", "svg"):
        figure.savefig(base_path.with_suffix("." + extension), dpi=300 if extension == "png" else None,
bbox_inches="tight", facecolor="white")
    plt.close(figure)

def generate_ibm_target_circuits(backend_name="ibm_fez", *, connect_ibm=False):
    # This function connects only to obtain the backend target; it never submits a job.
    import os
    if not connect_ibm:
        raise RuntimeError("offline mode is the default; pass connect_ibm=True or --connect-ibm to query a backend
target")
    if QiskitRuntimeService is None:
        raise RuntimeError("qiskit-ibm-runtime is required only for explicitly selected IBM online modes")
    root = Path(__file__).resolve().parent
    try:
        service = QiskitRuntimeService(name="entropy-project")
    except Exception:
        # Credentials remain in environment variables and are never written to output.
```

```
        service = QiskitRuntimeService(
            channel="ibm_quantum_platform",
            token=os.environ["QISKIT_IBM_TOKEN"],
            instance=os.environ["QISKIT_IBM_CRN"],
        )
    backend = service.backend(backend_name)
    if getattr(backend.configuration(), "simulator", True):
        raise RuntimeError("A real QPU target is required.")
    modular, complete = circuit_collection()
    rows = []
    for group, circuits in (("Modular_Circuits", modular), ("Complete_Circuits", complete)):
        for name, logical in circuits.items():
            pass_manager = generate_preset_pass_manager(backend=backend, optimization_level=1)
            isa = pass_manager.run(logical)
            save_circuit(isa, root / group / name)
            two_qubit = sum(count for gate, count in isa.count_ops().items() if gate in {"cz", "cx", "ecr", "rzz"})
            rows.append({
                "group": group, "circuit": name, "backend": backend.name,
                "simulator": False, "physical_qubits": isa.num_qubits,
                "depth": isa.depth(), "size": isa.size(), "two_qubit_gates": two_qubit,
            })
    with (root / "IBM_Target_Transpilation_Metrics.csv").open("w", newline="", encoding="utf-8") as handle:
        writer = csv.DictWriter(handle, fieldnames=list(rows[0])); writer.writeheader(); writer.writerows(rows)


if False and __name__ == "__main__":
    parser = argparse.ArgumentParser(description="Generate target-transpiled circuit diagrams without submitting a job.")
    parser.add_argument("--backend", default="ibm_fez")
    args = parser.parse_args()
    generate_ibm_target_circuits(args.backend)


# Existing IBM result-data processing and figure generation
import pandas as pd


IBM_DATA_ROOT = PACKAGE_ROOT / "data/ibm_hardware_existing"


def generate_ibm_hardware_figure(output_path: Path) -> None:
    """Plot included IBM results. This function does not connect to IBM or submit jobs."""
    teleportation = pd.read_csv(IBM_DATA_ROOT / "teleportation_hardware.csv")
    qotp = pd.read_csv(IBM_DATA_ROOT / "qotp_hardware.csv")
    swap = pd.read_csv(IBM_DATA_ROOT / "swap_test_hardware.csv")
    aer = pd.read_csv(IBM_DATA_ROOT / "composed_candidate_aer.csv").iloc[0]
    pilot = pd.read_csv(IBM_DATA_ROOT / "composed_candidate_pilot.csv").iloc[0]
    final = pd.read_csv(IBM_DATA_ROOT / "composed_candidate_final.csv").iloc[0]
    fig, axes = plt.subplots(2, 2, figsize=(12, 9))
    axes[0, 0].bar(teleportation["input_state"], teleportation["correct_output_probability"], color="0.35")
    axes[0, 0].set(title="(a) Quantum teleportation", ylabel="Correct-output probability", ylim=(0, 1.05))
    states = ["0", "1", "+", "-"]; positions = np.arange(4); width = 0.18
    keys = sorted(qotp["key"].unique())[:4]
    for index, key in enumerate(keys):
        part = qotp[qotp["key"] == key].set_index("input_state").reindex(states)
        axes[0, 1].bar(positions + (index - 1.5) * width, part["correct_output_probability"], width,
label=f"K={int(key):04d}")
    axes[0, 1].set_xticks(positions, states); axes[0, 1].set(title="(b) Strengthened-QOTP roundtrip", ylabel="Correct-
output probability", ylim=(0.94, 1.005)); axes[0, 1].legend(fontsize=8)
    cases = ["identical_0_0", "identical_plus_plus", "orthogonal_0_1", "orthogonal_plus_minus", "fixed_overlap_F_0.25"]
    selected = swap[(swap["execution"] == "Final") & (swap["lambda"] == 1)].set_index("case").loc[cases]
    ideal = (1 - selected["fidelity"].to_numpy()) / 2
    axes[1, 0].bar(np.arange(5) - 0.18, ideal, 0.36, label="Ideal", color="0.8", edgecolor="black")
    axes[1, 0].bar(np.arange(5) + 0.18, selected["single_test_detection_probability"], 0.36, label="IBM hardware",
color="0.25")
    axes[1, 0].set_xticks(range(5), ["0/0", "+/+", "0/1", "+/-", "F=.25"]); axes[1, 0].set(title="(c) Swap-test
verification", ylabel="Single-test detection probability", ylim=(0, 0.65)); axes[1, 0].legend(fontsize=8)
    values = [aer["success_probability"], pilot["success_probability"], final["success_probability"]]
    axes[1, 1].bar(["Aer ideal", "Pilot", "Final"], values, color=["0.75", "0.45", "0.15"])
    axes[1, 1].set(title="(d) Composed staged-emulation candidate", ylabel="Combined correct-output probability", ylim=(0,
1.05))
    fig.suptitle("IBM Quantum hardware results and local Aer reference", fontsize=16); fig.tight_layout()
    output_path.parent.mkdir(parents=True, exist_ok=True)
    for extension in ("pdf", "png", "eps"):
        fig.savefig(output_path.with_suffix("." + extension), dpi=300, bbox_inches="tight")
    plt.close(fig)
```

```python
def main_complete_ibm_code() -> None:
    parser = argparse.ArgumentParser(description="Process existing IBM results; connection and submission require explicit flags.")
    parser.add_argument("--mode", choices=["plot-existing", "retrieve-existing", "list-backends", "generate-target-circuits"], default="plot-existing")
    parser.add_argument("--output", type=Path, default=Path("ibm_hardware_results"))
    parser.add_argument("--backend", default="ibm_fez"); parser.add_argument("--shots", type=int, default=128)
    parser.add_argument("--connect-ibm", action="store_true", help="Explicitly permit IBM account/backend access; never implies submission.")
    parser.add_argument("--submit", action="store_true"); parser.add_argument("--confirm-submit", action="store_true")
    args = parser.parse_args()
    if args.submit:
        submit_one_candidate(args.shots, args.backend, args.confirm_submit)
    elif args.mode == "retrieve-existing":
        retrieve_existing_jobs(args.output.with_suffix(".csv"))
    elif args.mode == "list-backends":
        print(json.dumps(available_backends(runtime_service()), indent=2))
    elif args.mode == "generate-target-circuits":
        generate_ibm_target_circuits(args.backend, connect_ibm=args.connect_ibm)
    else:
        generate_ibm_hardware_figure(args.output)


if __name__ == "__main__":
    main_complete_ibm_code()
```

ibm_offline_analysis.py

```python
"""Offline-only replay of immutable IBM result files.

This module has no IBM Runtime import and cannot submit or retrieve a job.
"""

from __future__ import annotations

import csv
import json
from math import sqrt
from pathlib import Path

import pandas as pd


PACKAGE_ROOT = Path(__file__).resolve().parents[2]
DEFAULT_IBM_DATA_ROOT = PACKAGE_ROOT / "data/ibm_hardware_existing"


def wilson_interval(successes: int, shots: int, z: float = 1.959963984540054) -> tuple[float, float]:
    if shots <= 0 or successes < 0 or successes > shots:
        raise ValueError("require 0 <= successes <= shots and shots > 0")
    p = successes / shots
    denominator = 1 + z * z / shots
    center = (p + z * z / (2 * shots)) / denominator
    half = z * sqrt(p * (1 - p) / shots + z * z / (4 * shots * shots)) / denominator
    return center - half, center + half


def analyze_existing_hardware(output_path: Path, input_root: Path = DEFAULT_IBM_DATA_ROOT) -> None:
    """Recompute direct and derived metrics without constructing an IBM service."""
    input_root = input_root.resolve()
    final = pd.read_csv(input_root / "composed_candidate_final.csv").iloc[0]
    raw_counts = json.loads(final["counts_json"])
    shots = int(sum(raw_counts.values()))
    expected = f"{int(final['expected']):03d}"
    success_count = int(raw_counts.get(expected, 0))
    low, high = wilson_interval(success_count, shots)
    rows = [
        {
            "record_type": "composed_candidate_final",
            "execution": final["execution"],
            "case": "composed_candidate_m1",
            "m": 1,
            "DIRECT_HARDWARE": "YES",
            "DERIVED_FROM_SINGLE_TEST": "NO",
            "backend": final["backend"],
            "job_id": final["job_id"],
            "shots": shots,
            "recomputed_accept_probability": success_count / shots,
            "recomputed_detection_probability": "",
            "recomputed_wilson_95_low": low,
            "recomputed_wilson_95_high": high,
            "stored_probability": float(final["success_probability"]),
            "absolute_difference": abs(success_count / shots - float(final["success_probability"])),
            "composition_assumption": "direct m=1 hardware counts",
        }
    ]

    swap = pd.read_csv(input_root / "swap_test_hardware.csv")
    for (execution, case), group in swap.groupby(["execution", "case"], sort=False):
        direct = group[group["lambda"] == 1].iloc[0]
        single_accept = float(direct["single_test_accept_probability"])
        single_detection = 1.0 - single_accept
        detection_count = int(round(single_detection * int(direct["shots"])))
        direct_low, direct_high = wilson_interval(detection_count, int(direct["shots"]))
        for m in (1, 2, 4, 8):
            old = group[group["lambda"] == m].iloc[0]
            accept = single_accept**m
            detection = 1.0 - accept
            interval_low = 1.0 - (1.0 - direct_low) ** m
            interval_high = 1.0 - (1.0 - direct_high) ** m
            rows.append(
```

```
                {
                    "record_type": "swap_test",
                    "execution": execution,
                    "case": case,
                    "m": m,
                    "DIRECT_HARDWARE": "YES" if m == 1 else "NO",
                    "DERIVED_FROM_SINGLE_TEST": "NO" if m == 1 else "YES",
                    "backend": direct["backend"],
                    "job_id": direct["job_id"],
                    "shots": int(direct["shots"]),
                    "recomputed_accept_probability": accept,
                    "recomputed_detection_probability": detection,
                    "recomputed_wilson_95_low": interval_low,
                    "recomputed_wilson_95_high": interval_high,
                    "stored_probability": float(old["composed_detection_probability"]),
                    "absolute_difference": abs(detection - float(old["composed_detection_probability"])),
                    "composition_assumption": "direct m=1 hardware estimate" if m == 1 else "analytical independent-
identical composition from m=1 estimate",
                }
            )

    output_path.parent.mkdir(parents=True, exist_ok=True)
    with output_path.open("w", newline="", encoding="utf-8") as handle:
        writer = csv.DictWriter(handle, fieldnames=list(rows[0]))
        writer.writeheader()
        writer.writerows(rows)
```

ibm_single_qpu_candidate.py

```
#!/usr/bin/env python3
"""Maximum-feasible complete protocol-core candidate for one IBM QPU.

This circuit represents Alice, Bob, and Trent with separate logical registers on one
backend.  It implements three message preparations, three explicit Bell resources,
three coherent teleportation stages, duplicated encrypted Bell-outcome records,
strengthened-QOTP protection of the Trent reference copy, Trent/Bob swap tests, and
an explicit post-processed final decision.  It is not a networked three-QPU deployment,
does not implement QKD/authentication/arbitration, and implements one comparison copy
per circuit execution (lambda=1).
"""

from __future__ import annotations

import argparse
import csv
import hashlib
import json
import os
from collections import Counter
from datetime import datetime, timezone
from pathlib import Path
from typing import Any

import numpy as np
from qiskit import ClassicalRegister, QuantumCircuit, QuantumRegister
from qiskit.qasm3 import dumps as qasm3_dumps
from qiskit.transpiler import generate_preset_pass_manager

try:
    from qiskit_aer import AerSimulator
except ImportError:
    AerSimulator = None

try:
    from qiskit_ibm_runtime import QiskitRuntimeService, SamplerV2
except ImportError:
    QiskitRuntimeService = None
    SamplerV2 = None


X = np.array([[0, 1], [1, 0]], dtype=complex)
Y = np.array([[0, -1j], [1j, 0]], dtype=complex)
Z = np.array([[1, 0], [0, -1]], dtype=complex)
T_STRENGTHENED = np.sqrt(1j / 3) * (X - Y + Z)

KEYS = {
    "K_AT_p3": "0101",
    "K_AT_X1_bell_z": "0011",
    "K_AT_X1_bell_x": "1100",
    "K_AT_X1_comp_z": "0110",
    "K_AT_X1_comp_x": "1001",
    "K_BT_X2_bell_z": "1010",
    "K_BT_X2_bell_x": "0101",
    "K_BT_X2_comp_z": "1110",
    "K_BT_X2_comp_x": "0001",
    "K_BT_X3_bell_z": "1000",
    "K_BT_X3_bell_x": "0111",
    "K_BT_X3_comp_z": "0010",
    "K_BT_X3_comp_x": "1101",
}


def encryption_unitary(key4: str) -> np.ndarray:
    if len(key4) != 4 or set(key4) - {"0", "1"}:
        raise ValueError("Each strengthened-QOTP key block must contain four bits.")
    a, b, c, d = map(int, key4)
    return (
        np.linalg.matrix_power(X, a)
        @ np.linalg.matrix_power(Z, b)
        @ T_STRENGTHENED
        @ np.linalg.matrix_power(X, c)
        @ np.linalg.matrix_power(Z, d)
```

```python
    )


def append_qotp_roundtrip(qc: QuantumCircuit, qubit, key4: str, label: str) -> None:
    unitary = encryption_unitary(key4)
    qc.unitary(unitary, [qubit], label=f"{label}_enc")
    qc.unitary(unitary.conj().T, [qubit], label=f"{label}_dec")


def prepare_state(qc: QuantumCircuit, qubit, state: str) -> None:
    if state == "1":
        qc.x(qubit)
    elif state == "+":
        qc.h(qubit)
    elif state == "-":
        qc.x(qubit)
        qc.h(qubit)
    elif state != "0":
        raise ValueError("input_state must be one of 0, 1, +, -")


def return_to_zero_basis(qc: QuantumCircuit, qubit, state: str) -> None:
    if state in {"+", "-"}:
        qc.h(qubit)
    if state in {"1", "-"}:
        qc.x(qubit)


def prepare_bell_pair(qc: QuantumCircuit, sender, receiver, label: str) -> None:
    qc.h(sender)
    qc.cx(sender, receiver)
    qc.barrier(label=f"I2_{label}")


def coherent_teleport_with_duplicate_records(
    qc: QuantumCircuit,
    message,
    sender,
    receiver,
    bell_record,
    comp_record,
    key_prefix: str,
    stage: str,
) -> None:
    """Defer Bell measurements, coherently copy outcomes, and apply corrections."""
    qc.barrier(label=f"{stage}_Bell_transform")
    qc.cx(message, sender)
    qc.h(message)

    # Bell outcome ordering is (z outcome, x outcome) = (message, sender).
    for target in (bell_record[0], comp_record[0]):
        qc.cx(message, target)
    for target in (bell_record[1], comp_record[1]):
        qc.cx(sender, target)

    for suffix, qubit in (("bell_z", bell_record[0]), ("bell_x", bell_record[1]),
                          ("comp_z", comp_record[0]), ("comp_x", comp_record[1])):
        append_qotp_roundtrip(qc, qubit, KEYS[f"{key_prefix}_{suffix}"], f"{stage}_{suffix}")

    # Deferred-measurement form of X^x Z^z receiver correction.
    qc.cx(sender, receiver)
    qc.cz(message, receiver)
    qc.barrier(label=f"{stage}_receiver_corrected")


def append_swap_test(qc: QuantumCircuit, ancilla, left, right, stage: str) -> None:
    qc.barrier(label=f"{stage}_swap_test")
    qc.h(ancilla)
    qc.cswap(ancilla, left, right)
    qc.h(ancilla)


def build_complete_single_qpu_protocol(input_state: str = "+") -> QuantumCircuit:
    alice = QuantumRegister(3, "alice_msg")  # p1, p2, p3
    ab = QuantumRegister(2, "bell_AB")
```

```
    bt = QuantumRegister(2, "bell_BT")
    tb = QuantumRegister(2, "bell_TB")
    verify = QuantumRegister(2, "verify")  # Trent and Bob swap ancillas
    x1b = QuantumRegister(2, "X1_bell")
    x1c = QuantumRegister(2, "X1_comp")
    x2b = QuantumRegister(2, "X2_bell")
    x2c = QuantumRegister(2, "X2_comp")
    x3b = QuantumRegister(2, "X3_bell")
    x3c = QuantumRegister(2, "X3_comp")

    swap_bits = ClassicalRegister(2, "swap")
    message_bit = ClassicalRegister(1, "message")
    x1b_bits, x1c_bits = ClassicalRegister(2, "x1b"), ClassicalRegister(2, "x1c")
    x2b_bits, x2c_bits = ClassicalRegister(2, "x2b"), ClassicalRegister(2, "x2c")
    x3b_bits, x3c_bits = ClassicalRegister(2, "x3b"), ClassicalRegister(2, "x3c")

    qc = QuantumCircuit(
        alice, ab, bt, tb, verify, x1b, x1c, x2b, x2c, x3b, x3c,
        swap_bits, message_bit, x1b_bits, x1c_bits, x2b_bits, x2c_bits, x3b_bits, x3c_bits,
        name=f"complete_single_qpu_protocol_{input_state}",
    )

    # I1 is represented by fixed one-use key blocks in metadata; I2 and S1 are explicit.
    for qubit in alice:
        prepare_state(qc, qubit, input_state)
    qc.barrier(label="S1_three_independently_prepared_message_copies")
    prepare_bell_pair(qc, ab[0], ab[1], "AB")
    prepare_bell_pair(qc, bt[0], bt[1], "BT")
    prepare_bell_pair(qc, tb[0], tb[1], "TB")

    # S2-S3: Alice -> Bob, duplicate protected X1 records, and protected p3 reference.
    coherent_teleport_with_duplicate_records(qc, alice[0], ab[0], ab[1], x1b, x1c, "K_AT_X1", "S2_S3")
    p3_unitary = encryption_unitary(KEYS["K_AT_p3"])
    qc.unitary(p3_unitary, [alice[2]], label="S3_E_KAT_p3")

    # S4: Bob -> Trent and duplicate protected X2 records.
    coherent_teleport_with_duplicate_records(qc, ab[1], bt[0], bt[1], x2b, x2c, "K_BT_X2", "S4")

    # S5: Trent decrypts p3 and performs the comparison.
    qc.unitary(p3_unitary.conj().T, [alice[2]], label="S5_D_KAT_p3")
    append_swap_test(qc, verify[0], bt[1], alice[2], "S5_Trent")

    # S6-S7: Trent -> Bob, duplicate protected X3 records, then Bob comparison.
    coherent_teleport_with_duplicate_records(qc, bt[1], tb[0], tb[1], x3b, x3c, "K_BT_X3", "S6")
    append_swap_test(qc, verify[1], tb[1], alice[1], "S7_Bob")

    return_to_zero_basis(qc, tb[1], input_state)
    qc.measure(verify, swap_bits)
    qc.measure(tb[1], message_bit[0])
    qc.measure(x1b, x1b_bits); qc.measure(x1c, x1c_bits)
    qc.measure(x2b, x2b_bits); qc.measure(x2c, x2c_bits)
    qc.measure(x3b, x3b_bits); qc.measure(x3c, x3c_bits)

    qc.metadata = {
        "candidate_type": "complete_single_qpu_protocol_core",
        "input_state": input_state,
        "logical_roles": {"Alice": "alice_msg and AB sender", "Bob": "AB receiver and BT sender", "Trent": "BT receiver
and TB sender"},
        "implemented_steps": ["I1_fixed_key_metadata", "I2", "S1", "S2", "S3", "S4", "S5", "S6", "S7"],
        "implemented_checks": ["X1_duplicate_record_consistency", "X2_duplicate_record_consistency",
"X3_duplicate_record_consistency", "Trent_swap_test", "Bob_swap_test", "final_message_readout"],
        "lambda": 1,
        "decision_rule": "swap=00 and message=0 and x1b=x1c and x2b=x2c and x3b=x3c",
        "not_implemented": ["physical_node_separation", "inter_QPU_quantum_links", "QKD_execution",
"authenticated_session_binding", "dispute_arbitration", "lambda_greater_than_one_in_one_circuit"],
        "security_interpretation": "hardware execution evidence only; not a security proof",
    }
    return qc


def _counts_from_result(result: Any) -> dict[str, int]:
    if hasattr(result, "get_counts"):
        counts = result.get_counts()
        if isinstance(counts, list):
            counts = counts[0]
```

```python
        return {str(key): int(value) for key, value in counts.items()}
    if hasattr(result, "data") and hasattr(result.data, "keys"):
        register_names = list(result.data.keys())
        bit_arrays = [getattr(result.data, name) for name in register_names]
        if bit_arrays and all(hasattr(value, "get_bitstrings") for value in bit_arrays):
            shots = [value.get_bitstrings() for value in bit_arrays]
            if len({len(values) for values in shots}) != 1:
                raise RuntimeError("Runtime registers contain different shot counts.")
            # Match Qiskit's conventional count-key display: latest register first.
            return dict(Counter(" ".join(values[index] for values in reversed(shots)) for index in range(len(shots[0]))))
    for name in dir(result.data):
        if name.startswith("_"):
            continue
        value = getattr(result.data, name)
        if hasattr(value, "get_counts"):
            return {str(key): int(count) for key, count in value.get_counts().items()}
    raise RuntimeError("No result counts were found.")


def parse_register_key(key: str) -> dict[str, str]:
    fields = key.split()
    # Qiskit renders registers in reverse creation order.
    names = ["x3c", "x3b", "x2c", "x2b", "x1c", "x1b", "message", "swap"]
    if len(fields) != len(names):
        raise ValueError(f"Unexpected count key layout: {key!r}")
    return dict(zip(names, fields))


def is_success_key(key: str) -> bool:
    item = parse_register_key(key)
    return (
        item["swap"] == "00"
        and item["message"] == "0"
        and item["x1b"] == item["x1c"]
        and item["x2b"] == item["x2c"]
        and item["x3b"] == item["x3c"]
    )


def summarize_counts(counts: dict[str, int]) -> dict[str, Any]:
    shots = sum(counts.values())
    successes = sum(count for key, count in counts.items() if is_success_key(key))
    return {
        "shots": shots,
        "success_count": successes,
        "complete_core_acceptance_probability": successes / shots,
        "counts_json": json.dumps(counts, sort_keys=True),
    }


def component_metrics(counts: dict[str, int]) -> list[dict[str, Any]]:
    conditions = {
        "both_swap_tests_zero": lambda item: item["swap"] == "00",
        "message_readout_zero": lambda item: item["message"] == "0",
        "X1_duplicate_records_match": lambda item: item["x1b"] == item["x1c"],
        "X2_duplicate_records_match": lambda item: item["x2b"] == item["x2c"],
        "X3_duplicate_records_match": lambda item: item["x3b"] == item["x3c"],
        "complete_core_joint_acceptance": lambda item: is_success_key(" ".join(item[name] for name in ("x3c", "x3b",
"x2c", "x2b", "x1c", "x1b", "message", "swap"))),
    }
    shots = sum(counts.values())
    parsed = [(parse_register_key(key), count) for key, count in counts.items()]
    return [
        {"metric": name, "pass_count": sum(count for item, count in parsed if predicate(item)), "shots": shots,
         "probability": sum(count for item, count in parsed if predicate(item)) / shots}
        for name, predicate in conditions.items()
    ]


def circuit_sha256(circuit: QuantumCircuit) -> str:
    return hashlib.sha256(qasm3_dumps(circuit).encode("utf-8")).hexdigest()


def save_circuit_artifacts(circuit: QuantumCircuit, output_dir: Path, prefix: str) -> None:
    output_dir.mkdir(parents=True, exist_ok=True)
```

```
    (output_dir / f"{prefix}.qasm").write_text(qasm3_dumps(circuit), encoding="utf-8")
    (output_dir / f"{prefix}.txt").write_text(str(circuit.draw("text", fold=180)), encoding="utf-8")


def run_aer(output_dir: Path, shots: int, seed: int) -> dict[str, Any]:
    if AerSimulator is None:
        raise RuntimeError("qiskit-aer is required for Aer validation.")
    rows = []
    for state in ("0", "1", "+", "-"):
        logical = build_complete_single_qpu_protocol(state)
        simulator = AerSimulator()
        compiled = generate_preset_pass_manager(backend=simulator, optimization_level=1,
seed_transpiler=seed).run(logical)
        counts = simulator.run(compiled, shots=shots, seed_simulator=seed).result().get_counts()
        summary = summarize_counts(counts)
        rows.append({"input_state": state, "backend": "AerSimulator", "seed": seed, **summary})
        save_circuit_artifacts(logical, output_dir, f"complete_single_qpu_{state.replace('+', 'plus').replace('-',
'minus')}_logical")
    with (output_dir / "aer_complete_protocol_results.csv").open("w", newline="", encoding="utf-8") as handle:
        writer = csv.DictWriter(handle, fieldnames=list(rows[0])); writer.writeheader(); writer.writerows(rows)
    return {"rows": rows}


def runtime_service():
    if QiskitRuntimeService is None:
        raise RuntimeError("qiskit-ibm-runtime is not installed.")
    attempts = []
    try:
        return QiskitRuntimeService(name="entropy-project"), "saved_account"
    except Exception as exc:
        attempts.append(f"saved_account:{type(exc).__name__}")
    token = os.environ.get("QISKIT_IBM_TOKEN", "").strip()
    instance = os.environ.get("QISKIT_IBM_CRN", "").strip()
    if token and instance:
        return QiskitRuntimeService(channel="ibm_quantum_platform", token=token, instance=instance), "environment"
    raise RuntimeError("No callable IBM account was found (" + ", ".join(attempts) + "). No credential value was
printed.")


def submit_ibm(output_dir: Path, backend_name: str, shots: int, state: str, wait: bool) -> dict[str, Any]:
    if SamplerV2 is None:
        raise RuntimeError("qiskit-ibm-runtime is required for submission.")
    service, credential_source = runtime_service()
    backend = service.backend(backend_name)
    logical = build_complete_single_qpu_protocol(state)
    pass_manager = generate_preset_pass_manager(backend=backend, optimization_level=2, seed_transpiler=20260810)
    isa = pass_manager.run(logical)
    two_qubit = sum(value for gate, value in isa.count_ops().items() if gate in {"cz", "cx", "ecr", "rzz"})
    metadata = {
        "submitted_at": datetime.now(timezone.utc).isoformat(),
        "backend": backend.name,
        "shots": shots,
        "input_state": state,
        "logical_qubits": logical.num_qubits,
        "logical_clbits": logical.num_clbits,
        "logical_depth": logical.depth(),
        "logical_size": logical.size(),
        "logical_qasm_sha256": circuit_sha256(logical),
        "isa_qubits": isa.num_qubits,
        "isa_depth": isa.depth(),
        "isa_size": isa.size(),
        "isa_two_qubit_gates": two_qubit,
        "credential_source": credential_source,
        "claim_scope": "complete single-QPU protocol-core candidate; not distributed and not a security proof",
    }
    output_dir.mkdir(parents=True, exist_ok=True)
    save_circuit_artifacts(logical, output_dir, "complete_single_qpu_logical")
    save_circuit_artifacts(isa, output_dir, "complete_single_qpu_isa")
    sampler = SamplerV2(mode=backend)
    job = sampler.run([isa], shots=shots)
    metadata["job_id"] = job.job_id()
    (output_dir / "submission_metadata.json").write_text(json.dumps(metadata, indent=2), encoding="utf-8")
    print(json.dumps({key: metadata[key] for key in metadata if key != "credential_source"}, indent=2))
    if wait:
        result = job.result()[0]
```

```
        counts = _counts_from_result(result)
        summary = summarize_counts(counts)
        metadata["completed_at"] = datetime.now(timezone.utc).isoformat()
        metadata["status"] = str(job.status())
        metadata.update(summary)
        (output_dir / "raw_counts.json").write_text(json.dumps(counts, indent=2, sort_keys=True), encoding="utf-8")
        with (output_dir / "ibm_complete_protocol_result.csv").open("w", newline="", encoding="utf-8") as handle:
            writer = csv.DictWriter(handle, fieldnames=list(metadata)); writer.writeheader(); writer.writerow(metadata)
        (output_dir / "submission_metadata.json").write_text(json.dumps(metadata, indent=2), encoding="utf-8")
    return metadata


def retrieve_ibm_job(output_dir: Path, job_id: str) -> dict[str, Any]:
    service, credential_source = runtime_service()
    job = service.job(job_id)
    result = job.result()[0]
    counts = _counts_from_result(result)
    summary = summarize_counts(counts)
    metadata_path = output_dir / "submission_metadata.json"
    metadata = json.loads(metadata_path.read_text(encoding="utf-8")) if metadata_path.exists() else {}
    metadata.update({
        "job_id": job_id,
        "backend": job.backend().name,
        "status": str(job.status()),
        "completed_at": datetime.now(timezone.utc).isoformat(),
        "credential_source": credential_source,
        **summary,
    })
    try:
        metadata["runtime_metrics"] = job.metrics()
    except Exception as exc:
        metadata["runtime_metrics_error"] = type(exc).__name__
    output_dir.mkdir(parents=True, exist_ok=True)
    (output_dir / "raw_counts.json").write_text(json.dumps(counts, indent=2, sort_keys=True), encoding="utf-8")
    (output_dir / "submission_metadata.json").write_text(json.dumps(metadata, indent=2, default=str), encoding="utf-8")
    csv_row = {key: (json.dumps(value, sort_keys=True, default=str) if isinstance(value, (dict, list)) else value) for
key, value in metadata.items() if key != "credential_source"}
    with (output_dir / "ibm_complete_protocol_result.csv").open("w", newline="", encoding="utf-8") as handle:
        writer = csv.DictWriter(handle, fieldnames=list(csv_row)); writer.writeheader(); writer.writerow(csv_row)
    metrics = component_metrics(counts)
    with (output_dir / "ibm_complete_protocol_component_metrics.csv").open("w", newline="", encoding="utf-8") as handle:
        writer = csv.DictWriter(handle, fieldnames=list(metrics[0])); writer.writeheader(); writer.writerows(metrics)
    print(json.dumps({key: value for key, value in metadata.items() if key not in {"credential_source", "counts_json"}},
indent=2, default=str))
    return metadata


def main() -> None:
    parser = argparse.ArgumentParser()
    parser.add_argument("--mode", choices=["build", "aer", "submit", "retrieve"], default="build")
    parser.add_argument("--output-dir", type=Path, default=Path("complete_single_qpu_run"))
    parser.add_argument("--shots", type=int, default=1024)
    parser.add_argument("--seed", type=int, default=20260810)
    parser.add_argument("--backend", default="ibm_fez")
    parser.add_argument("--input-state", choices=["0", "1", "+", "-"], default="+")
    parser.add_argument("--confirm-submit", action="store_true")
    parser.add_argument("--wait", action="store_true")
    parser.add_argument("--job-id")
    args = parser.parse_args()

    if args.mode == "build":
        circuit = build_complete_single_qpu_protocol(args.input_state)
        save_circuit_artifacts(circuit, args.output_dir, "complete_single_qpu_logical")
        print(json.dumps({"qubits": circuit.num_qubits, "clbits": circuit.num_clbits, "depth": circuit.depth(), "size":
circuit.size(), "qasm_sha256": circuit_sha256(circuit), "metadata": circuit.metadata}, indent=2))
    elif args.mode == "aer":
        print(json.dumps(run_aer(args.output_dir, args.shots, args.seed), indent=2))
    elif args.mode == "submit":
        if not args.confirm_submit:
            raise RuntimeError("IBM submission requires --confirm-submit.")
        submit_ibm(args.output_dir, args.backend, args.shots, args.input_state, args.wait)
    else:
        if not args.job_id:
            raise RuntimeError("Retrieval requires --job-id.")
        retrieve_ibm_job(args.output_dir, args.job_id)
```

```
if __name__ == "__main__":
    main()
```

ibm_transpilation_artifacts.py

```
"""IBM ibm_fez ISA OpenQASM listings and export helper."""

from pathlib import Path

IBM_FEZ_TRANSPILED_QASM = {
    'COMPOSED_FULL_CANDIDATE_IBM_FEZ': r'''OPENQASM 3.0;
include "stdgates.inc";
bit[3] out;
barrier $105, $98, $108, $106, $107, $109, $118, $110, $111;
rz(pi/2) $106;
sx $106;
rz(pi/2) $106;
rz(pi/2) $107;
sx $107;
rz(pi) $107;
cz $106, $107;
sx $107;
rz(pi/2) $107;
barrier $105, $98, $108, $106, $107, $109, $118, $110, $111;
rz(pi/2) $109;
sx $109;
rz(pi/2) $109;
rz(pi/2) $118;
sx $118;
rz(pi) $118;
cz $109, $118;
sx $118;
rz(pi/2) $118;
barrier $105, $98, $108, $106, $107, $109, $118, $110, $111;
rz(pi/2) $110;
sx $110;
rz(pi/2) $110;
rz(pi/2) $111;
sx $111;
rz(pi) $111;
cz $110, $111;
sx $111;
rz(pi/2) $111;
barrier $105, $98, $108, $106, $107, $109, $118, $110, $111;
barrier $105, $98, $108, $106, $107, $109, $118, $110, $111;
rz(pi/2) $106;
sx $106;
rz(-pi) $106;
cz $105, $106;
rz(-pi) $105;
sx $105;
rz(pi/2) $105;
sx $106;
rz(pi/2) $106;
rz(pi/2) $107;
sx $107;
rz(pi) $107;
cz $106, $107;
sx $106;
cz $106, $105;
sx $105;
sx $106;
cz $106, $105;
sx $105;
sx $106;
cz $106, $105;
sx $107;
rz(pi/2) $107;
cz $106, $107;
barrier $106, $98, $108, $105, $107, $109, $118, $110, $111;
reset $106;
sx $107;
sx $108;
cz $108, $107;
sx $107;
sx $108;
cz $108, $107;
sx $107;
```

```
sx $108;
cz $108, $107;
rz(pi/2) $109;
sx $109;
cz $108, $109;
rz(pi/2) $108;
sx $108;
rz(pi/2) $108;
sx $109;
rz(-1.1252209778447106) $109;
sx $109;
sx $118;
rz(-pi/2) $118;
cz $109, $118;
rz(-pi) $109;
sx $109;
rz(-pi/2) $109;
sx $118;
cz $109, $118;
sx $109;
cz $108, $109;
sx $118;
rz(0.4455753489501859) $118;
barrier $106, $98, $107, $105, $108, $118, $109, $110, $111;
rz(pi/2) $106;
sx $106;
rz(5*pi/4) $106;
x $107;
rz(pi/2) $108;
sx $108;
sx $109;
rz(-3*pi/2) $109;
cz $108, $109;
sx $108;
sx $109;
cz $108, $109;
rz(-pi) $108;
sx $108;
sx $109;
cz $108, $109;
sx $108;
rz(-3*pi/2) $108;
cz $107, $108;
rz(-pi) $107;
sx $107;
rz(pi/4) $107;
sx $107;
cz $106, $107;
sx $107;
rz(5*pi/4) $107;
sx $107;
rz(pi) $107;
sx $108;
rz(-5*pi/4) $108;
cz $108, $107;
sx $107;
rz(3*pi/4) $107;
sx $107;
rz(pi) $107;
cz $106, $107;
sx $107;
rz(-pi/4) $107;
sx $107;
rz(pi/2) $107;
cz $107, $108;
sx $107;
sx $108;
cz $107, $108;
sx $107;
sx $108;
cz $107, $108;
cz $106, $107;
sx $107;
rz(3*pi/4) $107;
sx $107;
```

```
rz(pi) $107;
cz $106, $107;
sx $106;
rz(pi/2) $106;
cz $108, $107;
sx $107;
rz(pi/2) $107;
rz(pi/2) $109;
barrier $106, $98, $108, $105, $109, $118, $107, $110, $111;
sx $107;
sx $108;
cz $108, $107;
sx $107;
sx $108;
cz $108, $107;
sx $107;
sx $108;
cz $108, $107;
reset $109;
rz(pi/2) $109;
sx $109;
sx $110;
rz(-3*pi/2) $110;
cz $109, $110;
sx $109;
sx $110;
cz $109, $110;
rz(-pi) $109;
sx $109;
rz(-pi/2) $109;
sx $110;
cz $109, $110;
cz $108, $109;
rz(-pi) $108;
sx $108;
rz(pi/2) $108;
sx $109;
rz(-pi/2) $109;
rz(pi/2) $110;
sx $110;
sx $111;
cz $110, $111;
sx $110;
sx $111;
cz $110, $111;
sx $110;
sx $111;
cz $110, $111;
rz(-pi/2) $110;
sx $110;
rz(pi) $110;
cz $109, $110;
sx $109;
cz $109, $108;
sx $108;
sx $109;
cz $109, $108;
sx $108;
sx $109;
cz $109, $108;
sx $110;
rz(-pi/2) $110;
cz $109, $110;
barrier $106, $98, $107, $105, $111, $118, $109, $108, $110;
sx $98;
sx $110;
rz(-pi) $111;
sx $111;
rz(pi/2) $111;
cz $111, $98;
sx $98;
sx $111;
cz $111, $98;
sx $98;
sx $111;
```

```
cz $111, $98;
cz $111, $110;
rz(-pi) $110;
sx $110;
rz(-5*pi/4) $110;
sx $111;
rz(-3*pi/4) $111;
sx $111;
cz $98, $111;
sx $111;
rz(5*pi/4) $111;
sx $111;
rz(pi) $111;
cz $110, $111;
sx $111;
rz(3*pi/4) $111;
sx $111;
rz(pi) $111;
cz $98, $111;
sx $111;
rz(-pi/4) $111;
sx $111;
rz(pi/2) $111;
cz $111, $110;
sx $110;
sx $111;
cz $111, $110;
sx $110;
sx $111;
cz $111, $110;
cz $98, $111;
rz(3*pi/4) $98;
sx $111;
rz(3*pi/4) $111;
sx $111;
rz(pi) $111;
cz $98, $111;
sx $98;
rz(pi/2) $98;
cz $110, $111;
sx $111;
out[0] = measure $106;
out[1] = measure $98;
out[2] = measure $111;
''',
    'SWAP_TEST_ORTHOGONAL_0_1_IBM_FEZ': r'''OPENQASM 3.0;
include "stdgates.inc";
bit[1] c;
rz(pi/2) $145;
sx $145;
rz(5*pi/4) $145;
sx $147;
cz $146, $147;
rz(-pi) $146;
sx $146;
rz(pi/4) $146;
sx $146;
cz $145, $146;
sx $146;
rz(5*pi/4) $146;
sx $146;
rz(pi) $146;
sx $147;
rz(-5*pi/4) $147;
cz $147, $146;
sx $146;
rz(3*pi/4) $146;
sx $146;
rz(pi) $146;
cz $145, $146;
sx $146;
rz(-pi/4) $146;
sx $146;
rz(pi/2) $146;
cz $146, $147;
```

```
sx $146;
sx $147;
cz $146, $147;
sx $146;
sx $147;
cz $146, $147;
cz $145, $146;
sx $146;
rz(3*pi/4) $146;
sx $146;
rz(pi) $146;
cz $145, $146;
sx $145;
rz(pi/2) $145;
cz $147, $146;
sx $146;
rz(pi/2) $146;
c[0] = measure $145;
''',
    'SWAP_TEST_IDENTICAL_0_0_IBM_FEZ': r'''OPENQASM 3.0;
include "stdgates.inc";
bit[1] c;
rz(pi/2) $145;
sx $145;
rz(5*pi/4) $145;
sx $147;
cz $146, $147;
sx $146;
rz(-3*pi/4) $146;
sx $146;
cz $145, $146;
sx $146;
rz(5*pi/4) $146;
sx $146;
rz(pi) $146;
rz(-pi) $147;
sx $147;
rz(-5*pi/4) $147;
cz $147, $146;
sx $146;
rz(3*pi/4) $146;
sx $146;
rz(pi) $146;
cz $145, $146;
sx $146;
rz(-pi/4) $146;
sx $146;
rz(pi/2) $146;
cz $146, $147;
sx $146;
sx $147;
cz $146, $147;
sx $146;
sx $147;
cz $146, $147;
cz $145, $146;
sx $146;
rz(3*pi/4) $146;
sx $146;
rz(pi) $146;
cz $145, $146;
sx $145;
rz(pi/2) $145;
cz $147, $146;
sx $146;
rz(pi/2) $146;
c[0] = measure $145;
''',
    'SWAP_TEST_IDENTICAL_PLUS_PLUS_IBM_FEZ': r'''OPENQASM 3.0;
include "stdgates.inc";
bit[1] c;
rz(pi/2) $145;
sx $145;
rz(5*pi/4) $145;
rz(-pi/2) $146;
```

```
sx $146;
rz(3*pi/2) $146;
rz(-pi) $147;
sx $147;
rz(-3*pi/2) $147;
cz $146, $147;
sx $146;
rz(pi/4) $146;
sx $146;
cz $145, $146;
sx $146;
rz(5*pi/4) $146;
sx $146;
rz(pi) $146;
sx $147;
rz(-5*pi/4) $147;
cz $147, $146;
sx $146;
rz(3*pi/4) $146;
sx $146;
rz(pi) $146;
cz $145, $146;
sx $146;
rz(-pi/4) $146;
sx $146;
rz(pi/2) $146;
cz $146, $147;
sx $146;
sx $147;
cz $146, $147;
sx $146;
sx $147;
cz $146, $147;
cz $145, $146;
sx $146;
rz(3*pi/4) $146;
sx $146;
rz(pi) $146;
cz $145, $146;
sx $145;
rz(pi/2) $145;
cz $147, $146;
sx $146;
rz(pi/2) $146;
c[0] = measure $145;
''',
    'SWAP_TEST_ORTHOGONAL_PLUS_MINUS_IBM_FEZ': r'''OPENQASM 3.0;
include "stdgates.inc";
bit[1] c;
rz(pi/2) $145;
sx $145;
rz(5*pi/4) $145;
rz(-pi/2) $146;
sx $146;
rz(-pi/2) $146;
sx $147;
cz $146, $147;
sx $146;
rz(pi/4) $146;
sx $146;
cz $145, $146;
sx $146;
rz(5*pi/4) $146;
sx $146;
rz(pi) $146;
rz(-pi/2) $147;
sx $147;
rz(-pi/4) $147;
cz $147, $146;
sx $146;
rz(3*pi/4) $146;
sx $146;
rz(pi) $146;
cz $145, $146;
sx $146;
```

```
rz(-pi/4) $146;
sx $146;
rz(pi/2) $146;
cz $146, $147;
sx $146;
sx $147;
cz $146, $147;
sx $146;
sx $147;
cz $146, $147;
cz $145, $146;
sx $146;
rz(3*pi/4) $146;
sx $146;
rz(pi) $146;
cz $145, $146;
sx $145;
rz(pi/2) $145;
cz $147, $146;
sx $146;
rz(pi/2) $146;
c[0] = measure $145;
''',
    'SWAP_TEST_FIXED_OVERLAP_F_0_25_IBM_FEZ': r'''OPENQASM 3.0;
include "stdgates.inc";
bit[1] c;
rz(pi/2) $145;
sx $145;
rz(5*pi/4) $145;
sx $146;
rz(-pi/3) $146;
sx $146;
rz(-pi) $147;
sx $147;
rz(-pi) $147;
cz $146, $147;
sx $146;
rz(-3*pi/4) $146;
sx $146;
cz $145, $146;
sx $146;
rz(5*pi/4) $146;
sx $146;
rz(pi) $146;
sx $147;
rz(-pi/4) $147;
cz $147, $146;
sx $146;
rz(3*pi/4) $146;
sx $146;
rz(pi) $146;
cz $145, $146;
sx $146;
rz(-pi/4) $146;
sx $146;
rz(pi/2) $146;
cz $146, $147;
sx $146;
sx $147;
cz $146, $147;
sx $146;
sx $147;
cz $146, $147;
cz $145, $146;
sx $146;
rz(3*pi/4) $146;
sx $146;
rz(pi) $146;
cz $145, $146;
sx $145;
rz(pi/2) $145;
cz $147, $146;
sx $146;
rz(pi/2) $146;
c[0] = measure $145;
```

```
''',
}


def write_transpiled_qasm(output_dir: Path) -> None:
    """Write each included target-transpiled program without changing its instructions."""
    output_dir.mkdir(parents=True, exist_ok=True)
    for name, source in IBM_FEZ_TRANSPILED_QASM.items():
        (output_dir / f'{name}.qasm').write_text(source, encoding='utf-8')


if __name__ == "__main__":
    write_transpiled_qasm(Path('ibm_fez_transpiled_qasm'))
```
